# The private life of *Cystodinium*: *in situ* observation of its attachments and population dynamics

**DOI:** 10.1093/plankt/fbab025

**Published:** 2021-04-19

**Authors:** Tara Tapics, Irene Gregory-Eaves, Yannick Huot

**Affiliations:** Département de géomatique appliquée, Université de Sherbrooke, 2500, boul. de l’Université, Sherbrooke, Québec J1K 2R1, Canada; Department of Biology, McGill University, 845 Sherbrooke Street West, Montréal, Québec H3A 0G4, Canada; Département de géomatique appliquée, Université de Sherbrooke, 2500, boul. de l’Université, Sherbrooke, Québec J1K 2R1, Canada

**Keywords:** *Cystodinium*, dinoflagellates, cysts, parasites, freshwater, imaging flow cytometer, vegetative cysts, non-motile stage, temporary cysts, coccoid stage

## Abstract

Phytoplankton images were collected using an Imaging Flow Cytobot moored in the mesotrophic lake Lac Montjoie (Quebec, Canada). *Cystodinium*—an unusual dinoflagellate genus—was found during manual classification of the images into taxonomic groups while building an automated classifier. *Cystodinium*’s particularity is that while it can take a typical motile dinoflagellate form, it is thought to exist primarily as an immotile photosynthetically competent parasitic cyst in the shape of a crescent moon. Observations presented here are of this immotile lunate cyst. Manually classified images revealed that the majority of the *Cystodinium* found (86%) were attached to other microalgae or detrital material while the rest were unattached. The established auto-classifier was only able to correctly identify unattached *Cystodinium* images and thus was used to generate time series as cells per 100 mL for the unattached cell subset. Our observations, coupled with a literature review, lead us to question the parasitic nature of this taxonomic group.

## INTRODUCTION

Mixotrophic freshwater dinoflagellates are very poorly studied (Sanders, 2011) and *Cystodinium* is no exception. *Cystodinium* has typically been described as a mixotrophic parasite of microalgae (e.g. Cachon and Cachon, 1987; Gómez, 2012; Stoecker, 1999), that spends most of its life as an immotile lunate cyst (Taylor, 1987a, p. 12, citing Klebs 1912; Taylor, 1987b, p. 42). This description places it among the few (up to 7%) freshwater dinoflagellates considered to be parasites (Gómez, 2012). It was originally believed to be an obligate autotroph (Cachon and Cachon, 1987, p. 572).

The life cycle, habitat and taxonomy of *Cystodinium* remain understudied. *Cystodinium* was first described as having “free-floating” lunate cells (cysts) and motile “*Gymnodinium*-like” gametes (Prescott, 1962, pp. 438–439, citing Klebs 1912). *Cystodinium*’s life history has been characterized only once, for the holotype species *Cystodinium bataviense*: *in vitro* cell division in the lunate immotile cell (what we call cysts) was reportedly followed by the release of short-lived, flagellated *Gymnodinium*-like phototactic gametes that rapidly swam to the air–water interface, then “colonized” the epineuston as immotile cells (Timpano and Pfiester, 1985). However, *Cystodinium* (which is almost always reported in its cyst form) is more typically described as benthic (Gómez, 2012) or tychoplanktonic (Prescott, 1962, p. 439). Because they are poorly described, Thompson and Meyer (1984) suggested the removal of numerous *Cystodinium* species from taxonomic lists. More recently, the genus was called “insufficiently known” (Gómez, 2012).

The lunate *Cystodinium* cyst is often observed attached to other microalgae via a specialized holdfast or peduncle, hence their reputation as ectoparasites. The nature of their peduncle does not appear to have been studied and we could not find evidence in the literature of heterotrophic feeding behavior via the peduncle. Nutritionally independent and phototrophic “ectoparasitic” dinoflagellates whose peduncles appear to be mere holdfasts do exist (Schnepf and Elbrächter, 1992).

*Cystodinium*’s status as a parasite seems to have been established by Pfiester and Lynch (1980), who observed “pseudopodia” extending from the (previously unobserved) “amoeboid” life stages of *C. bataviense* into the cells of *Oedogonium* and consuming them over a period of 10–12 h. We did not find other observations of the amoeboid *Cystodinium* life stage in the literature. Unfortunately, the accompanying images do not allow for the verification of the consumption of *Oedogonium* by *Cystodinium*. Other researchers who have studied the genus or have it in culture agree that there is insufficient evidence that *Cystodinium* is parasitic (A. J. Calado, Aveiro and Ø. Moestrup, Copenhagen, personal communication). Herein, we report on *in situ* observations of the lunate *Cystodinium* cyst and its attachments with the objective of advancing our understanding of the ecology of this taxon.

## METHOD

We used an Imaging Flow Cytobot (IFCB) (Olson and Sosik, 2007) moored in a deep central portion of Lac Montjoie (latitude: 45.4092, longitude: −72.0995), Quebec, Canada to image chlorophyll-a fluorescing particles between ~10 and 100 μm. Samples of ~4 mL were drawn, and the particles were imaged roughly every 25 min while the instrument was operational (see Supplemental Fig. S1 for periods of operation; sample flow rate, 0.25 mL min^−1^; cell transit velocity through flow cell, 2.2 ms^−1^). The instrument profiled the water column, but the pattern of sampling depths changed through time (see Supplemental Fig. S2 for sampling depth details). A total of 31,990 samples were imaged from late 2012 through 2016.

We reviewed images from 4270 samples to select representative photographs for various plankton groups found in the lake and build an automated classifier (Sosik and Olson, 2007). *Cystodinium* cysts posed a number of challenges to our “hard” automated classifier: (i) they were exceedingly rare, limiting the number of training images, and (ii) they often appeared attached to other plankton or detritus. Since a “hard” classifier assigns each image to one plankton category, images containing two or more taxa cannot be classified properly. We therefore examined *Cystodinium’s in situ* associations using 325 manually identified images. Although these images provide useful insights, we cannot provide robust quantitative estimates or seasonal trends with these data. Time series could be generated of unattached *Cystodinium* cysts with the auto-classifier, but this process produced false positives. Thus, we visually reviewed the auto-classified *Cystodinium* cysts from the year 2013 (9664 files) to exclude other taxa. Note that in addition to strictly unattached *Cystodinium* cysts, the automated classifier catches *Cystodinium* cysts with low visibility attachments, and we kept these in the auto-classified set. Overall, we had 446 confirmed detections with the auto-classifier and an additional 198 detections in the manually identified set.

## RESULT

**Fig. 1:**
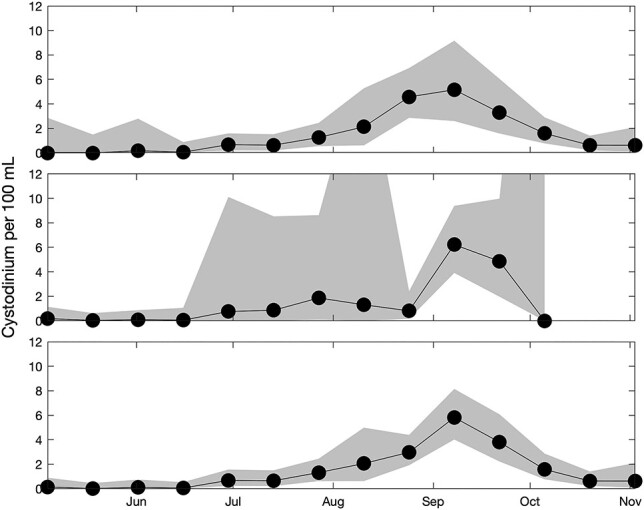
Upper plot: distribution of unattached *Cystodinium* cyst detections per 100 mL volume above the 7-day average seasonal thermocline. Middle plot: distribution of unattached *Cystodinium* cyst detections per 100 mL volume below the 7-day average seasonal thermocline. Lower plot: distribution of unattached *Cystodinium* cyst detections per 100 mL volume over all available depths. Two weeks' data are pooled into each Cystodinium per 100 mL value: the black circles mark the midpoints of the intervals (midpoints at 4-May, 18-May, 1-Jun, 15-Jun, 29-Jun, 13-Jul, 27-Jul, 10-Aug, 24-Aug, 7-Sep, 21-Sep, 5-Oct, 19-Oct, 2-Nov). 95% confidence intervals appear in gray; upper confidence limits that exceed the plotted range correspond to regions of very low sampling intensity (first point in May 2013 above thermocline, upper confidence limit 123, last point in October 2013 below thermocline, upper confidence limit 46).

The time series of auto-classified, unattached *Cystodinium* cysts per 100 mL showed a peak in September 2013 and relatively low concentrations in May and June (Fig. 1). The manually identified *Cystodinium* cysts showed a similar pattern in 2013 and increasing abundance in July 2014 (Supplemental Fig. S1).

*Cystodinium* was attached in 281 of the 325 manually identified cyst images (86%). No attachment was visible in the remaining 44 images, which suggests that it spends ~14% of its lunate cyst stage as free living. Alternatively, unattached cases represent images where the attachment is to transparent/invisible material or where the cysts detached in the IFCB (we consider detachment unlikely).

**Fig. 2:**
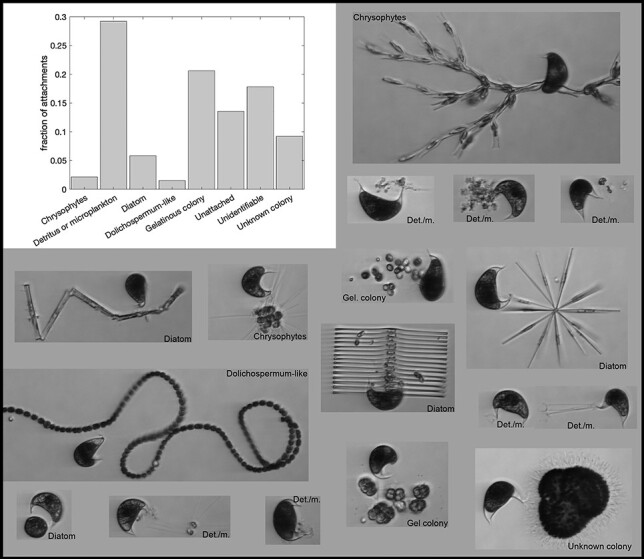
Fraction of *Cystodinium* cysts found either unattached, or attached to a particular “host”; the categories “Unknown colony” and “Gelatinous colony” each encompass several different taxa that could not be identified beyond their functional group. The category “Diatoms” includes *Asterionella*, *Fragilaria*, *Synedra*, *Tabellaria* and an unidentified centric diatom. “Detritus/microplankton” includes images of material that was either clearly detrital or composed of fine agglomerates that could have been detritus or microplankton. “Unidentifiable” includes all images for which either (i) a point of attachment (generally to something transparent and gelatinous) was visible but the host was not, or (ii) a few cells were visible but no identification could be made. See supplemental material for full image set.

Attached *Cystodinium* cysts were commonly associated with detrital material/flocs (34%; Fig. 2). Sixty other images (21%) showed attachments that were not clear enough to be classified. The remainder (46%) showed attachments to individual microalgal cells or colonies, although some of these could only be identified to functional group (e.g. gelatinous colonies). There was no discernable pattern in the detections with respect to the time of the day (results not shown).

## DISCUSSION

Data from the IFCB revealed previously unobserved patterns in the occurrence of unattached *Cystodinium* cysts, which were found at low levels over all depths and throughout the sampling period. The IFCB also provided a unique look at the *in situ* hosts of the attached *Cystodinium* cysts, which included a variety of microalgal taxa as well as detritus. The depth distribution of our *Cystodinium* detections do not support inferences that the cysts are primarily benthic/tychoplanktonic (e.g. Prescott, 1962, p. 439) nor epineustonic (e.g. Timpano and Pfiester, 1985). Unattached *Cystodinium* cysts peaked under conditions that would not tend to favor growth in autotrophs (i.e. as light was diminishing in the fall when the thermocline was deepening but had not yet overturned) nor microalgal parasites (i.e. during a period that did not coincide with peaks in its microalgal hosts).

## CONCLUSION

Our data do not allow us to draw further conclusions about the nature of *Cystodinium* cysts. However, as Gómez, 2012 (citing Baumeister 1957) suggests, *Cystodinium* may be a “form genus” (a genus that was named when a morphologically distinct life stage was seen but not linked to the other life stages). The *Gymnodinium*-like cell could in fact be the primary form. *Cystodinium* cysts are highly identifiable, while *Gymnodinium* are morphologically difficult to distinguish (e.g. Hoppenrath, 2016)—*Gymnodinium*-like swimming *Cystodinium* could be improperly recorded in plankton records (especially if no one is looking for them). The seasonal pattern we observed in *Cystodinium* cysts seems more consistent with a resting-phase role, particularly if the cysts are first formed continuously at low levels as a form of bet-hedging, then in greater numbers as winter approaches.

## Supplementary Material

Cystodinium_and_its_attachments-Dolichospermum_label_fbab025

Cystodinium_supplemental_2021_submission_fbab025
