# Supplementary material for: The private life of Cystodinium: in situ observation of its attachments and population dynamics
Source: J Plankton Res. 2021 Apr 19;43(3):492–6. doi: 10.1093/plankt/fbab025 (PMC8163037; doi:10.1093/plankt/fbab025)

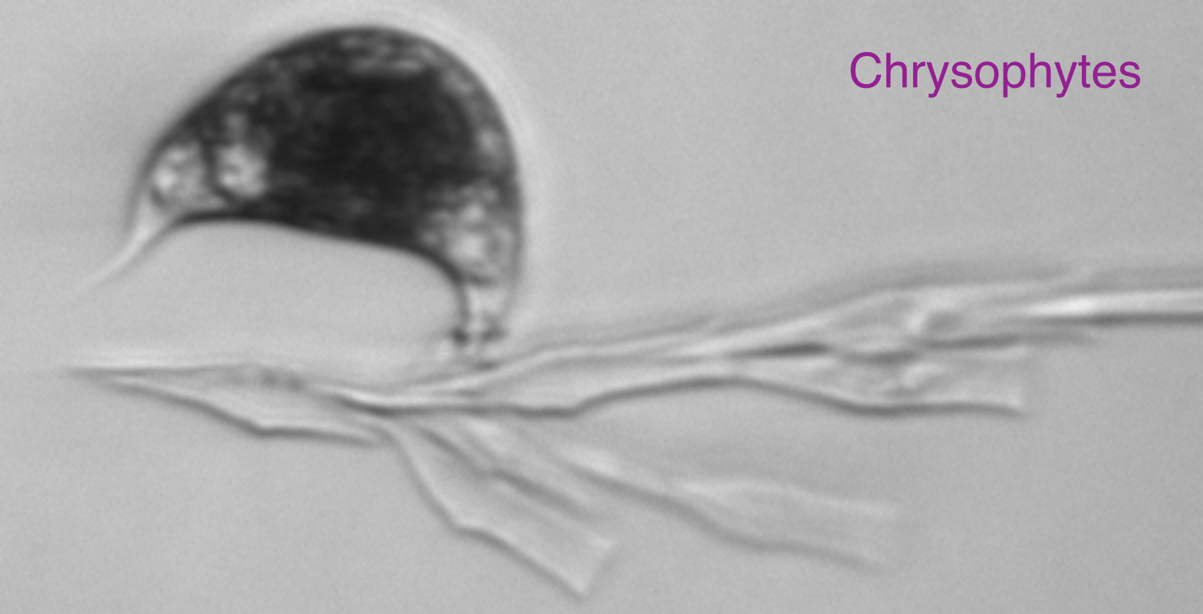
Chrysophytes

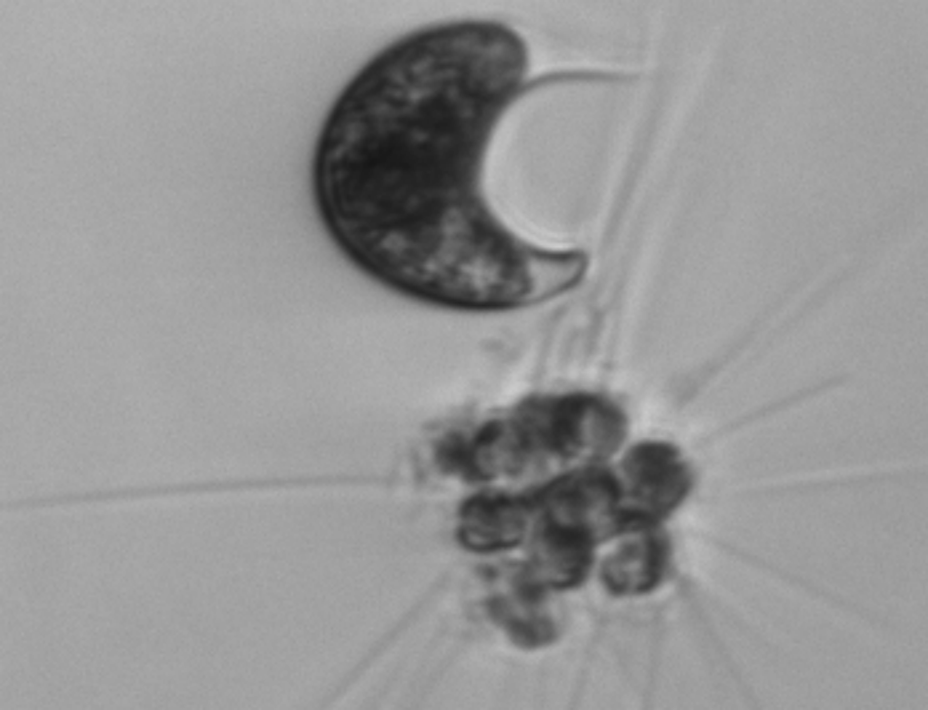

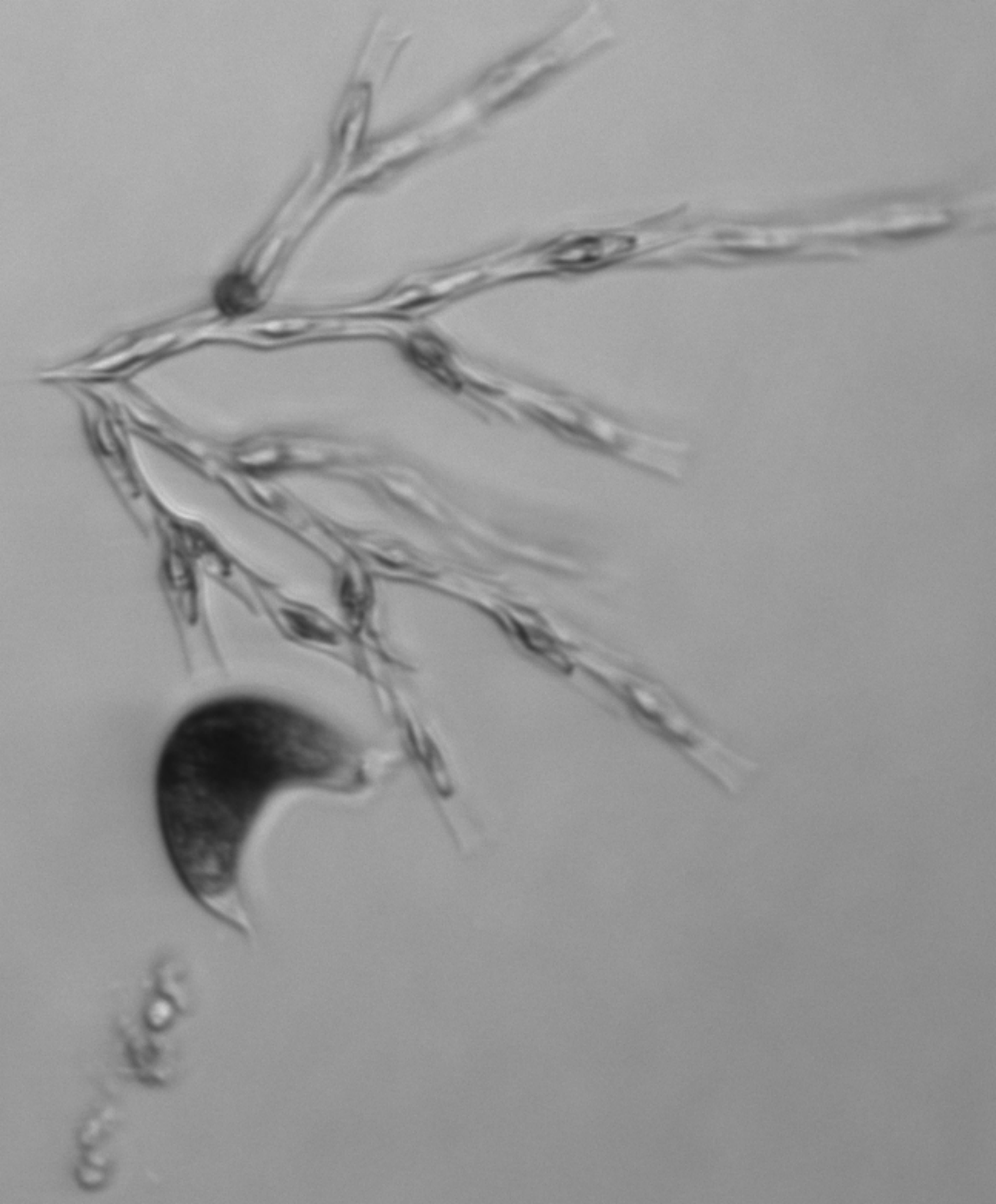

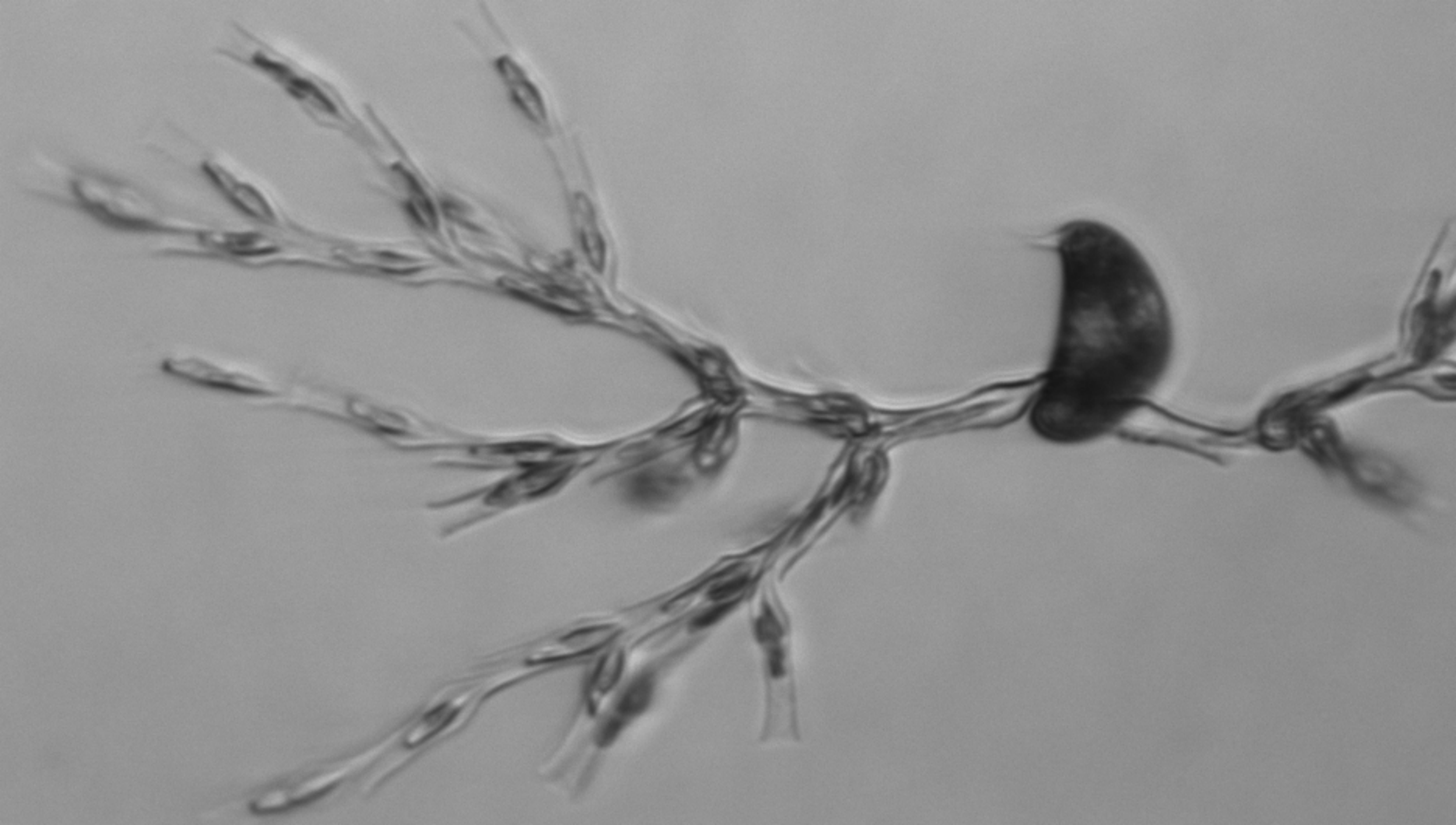

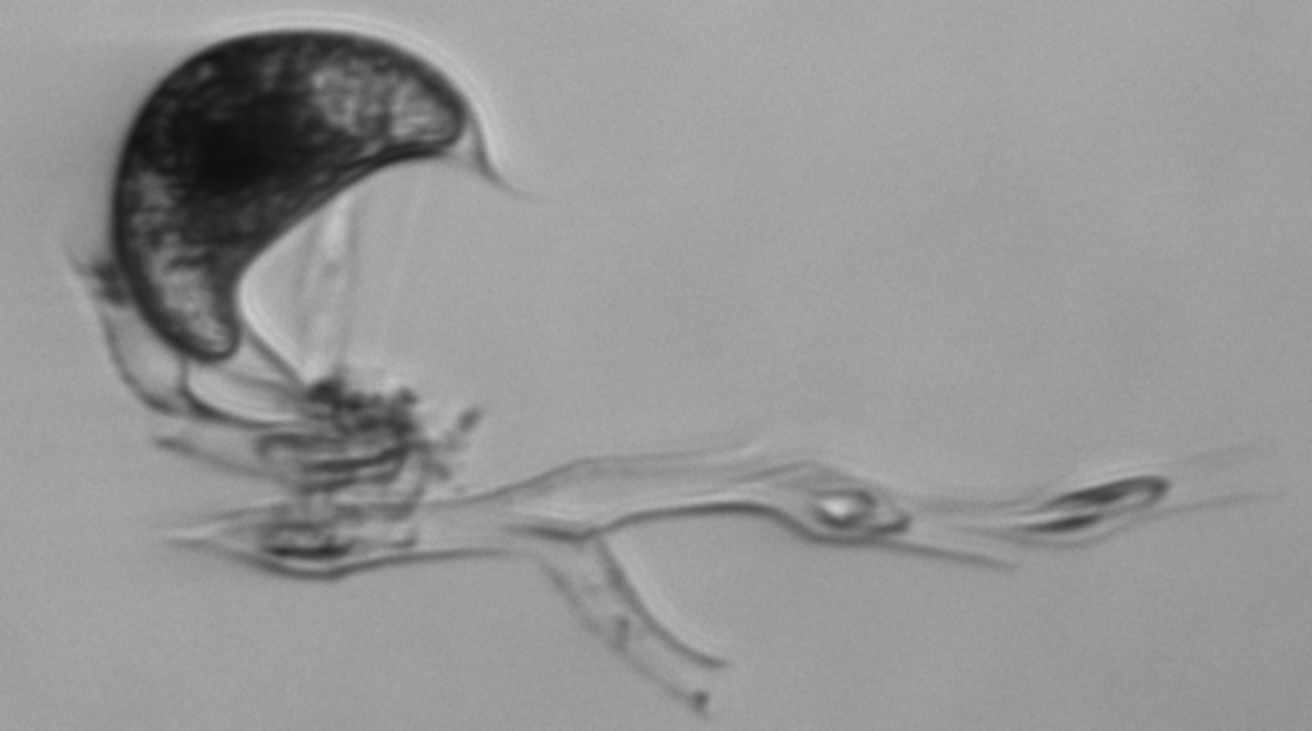

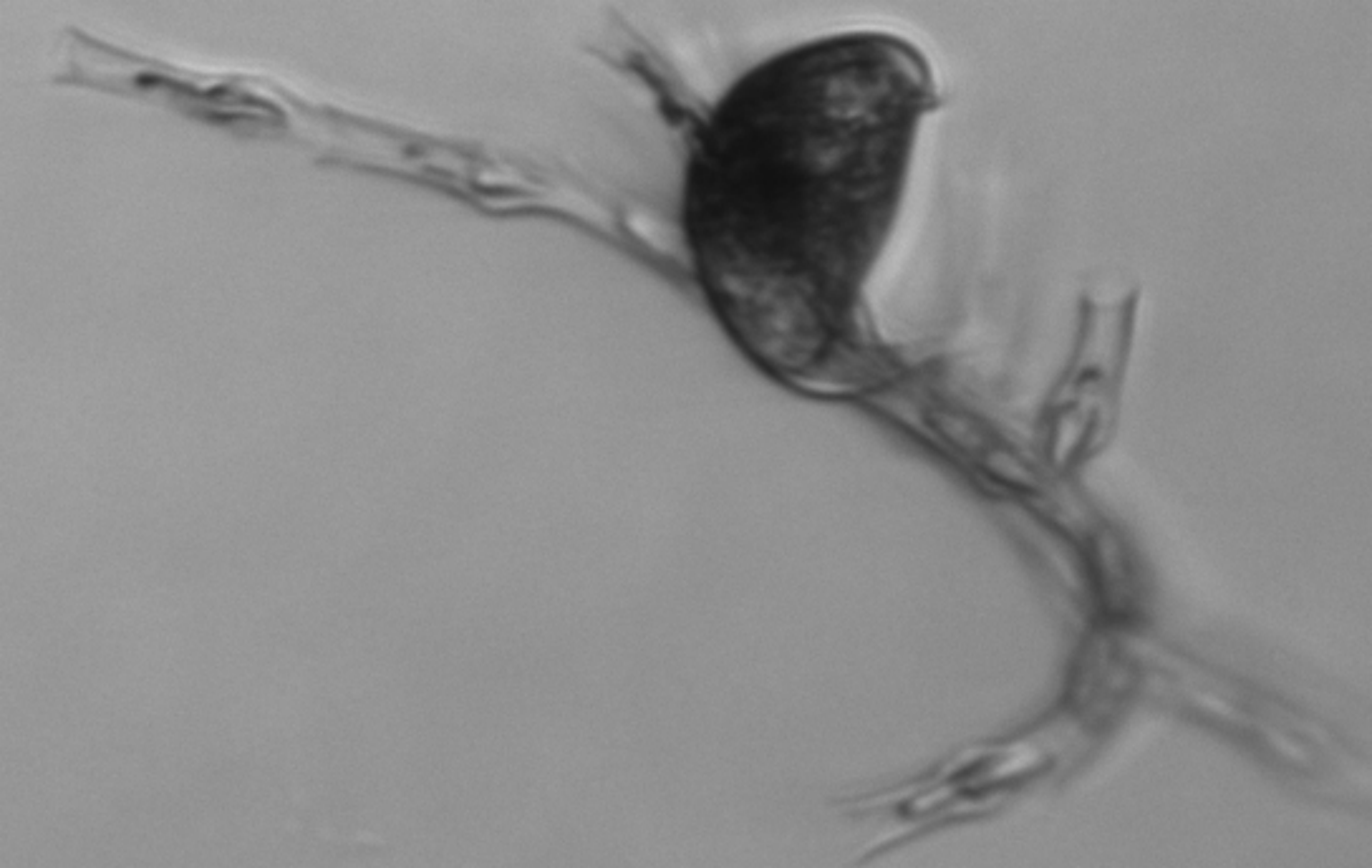

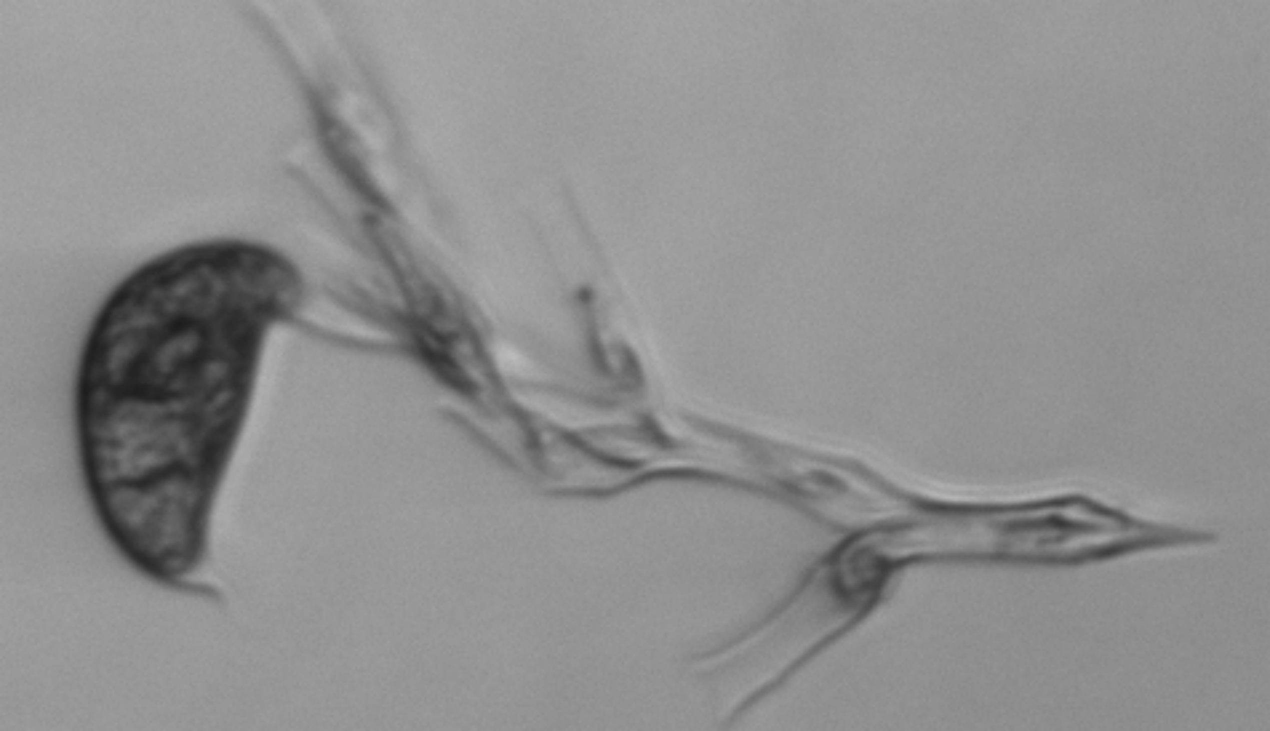

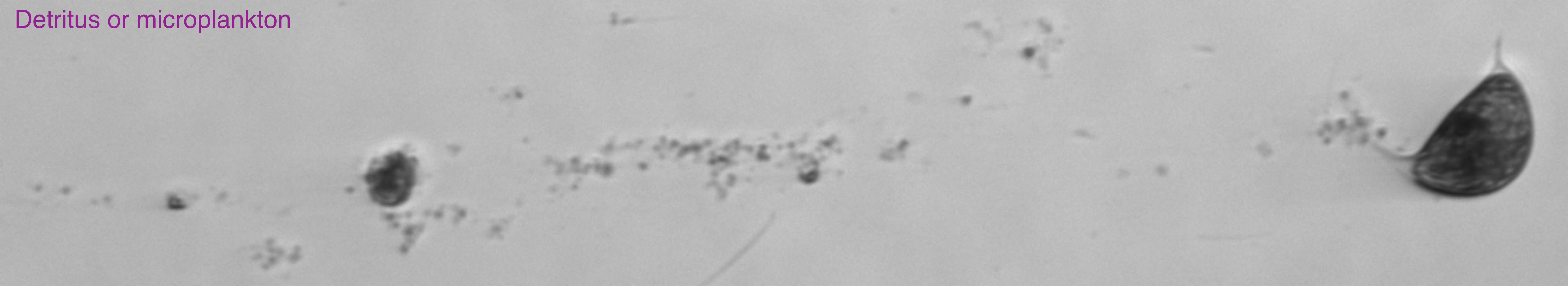
Detritus or microplankton

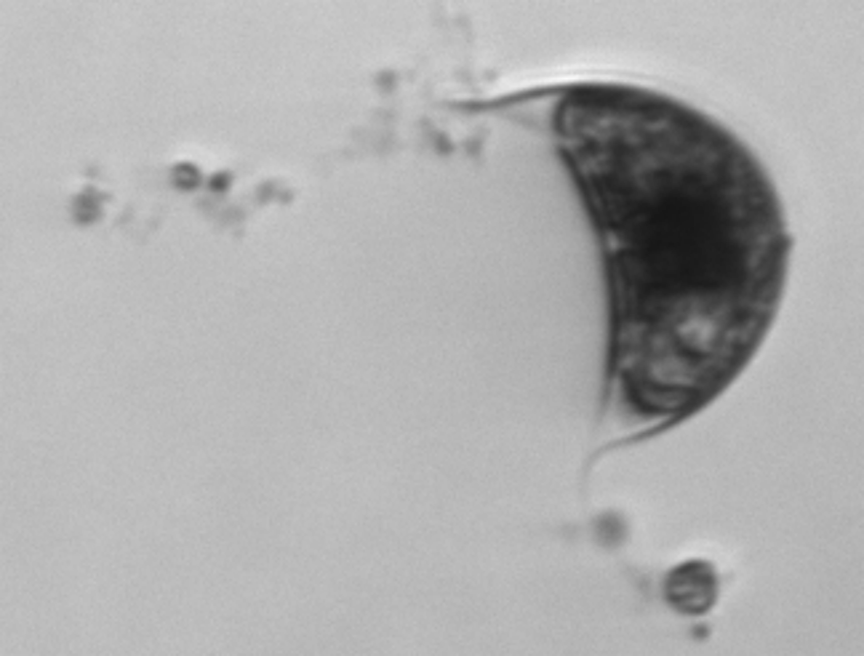

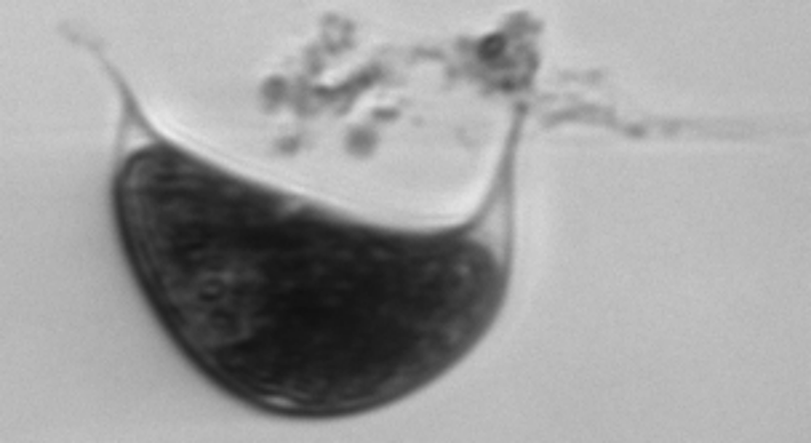

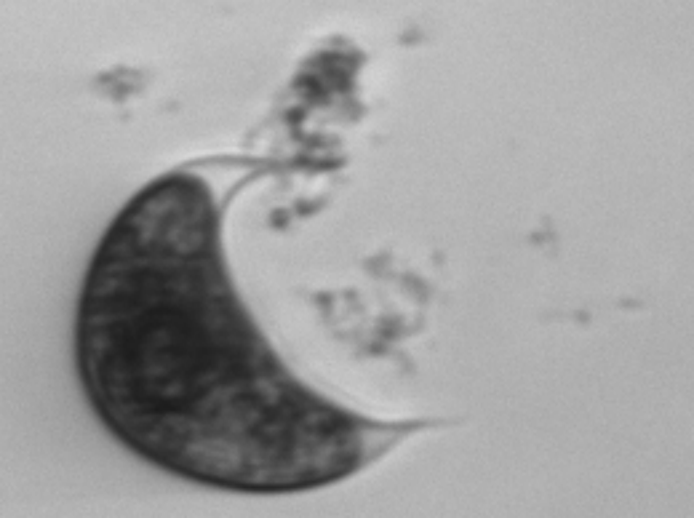

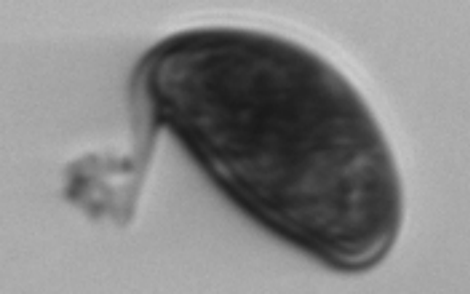

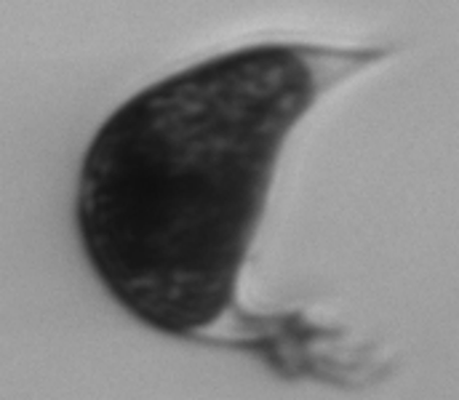

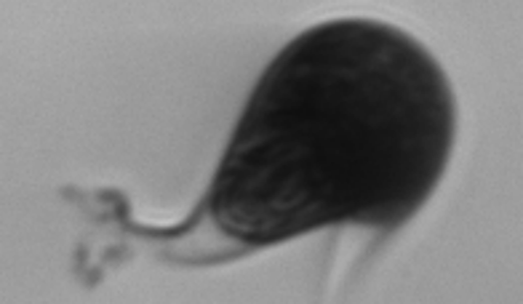

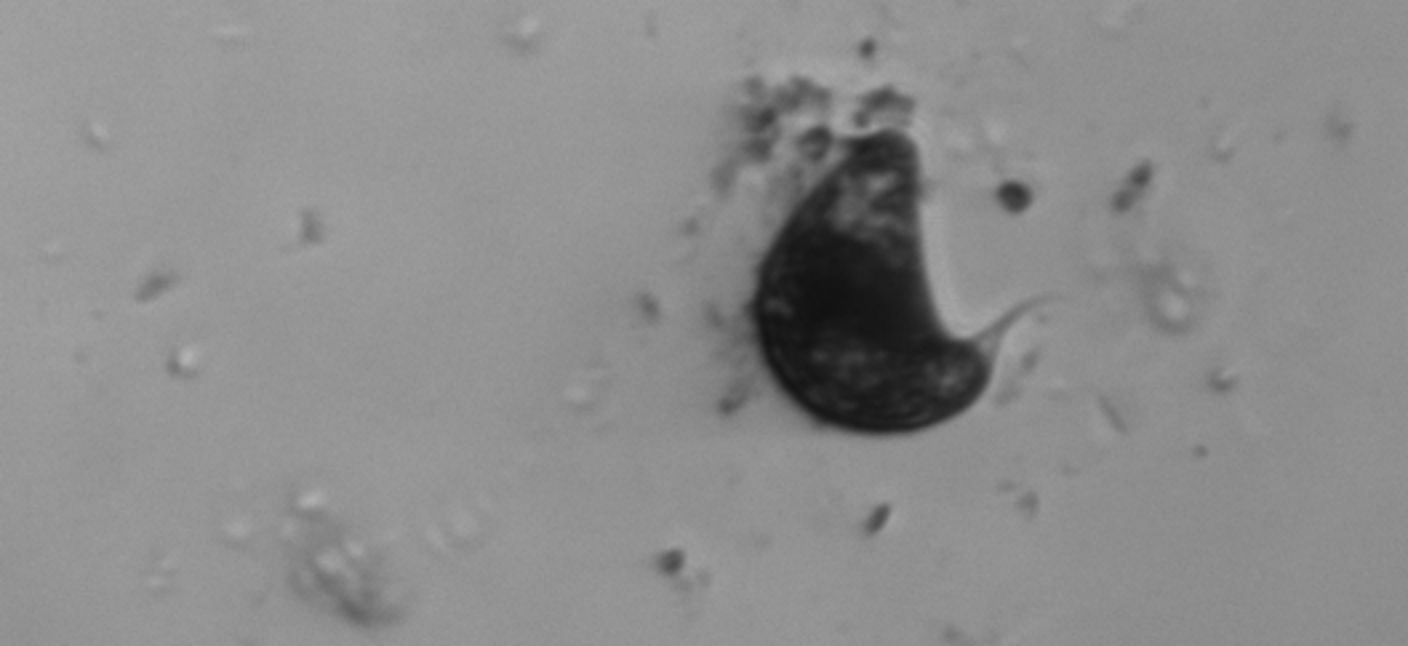

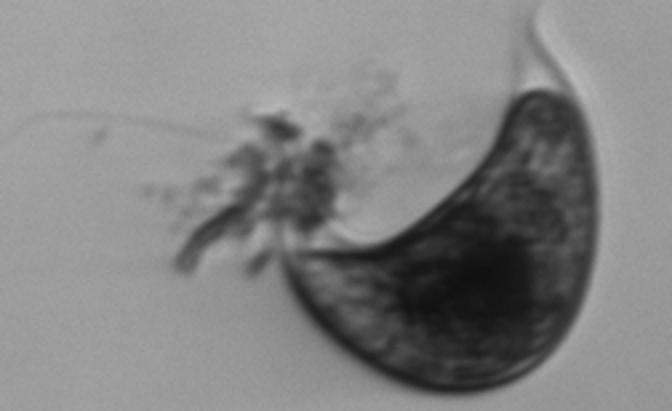

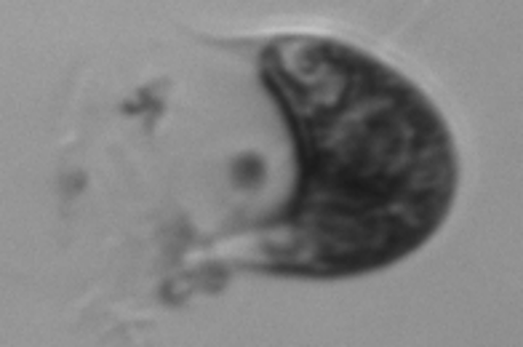

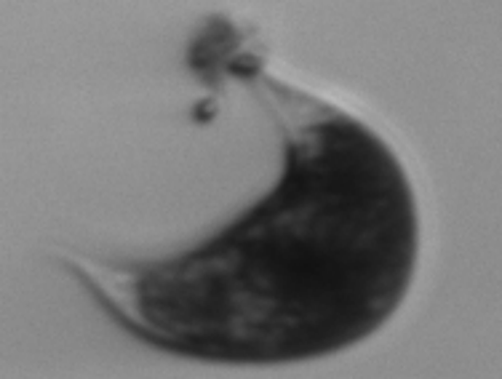

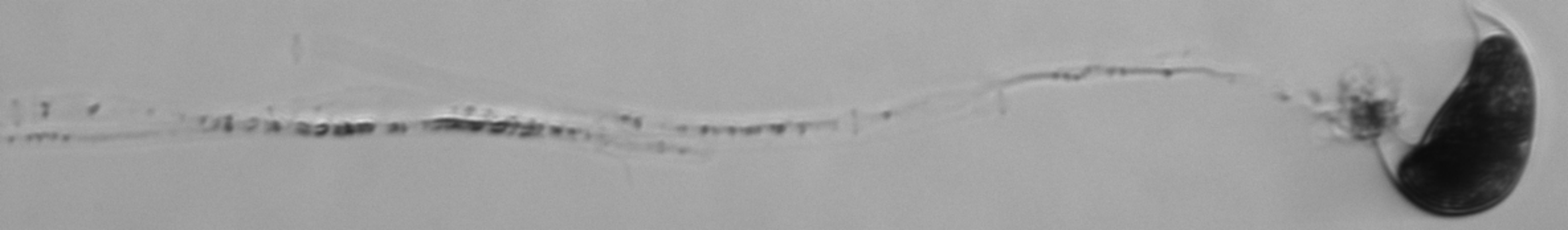

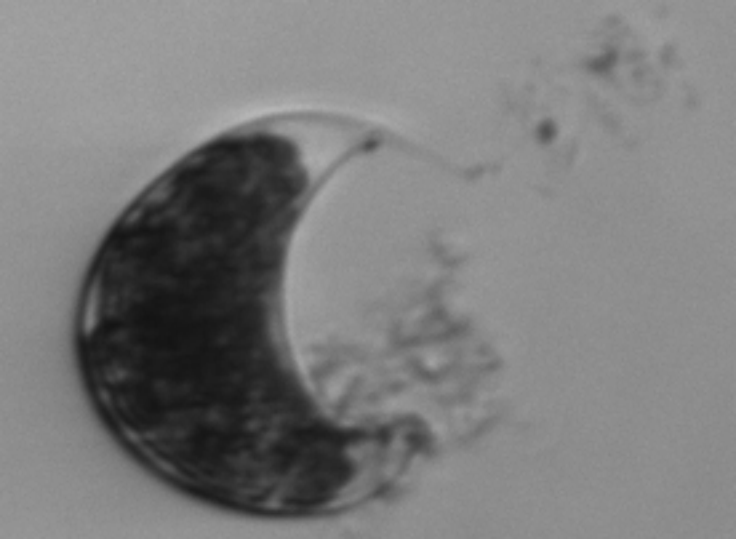

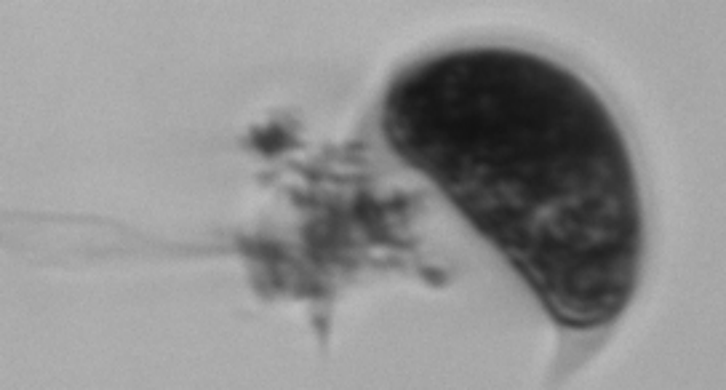

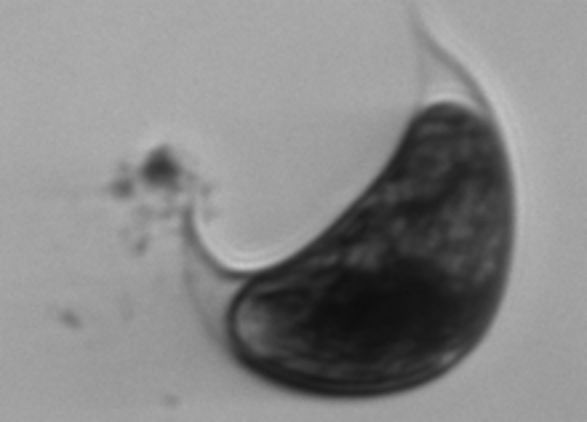

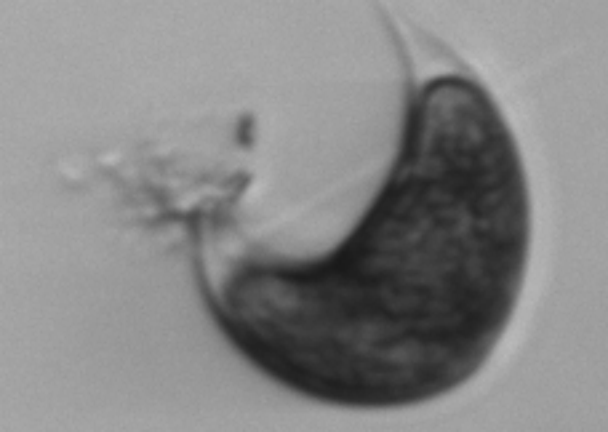

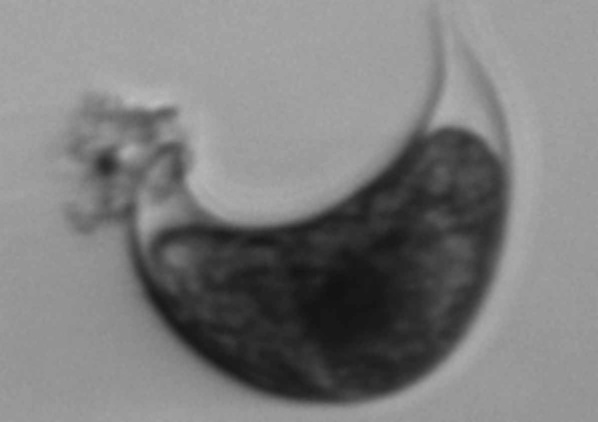

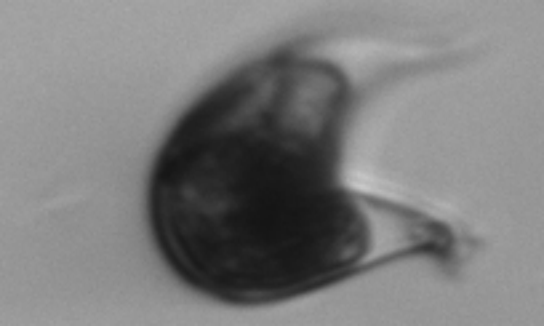

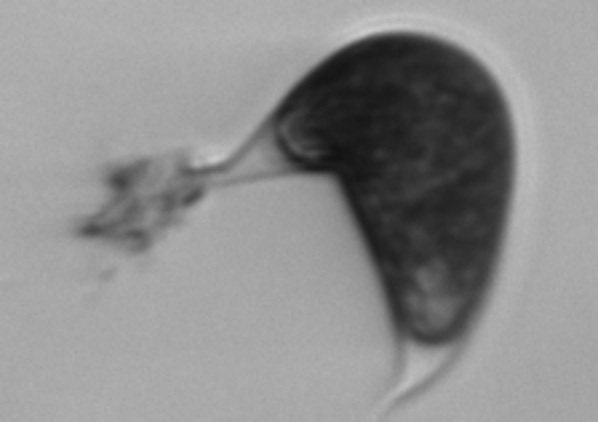

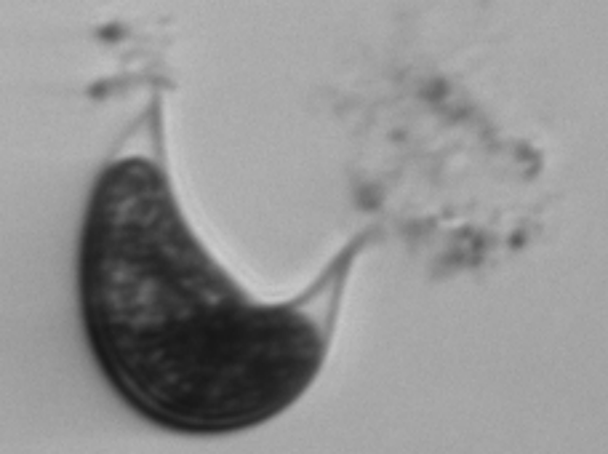

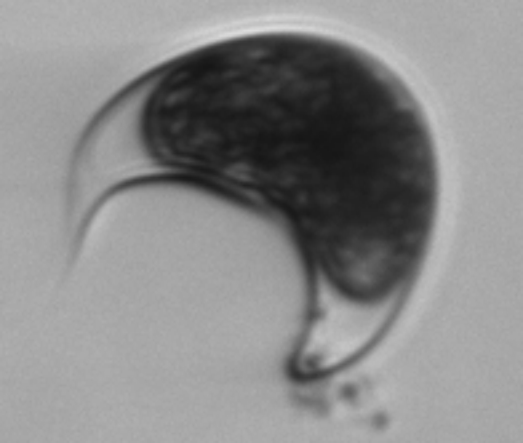

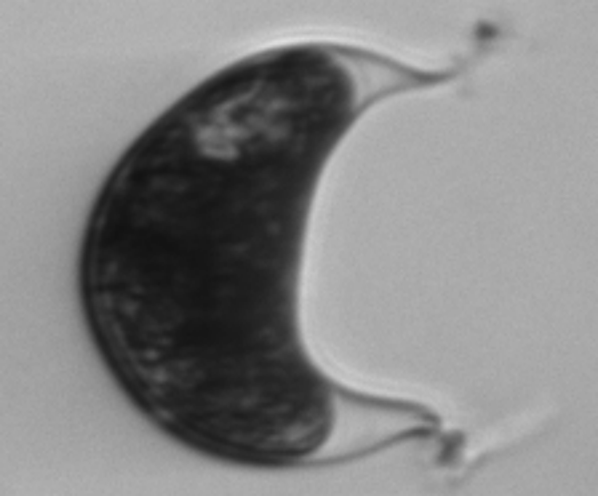

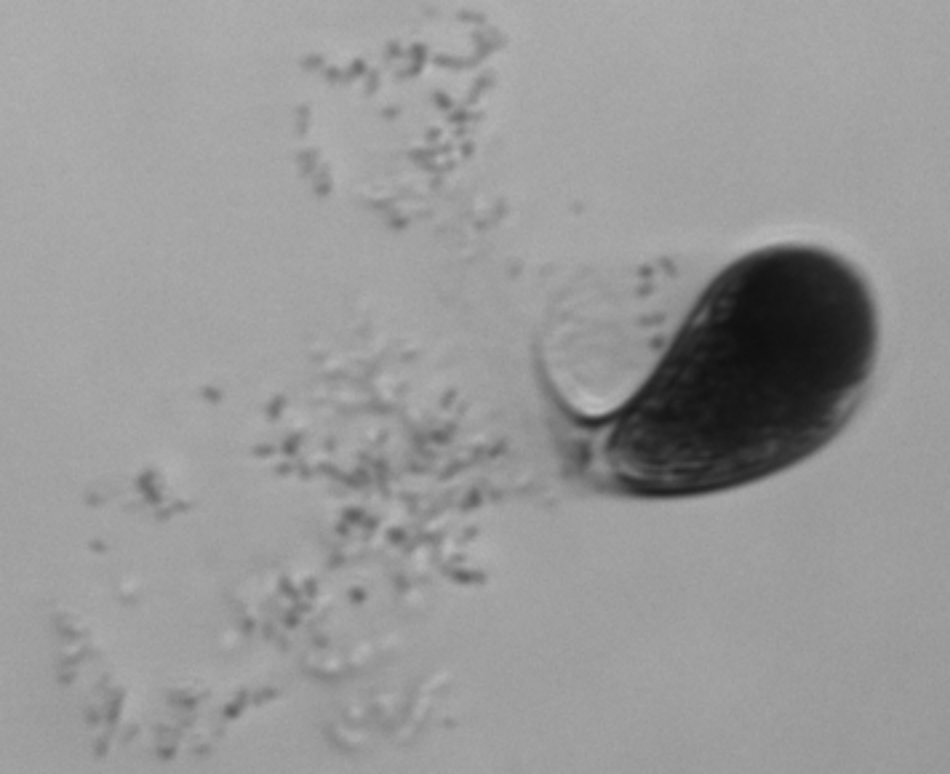

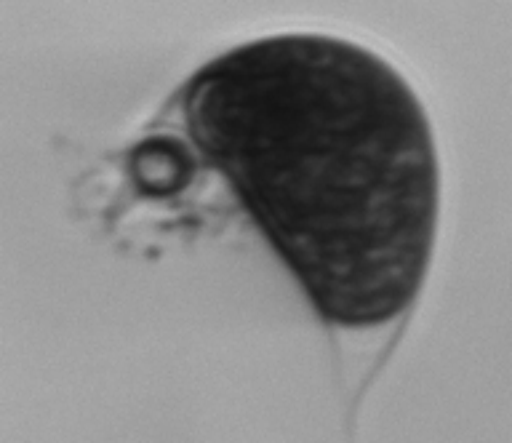

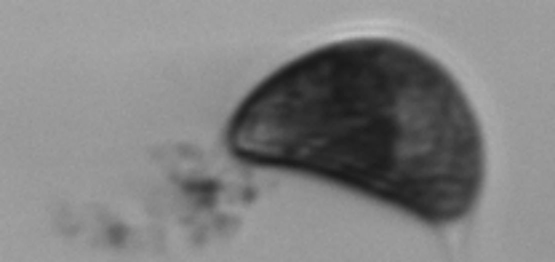

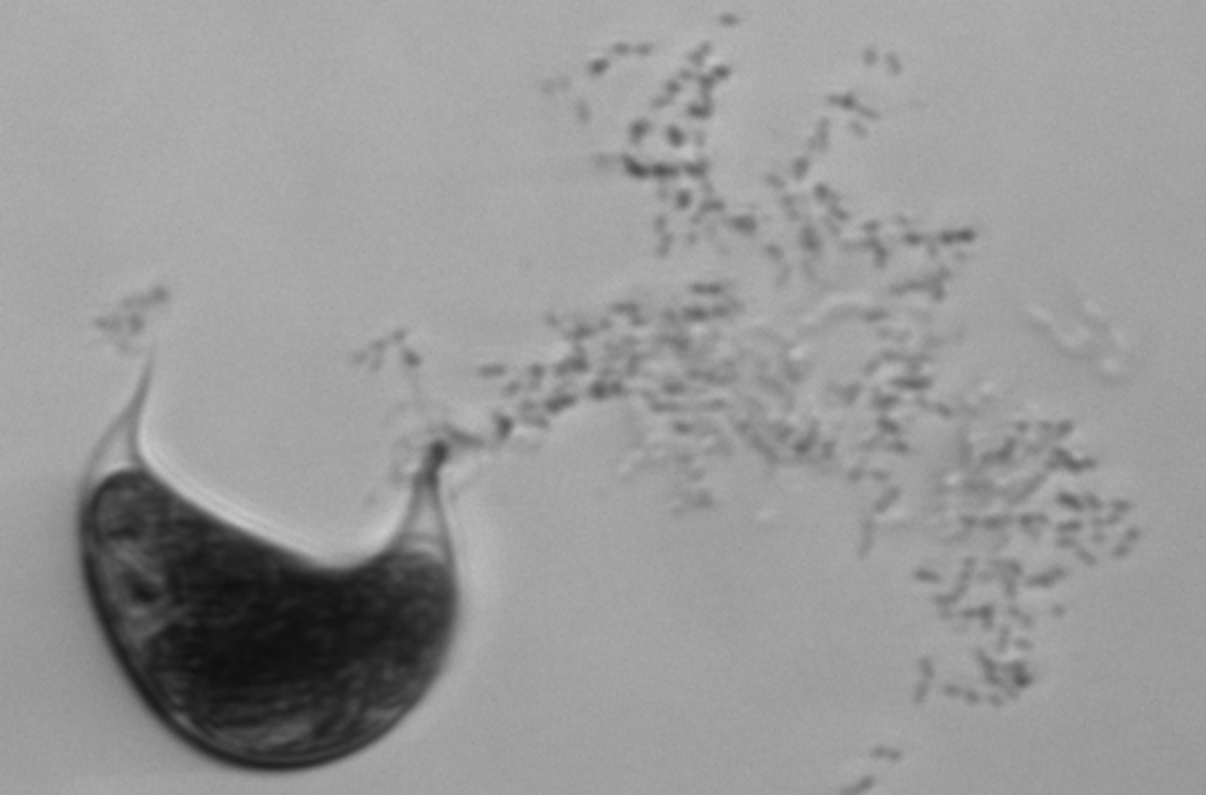

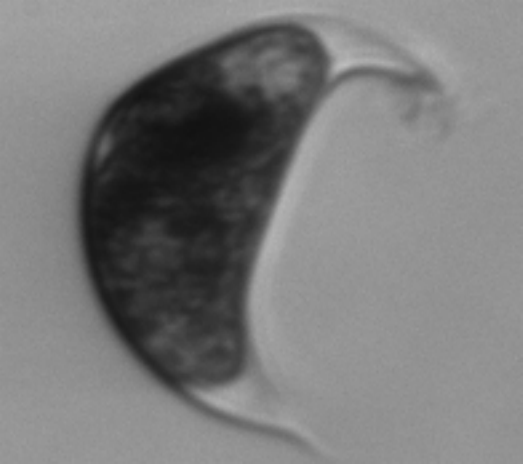

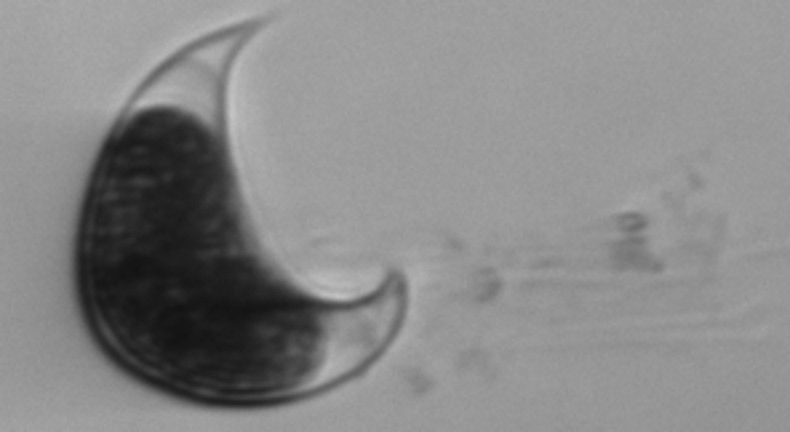

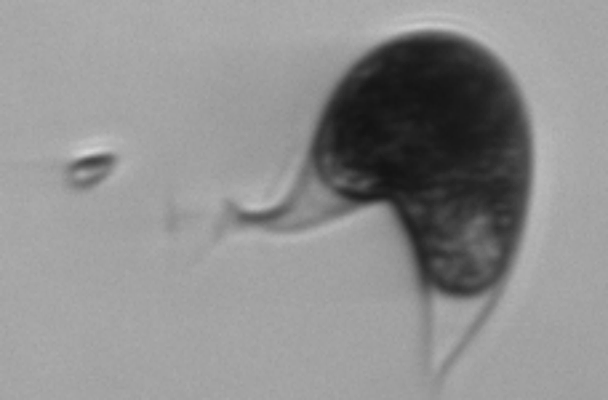

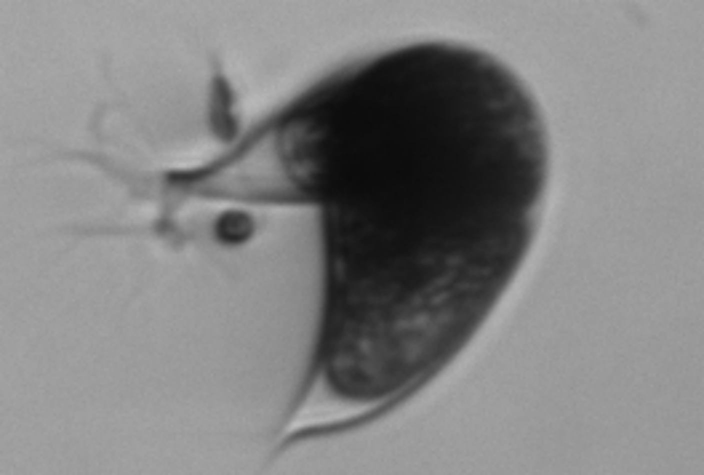

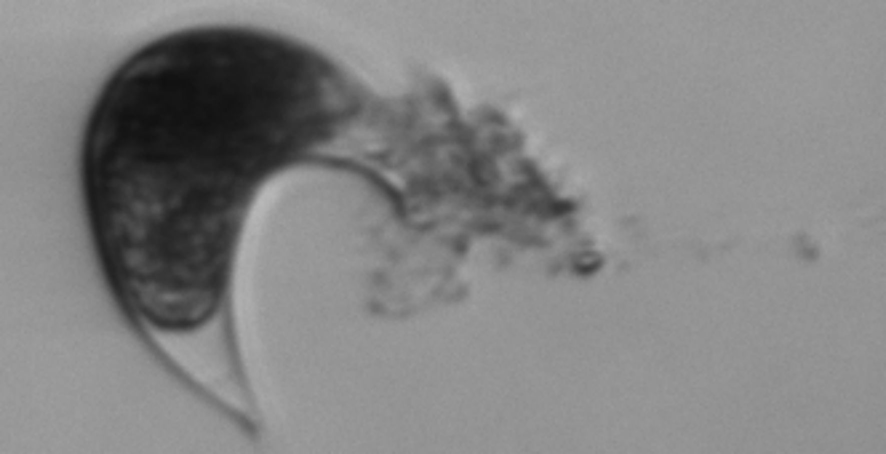

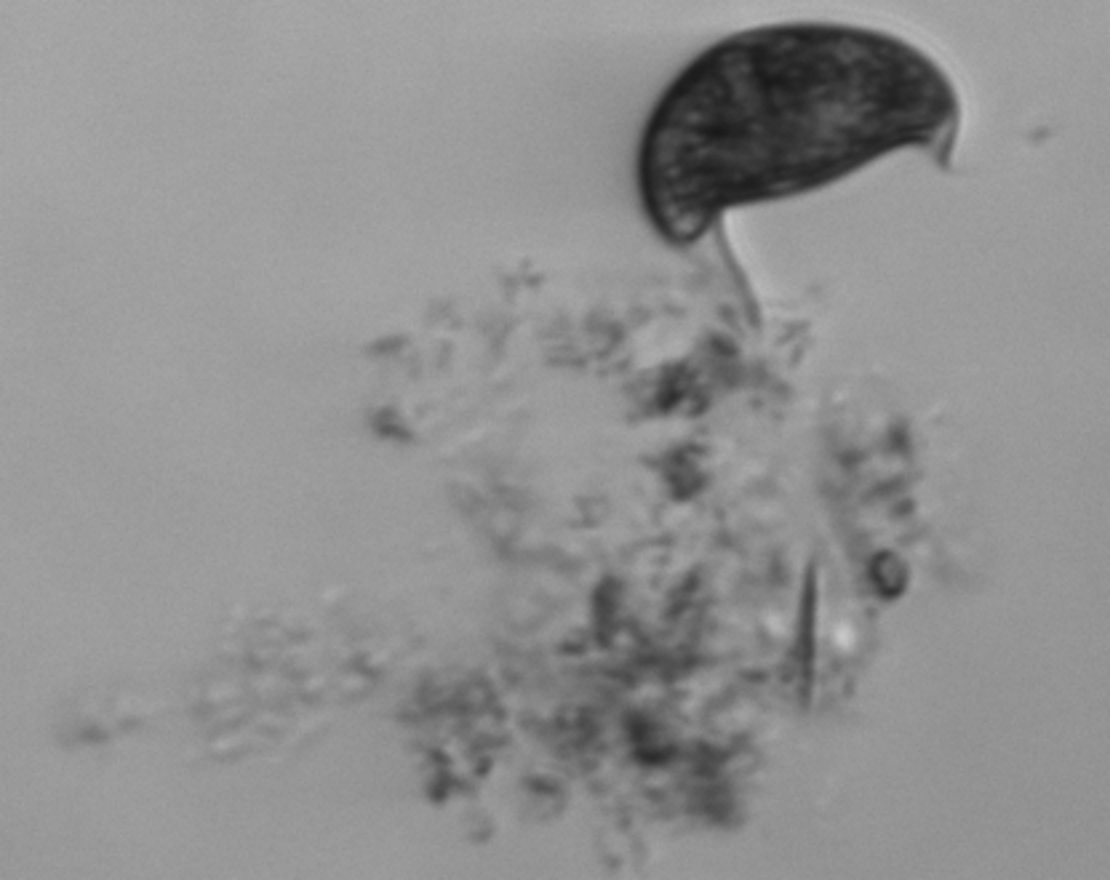

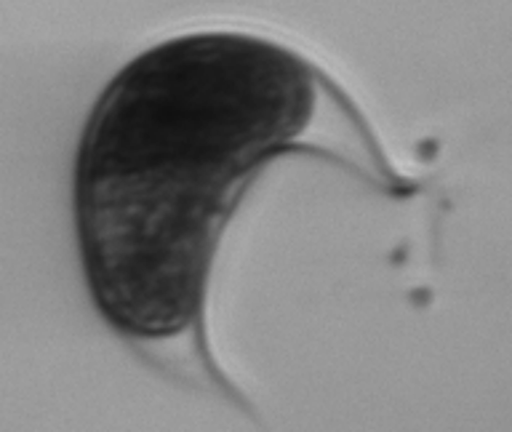

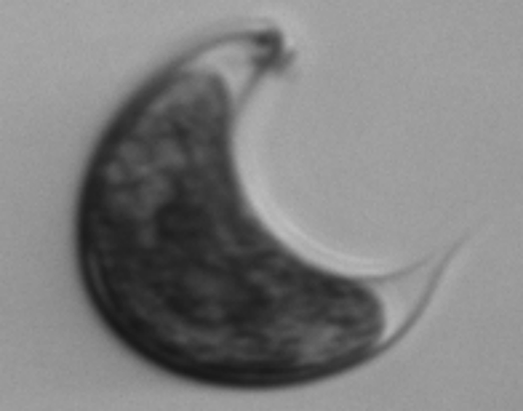

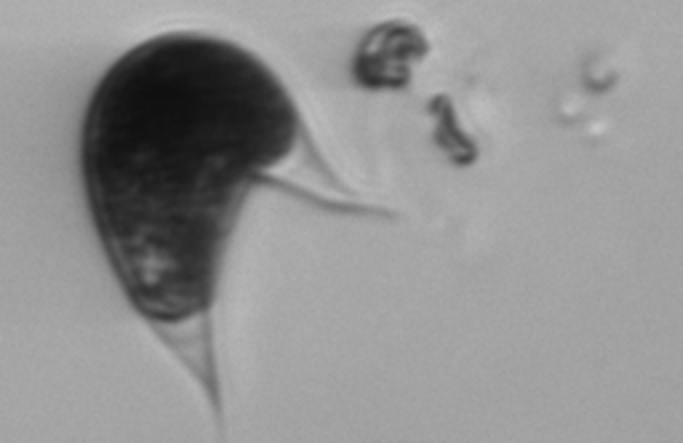

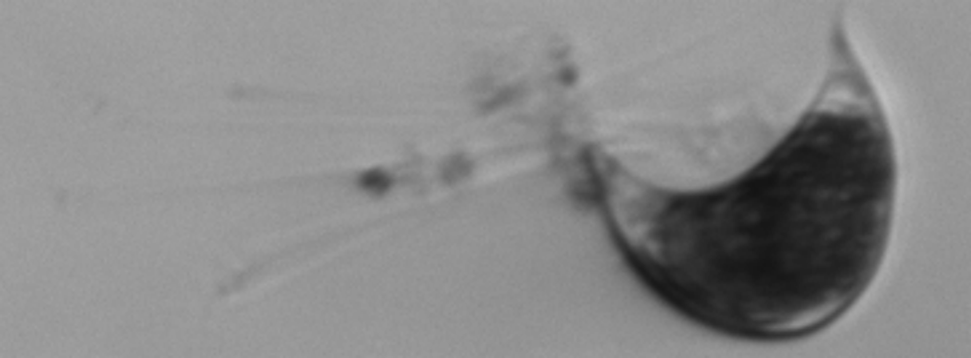

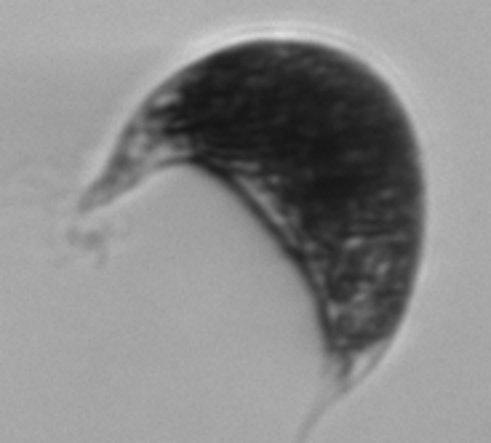

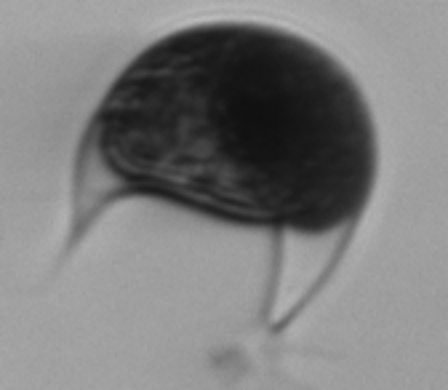

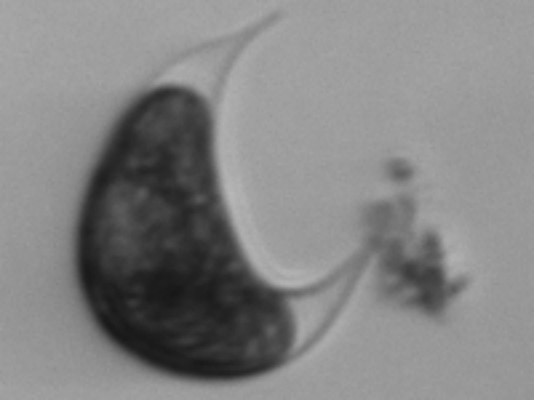

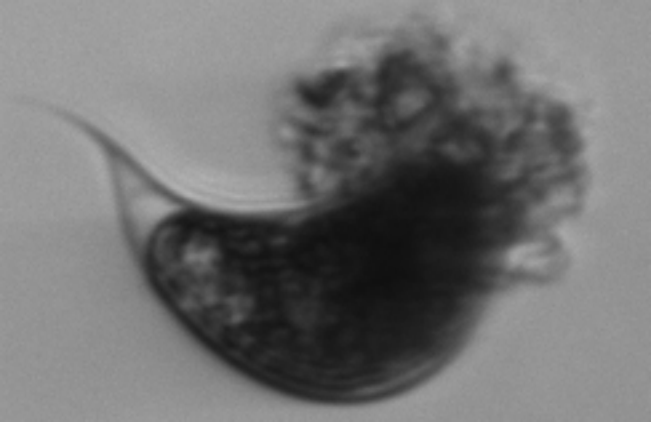

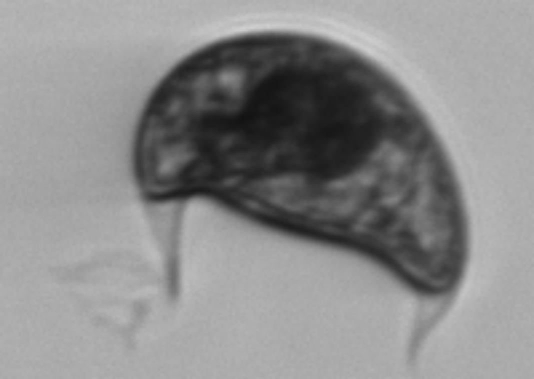

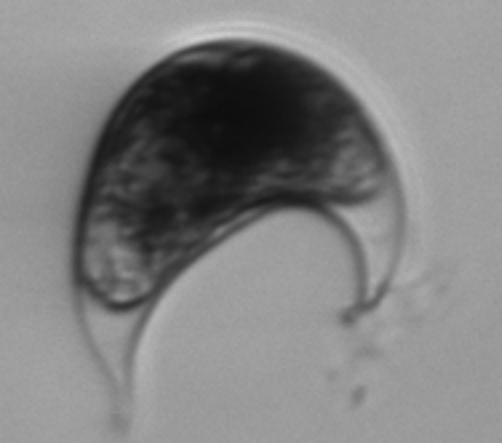

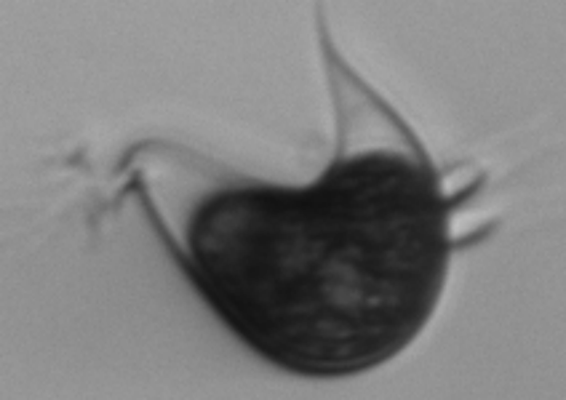

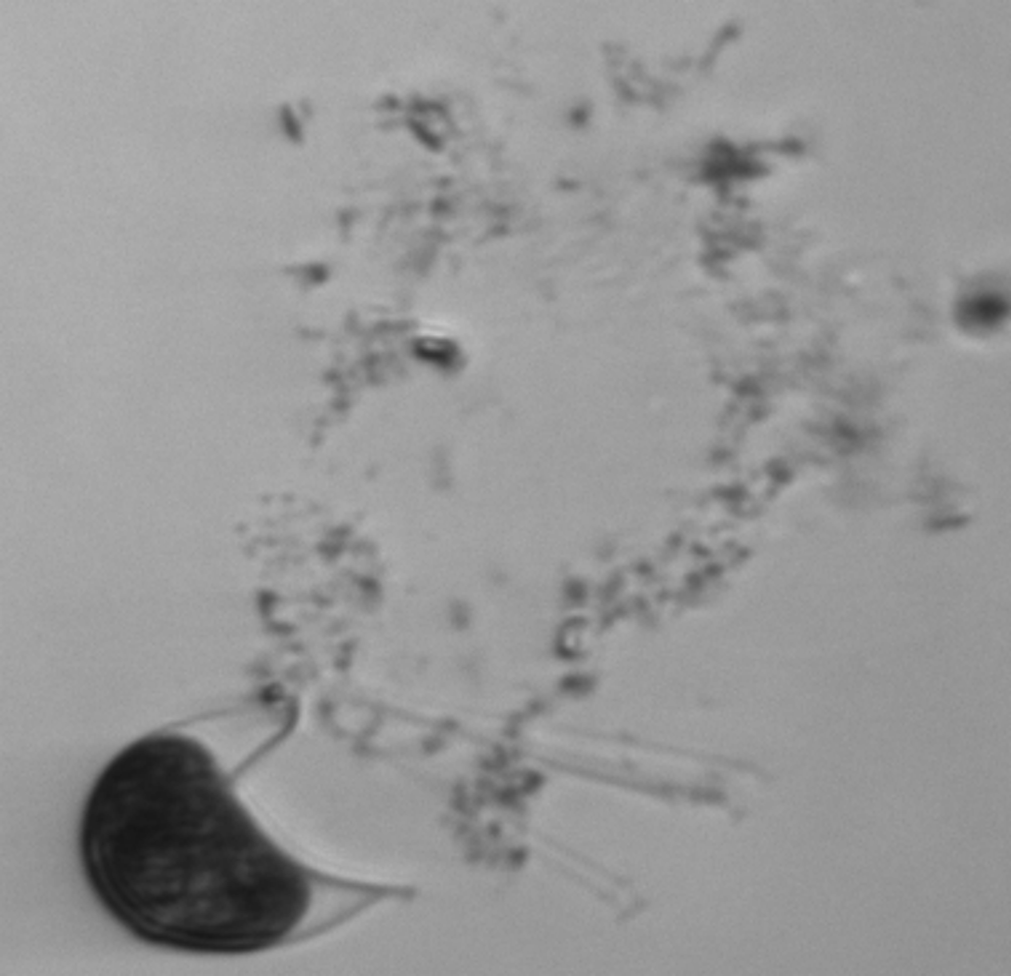

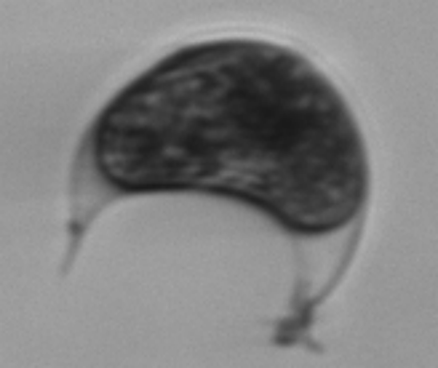

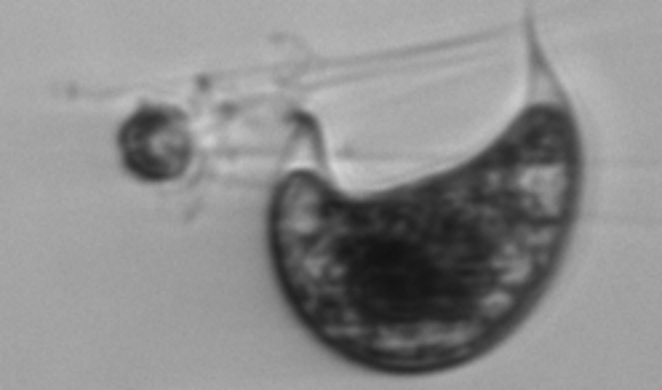

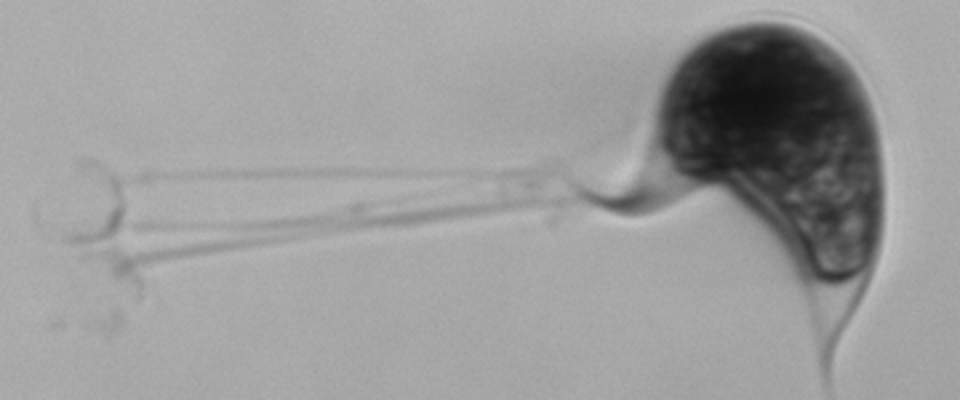

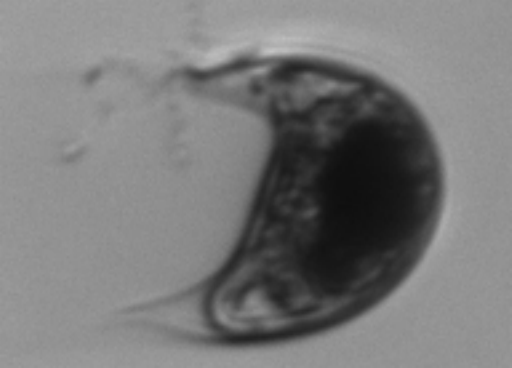

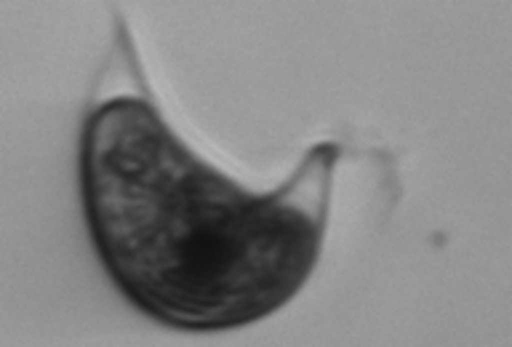

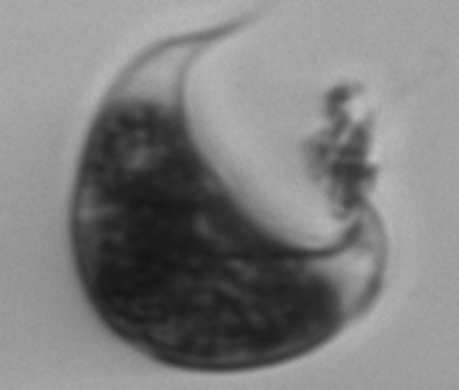

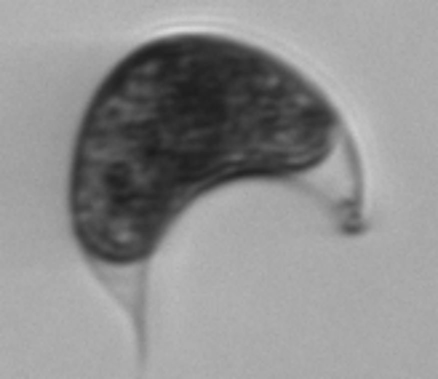

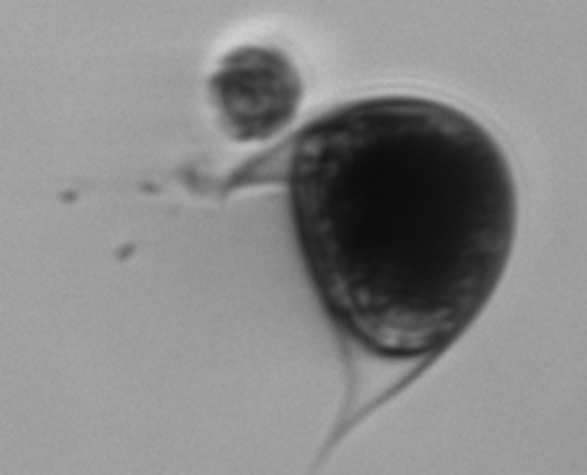

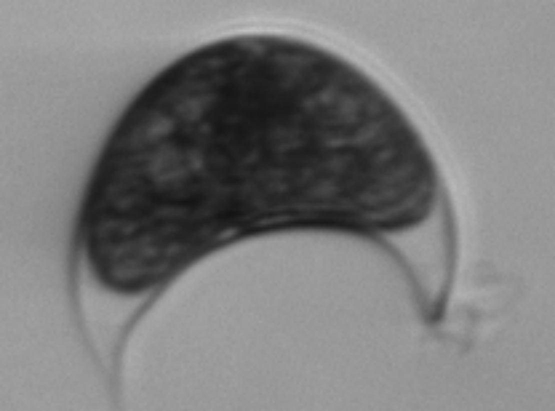

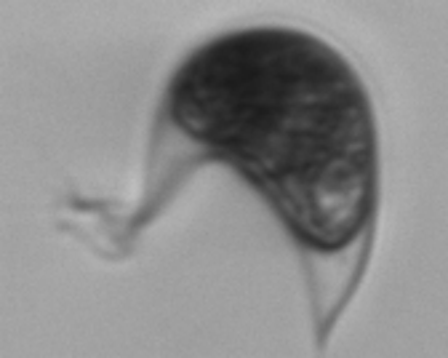

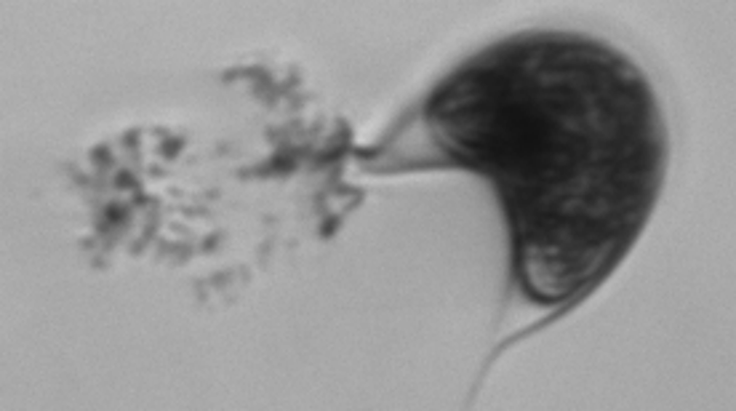

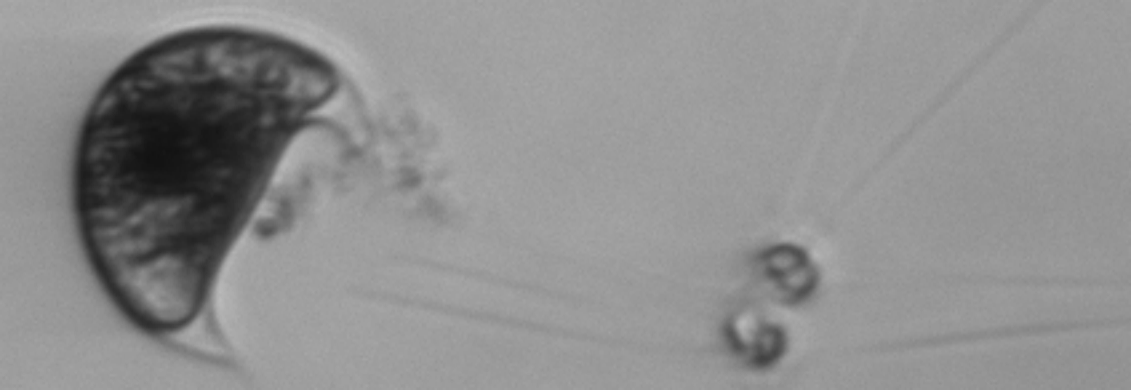

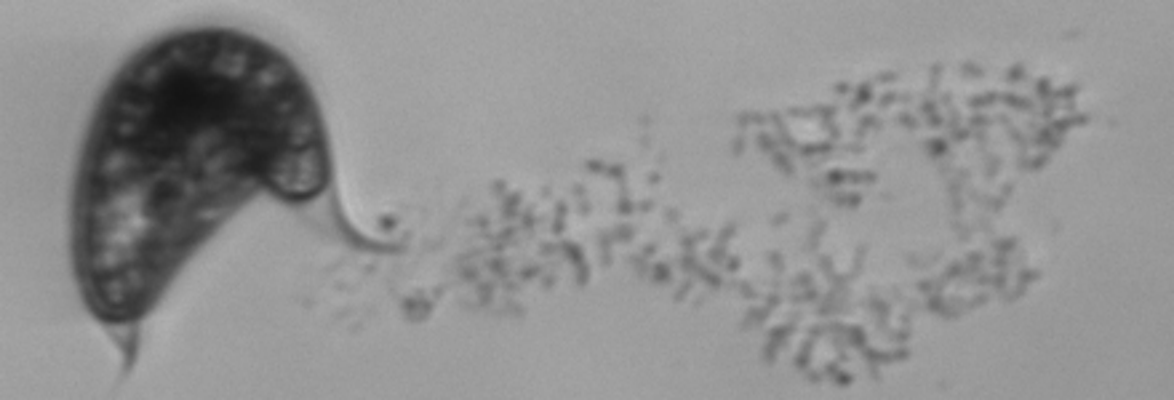

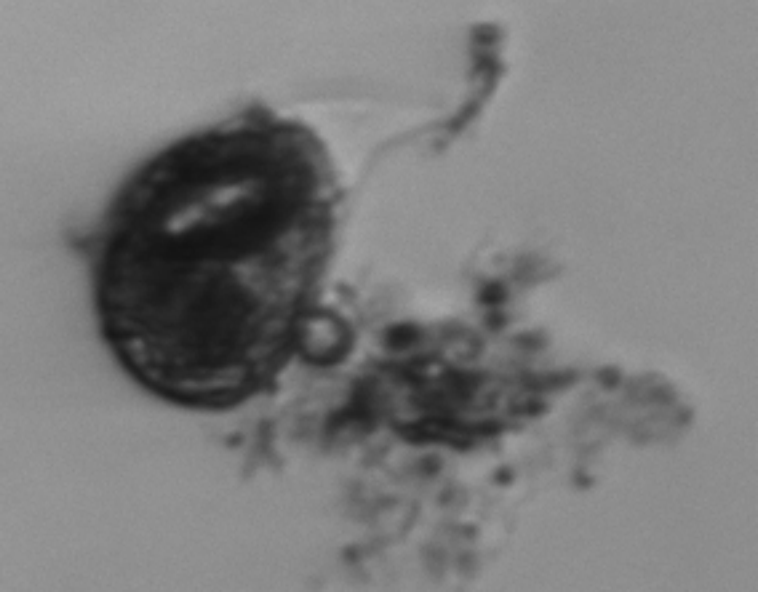

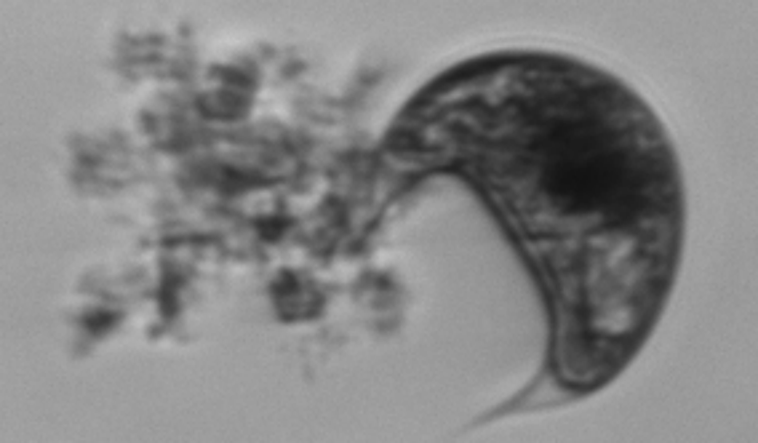

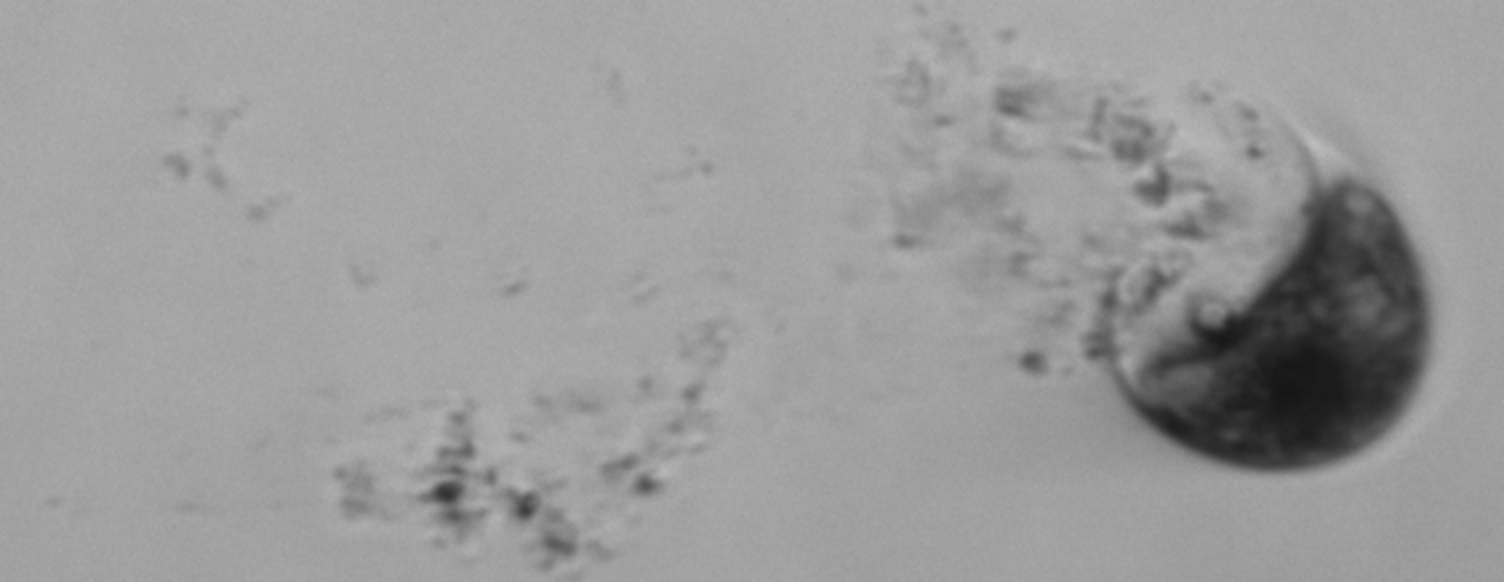

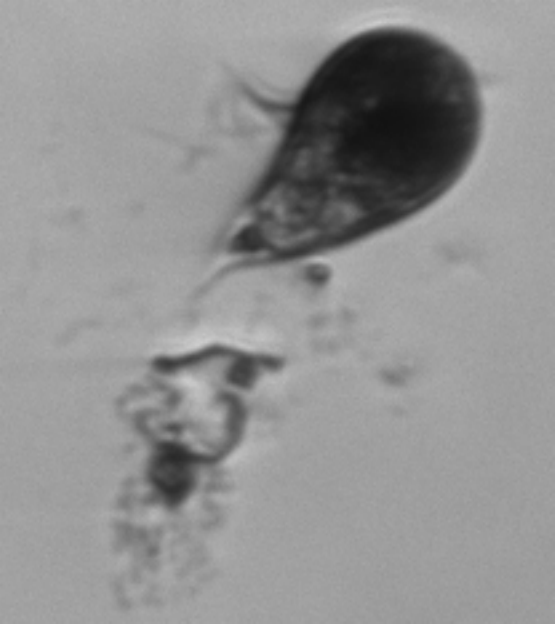

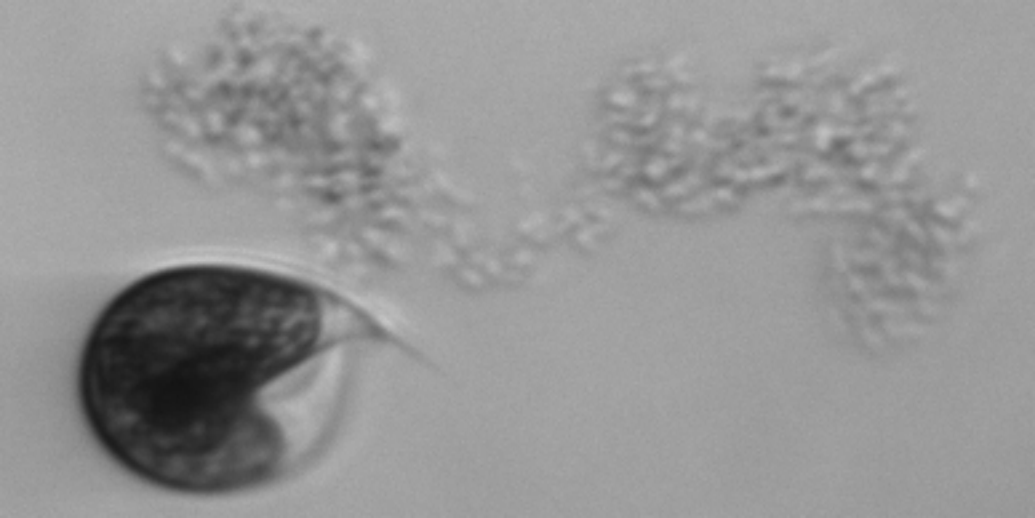

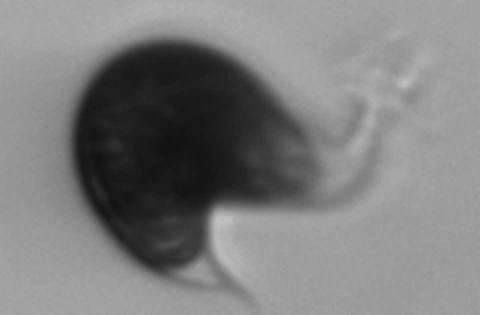

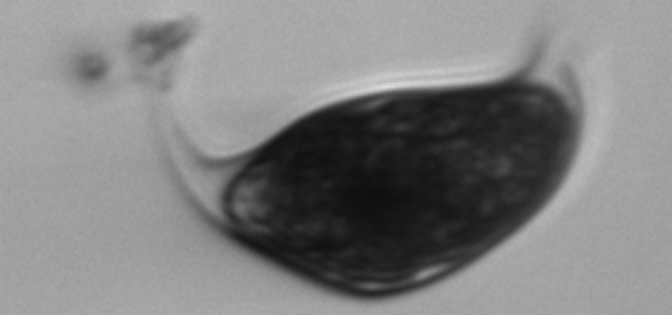

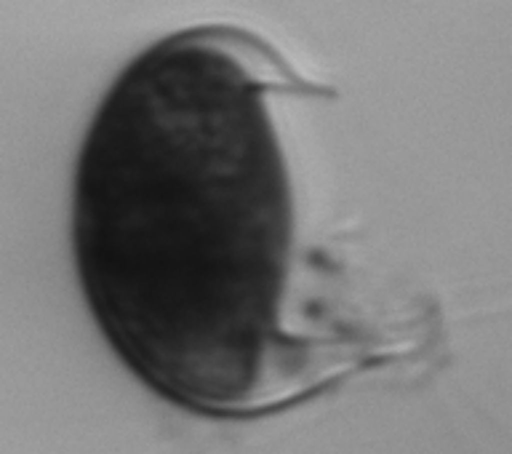

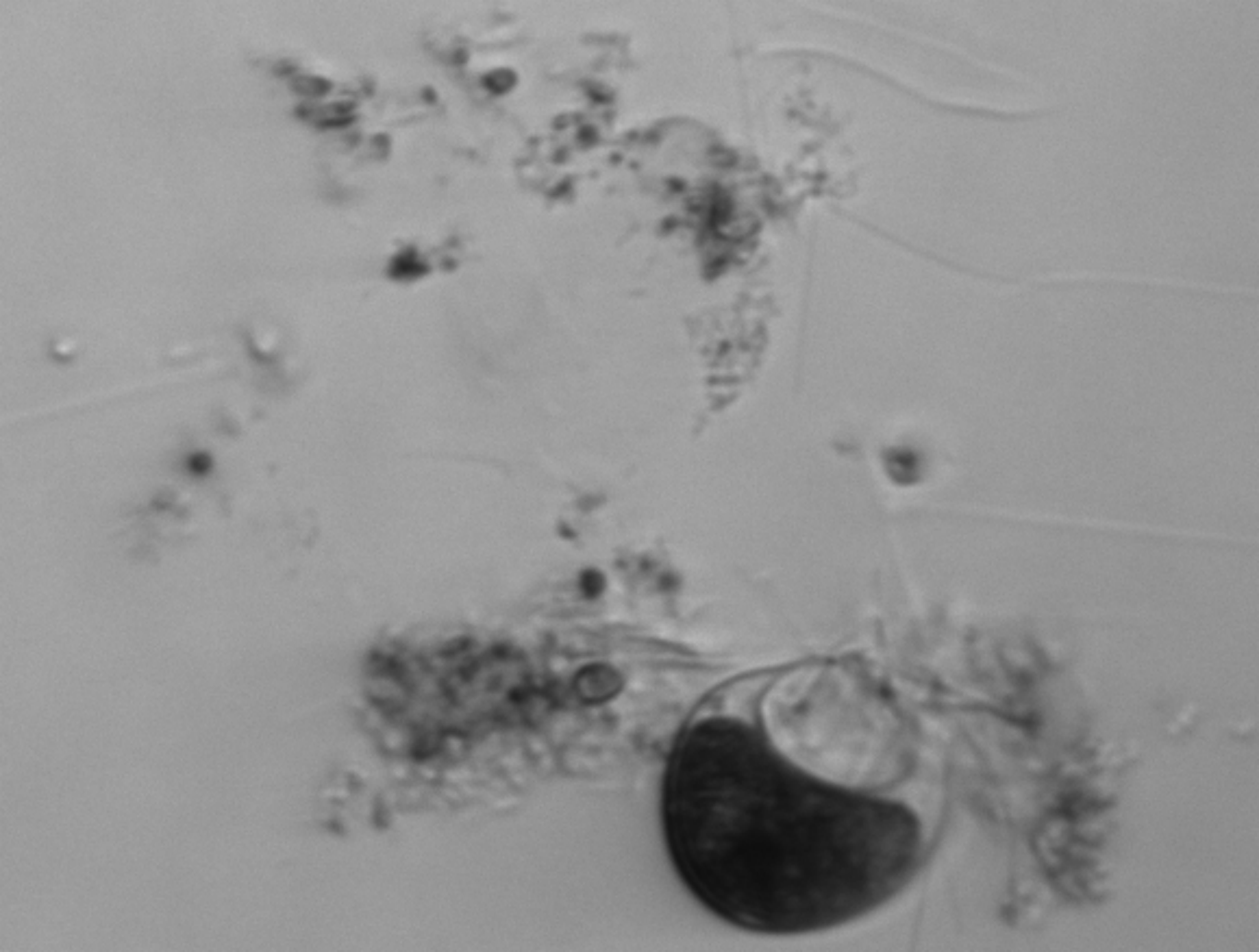

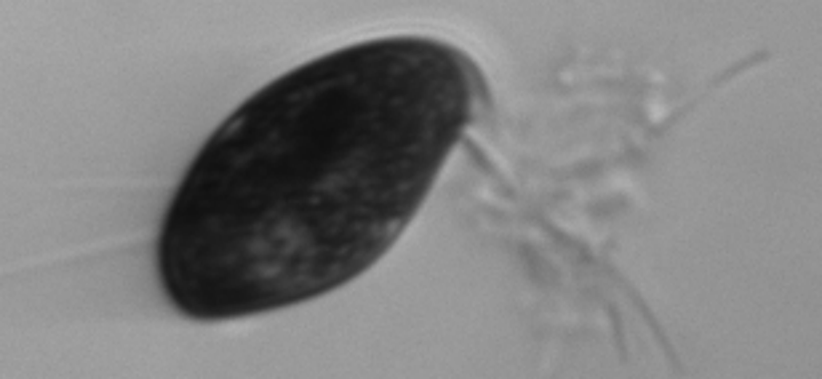

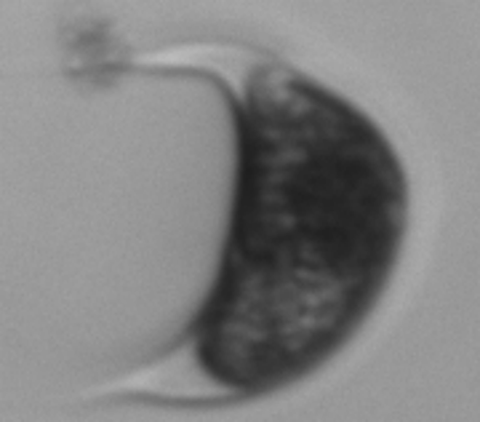

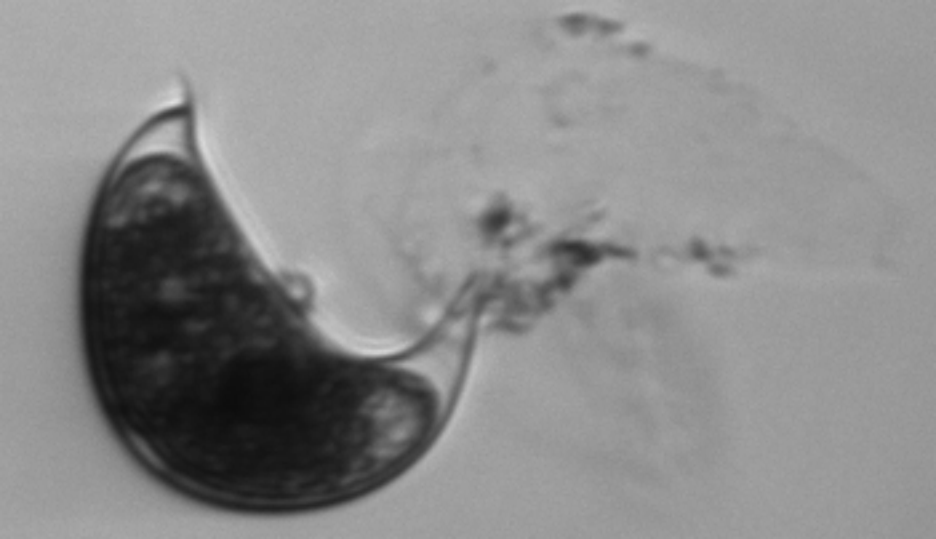

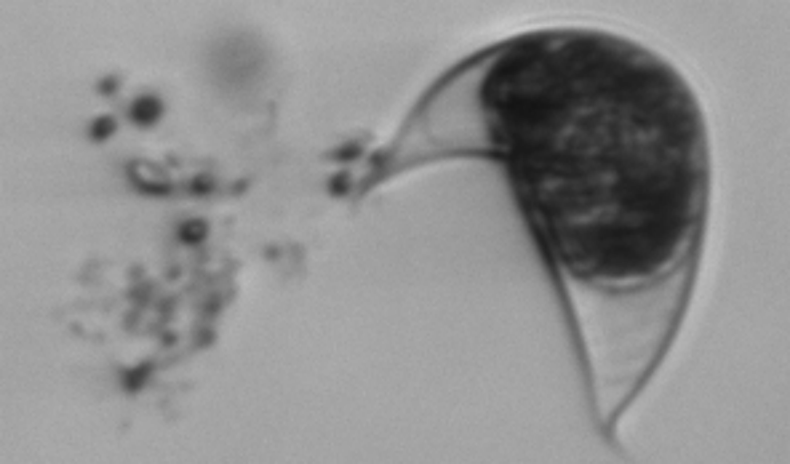

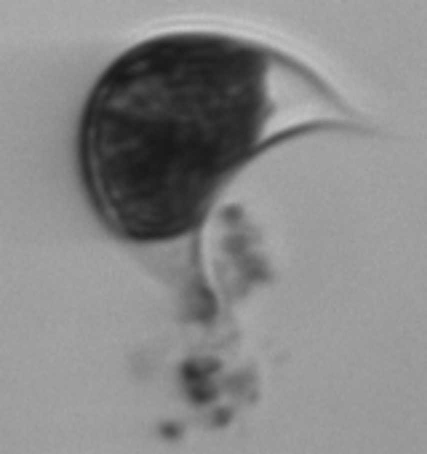

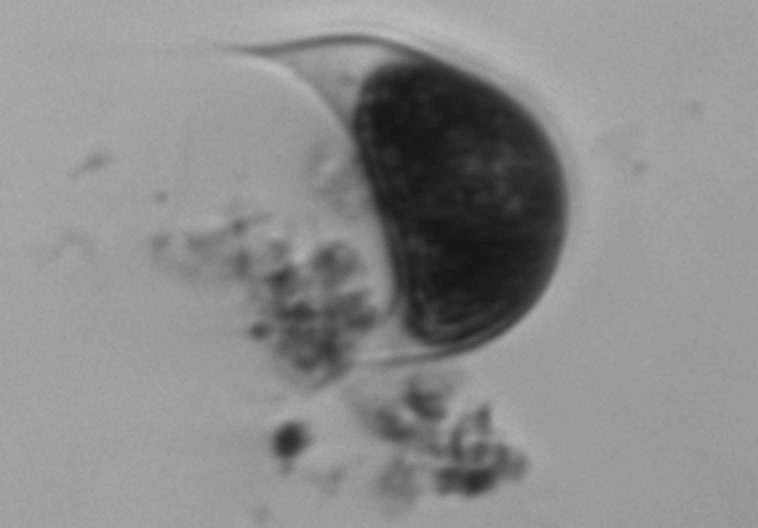

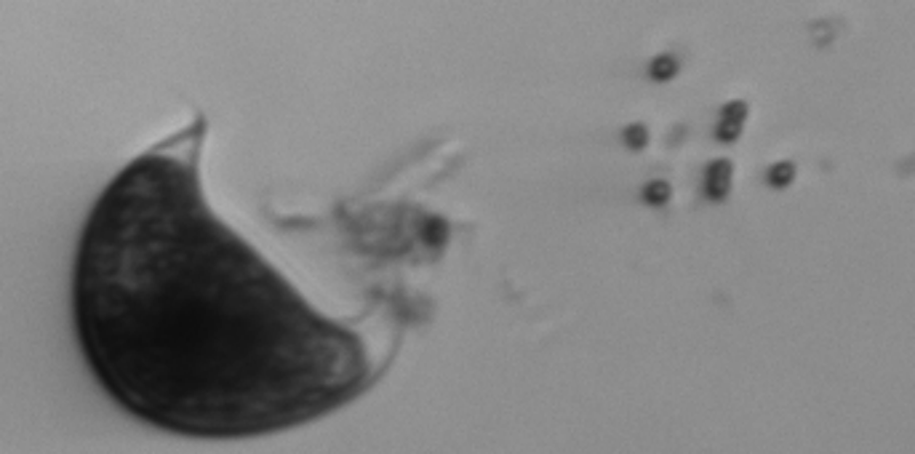

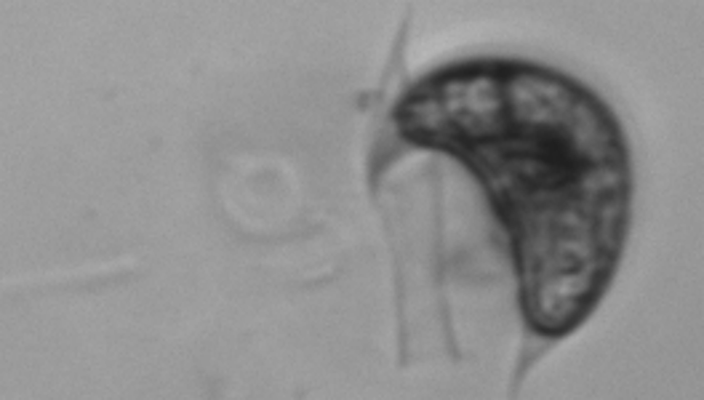

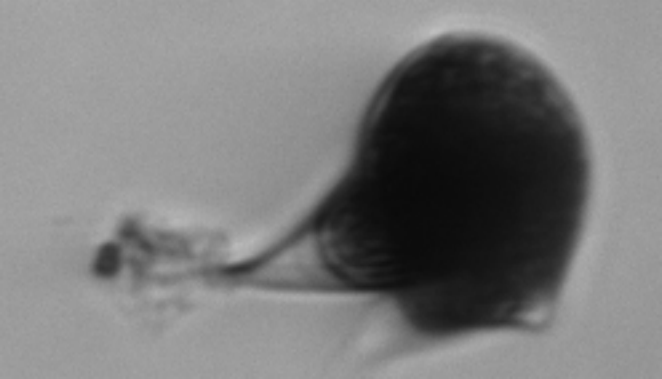

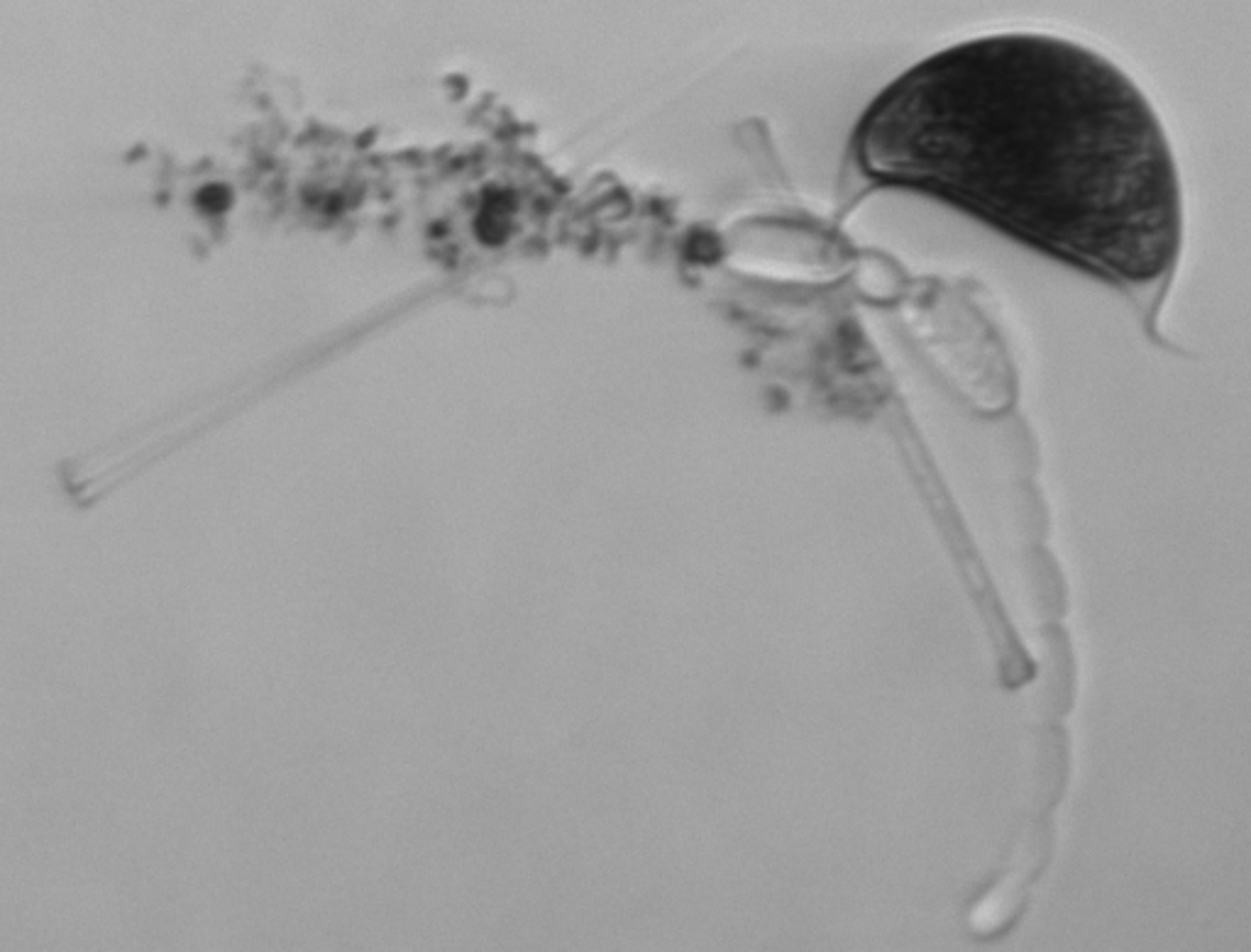

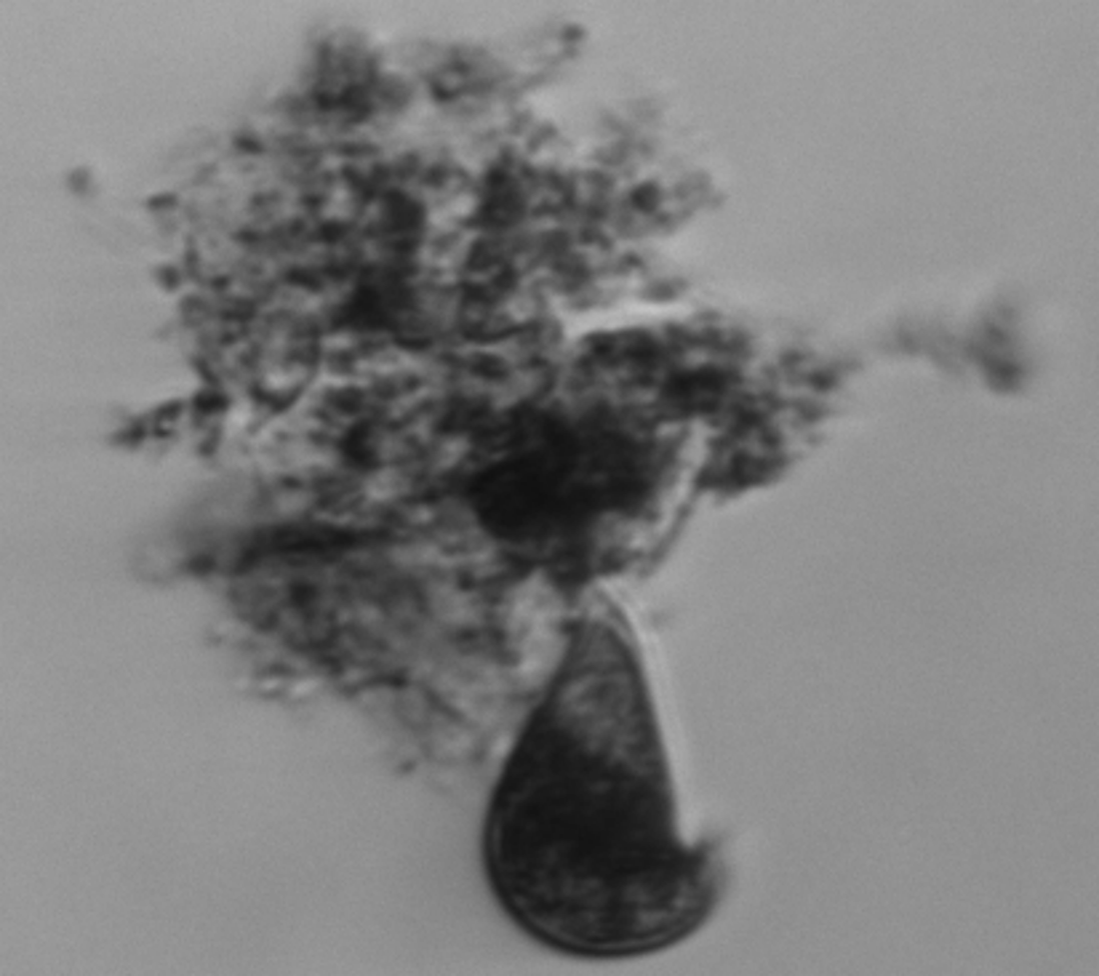

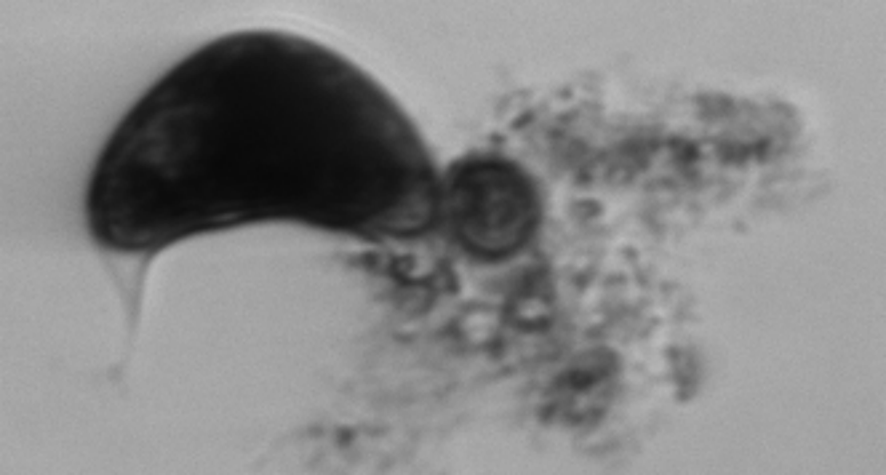

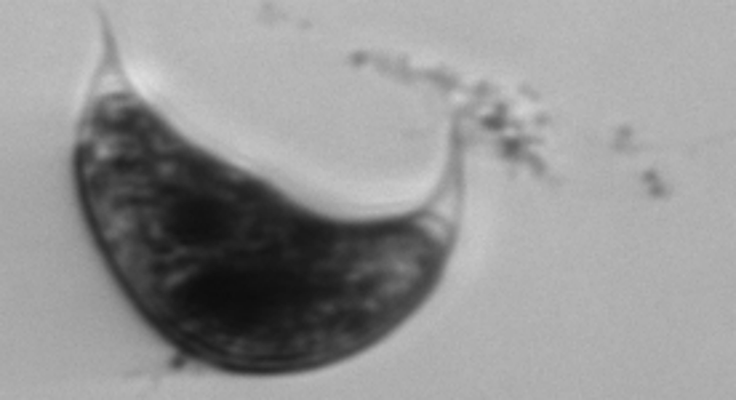

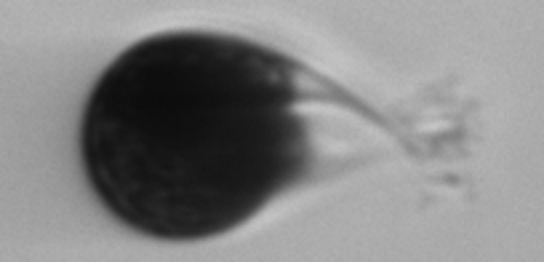

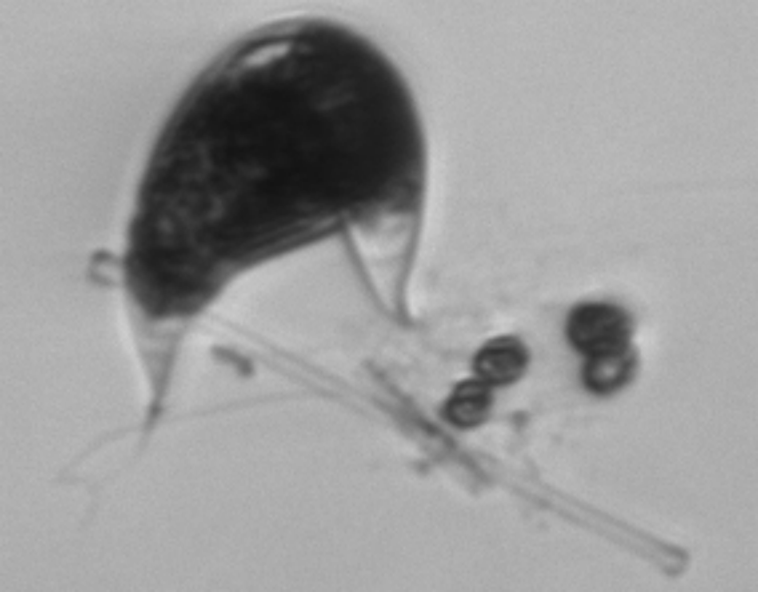

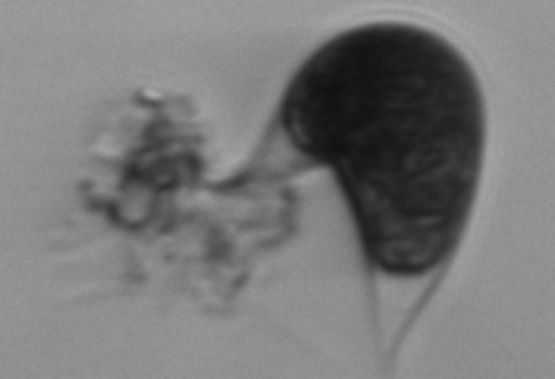

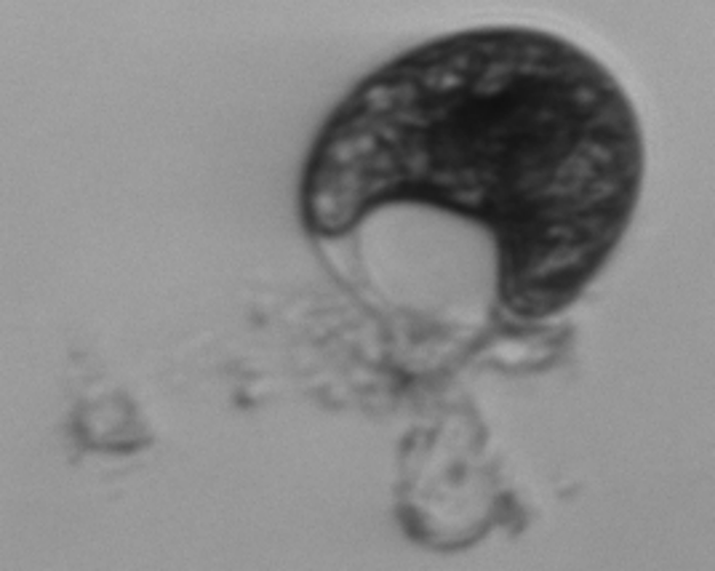

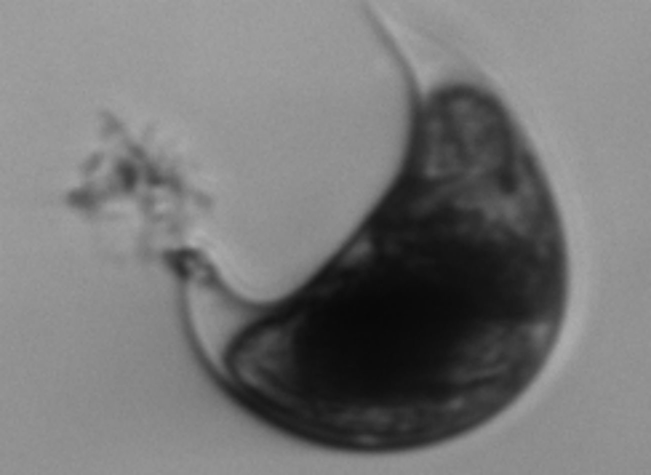

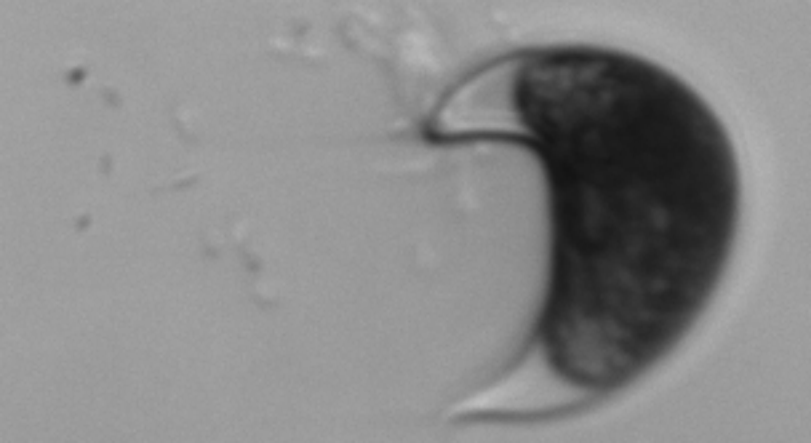

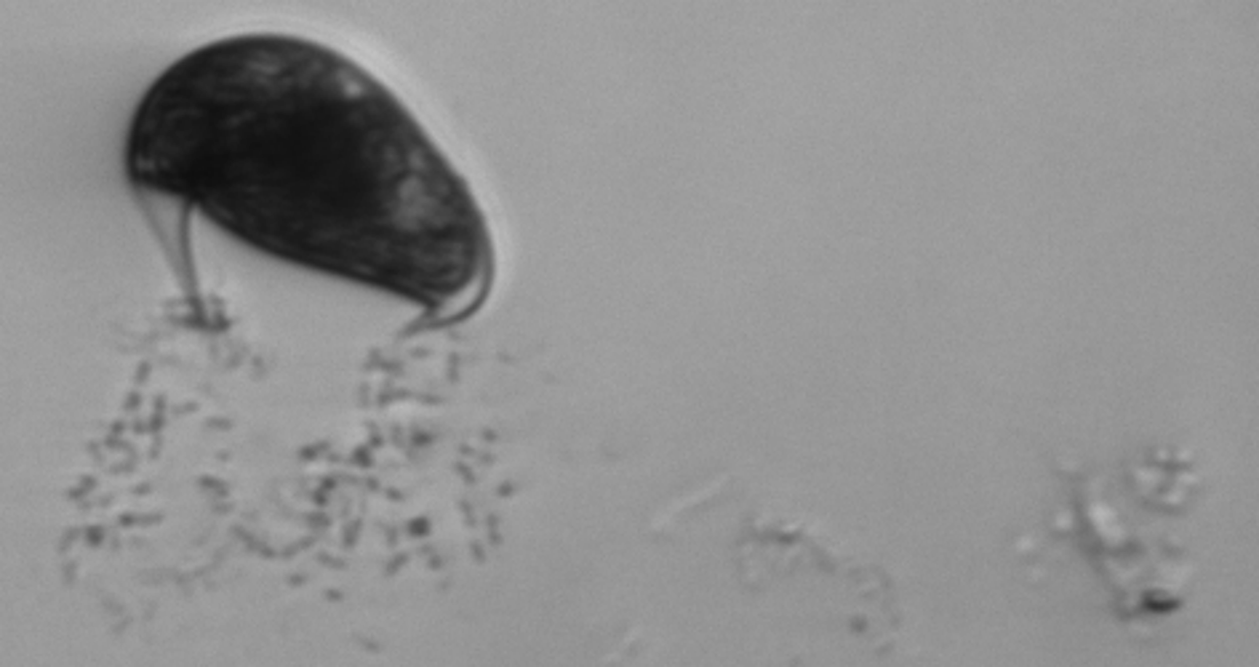

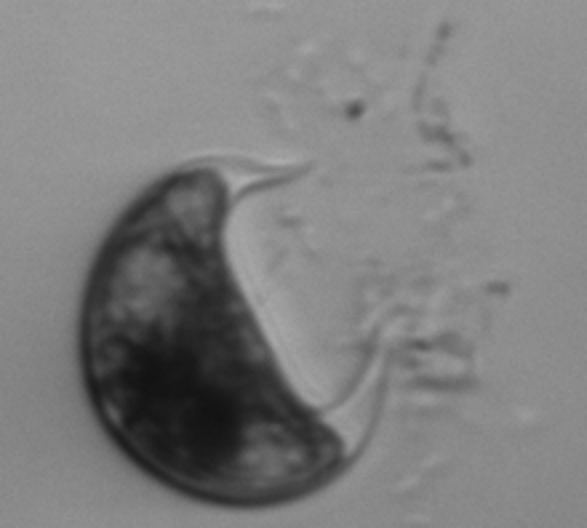

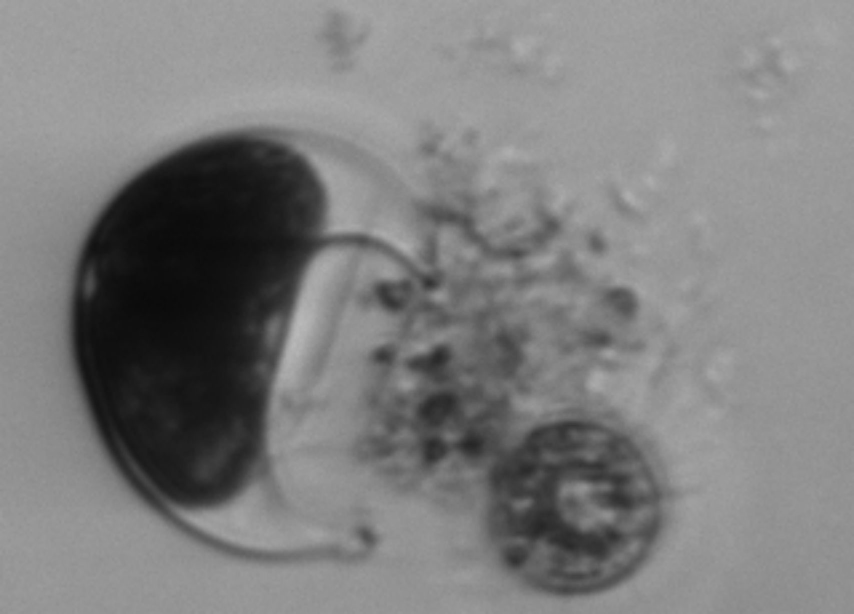

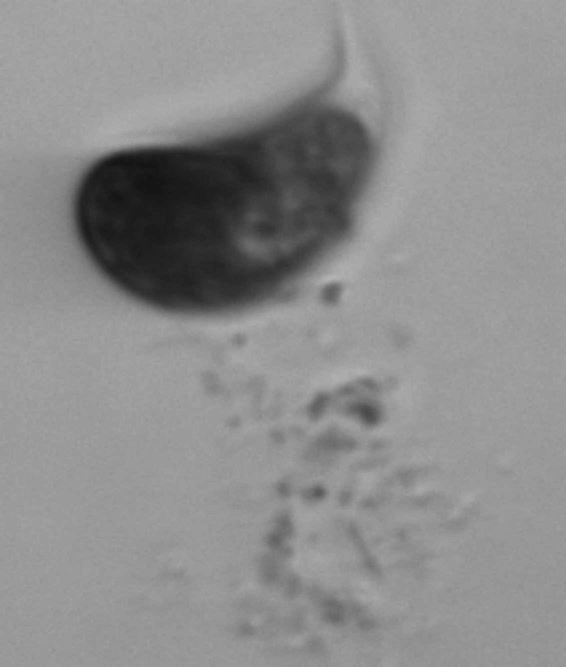

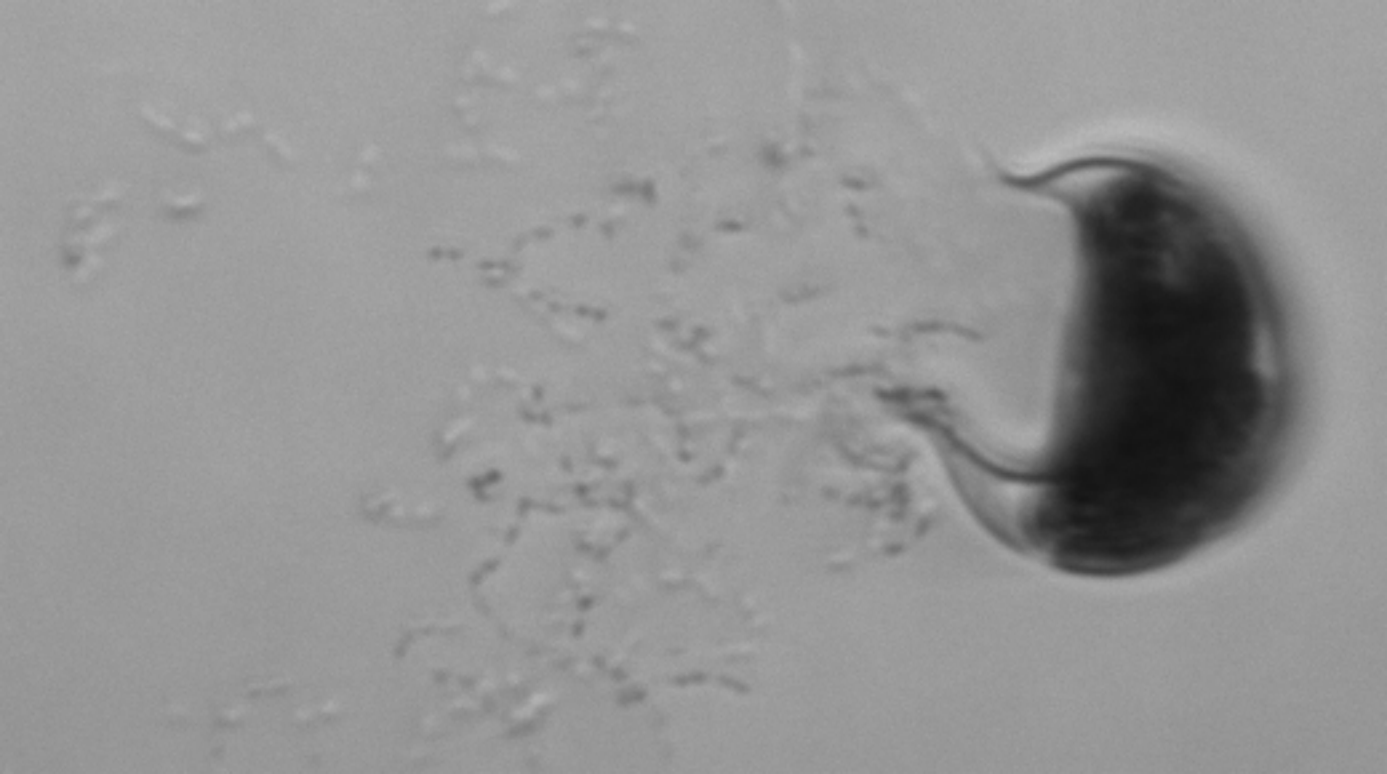

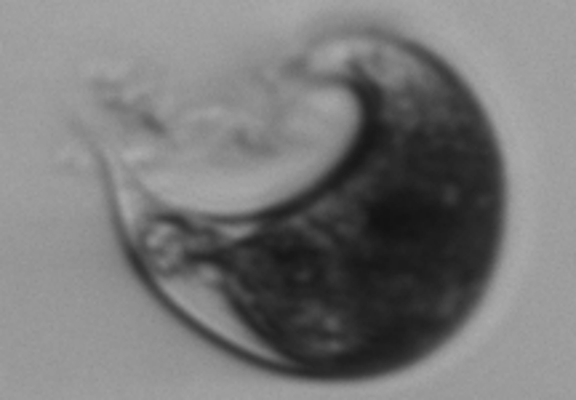

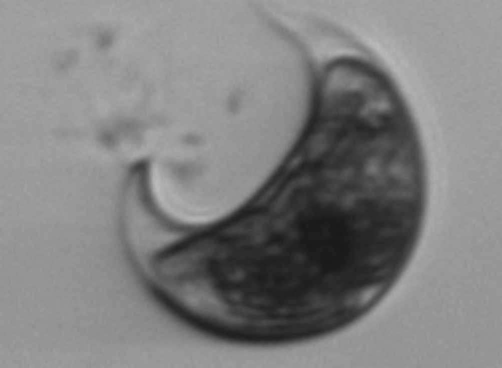

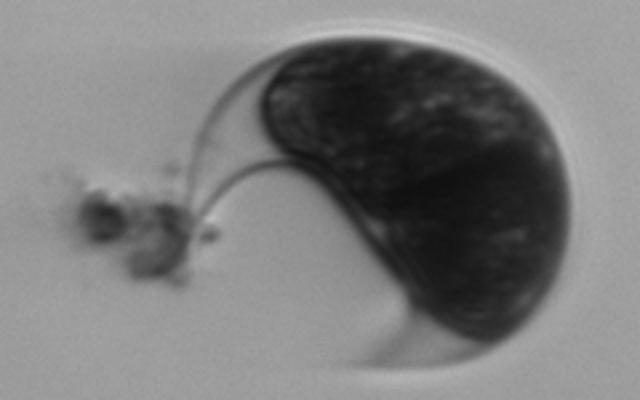

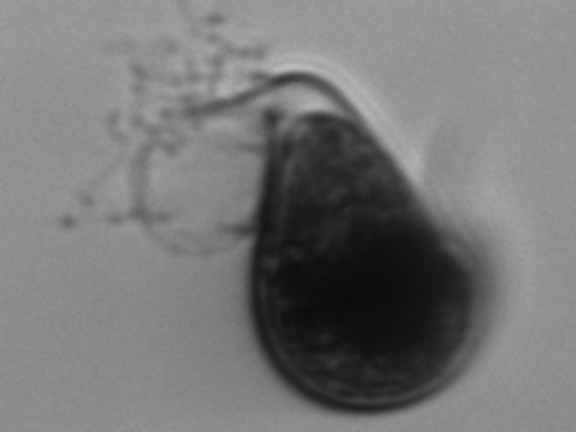

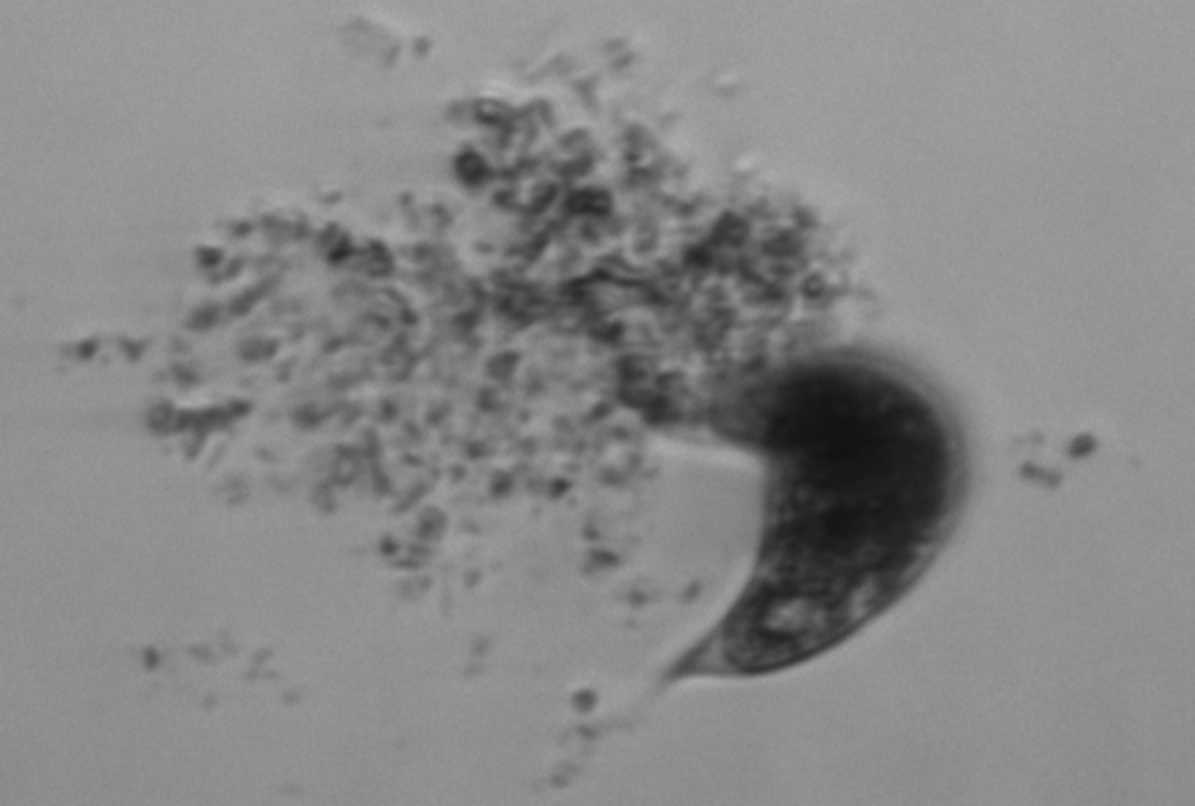

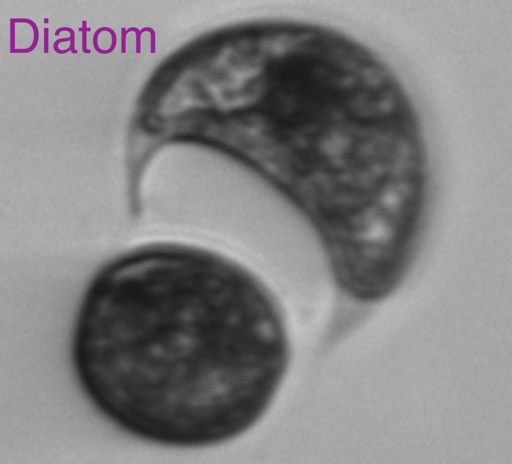
Diatom

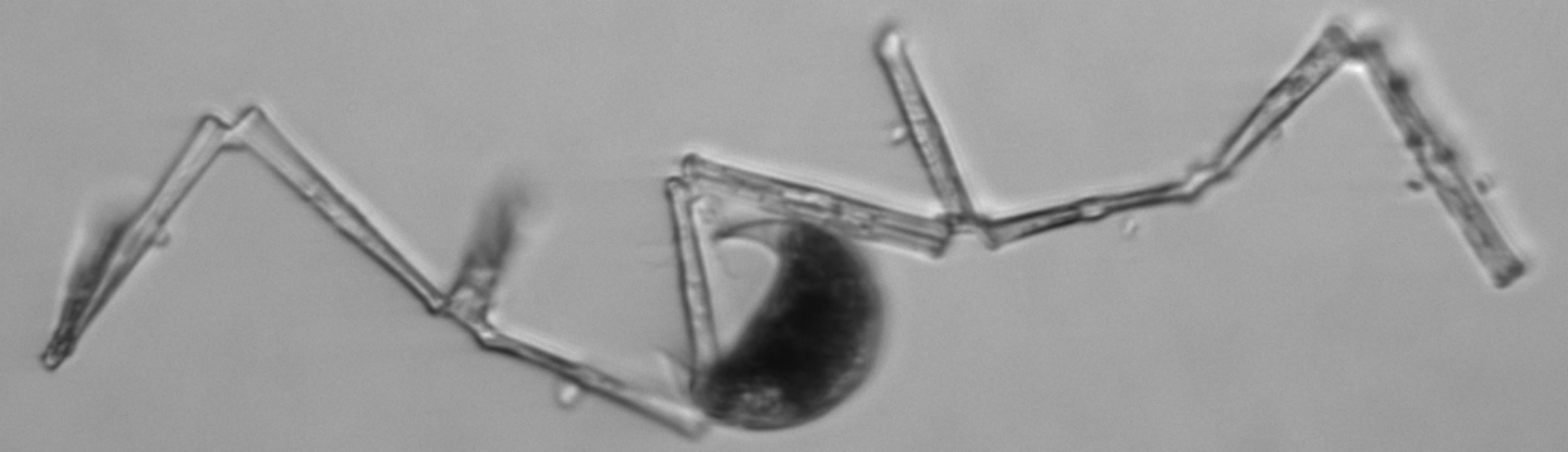

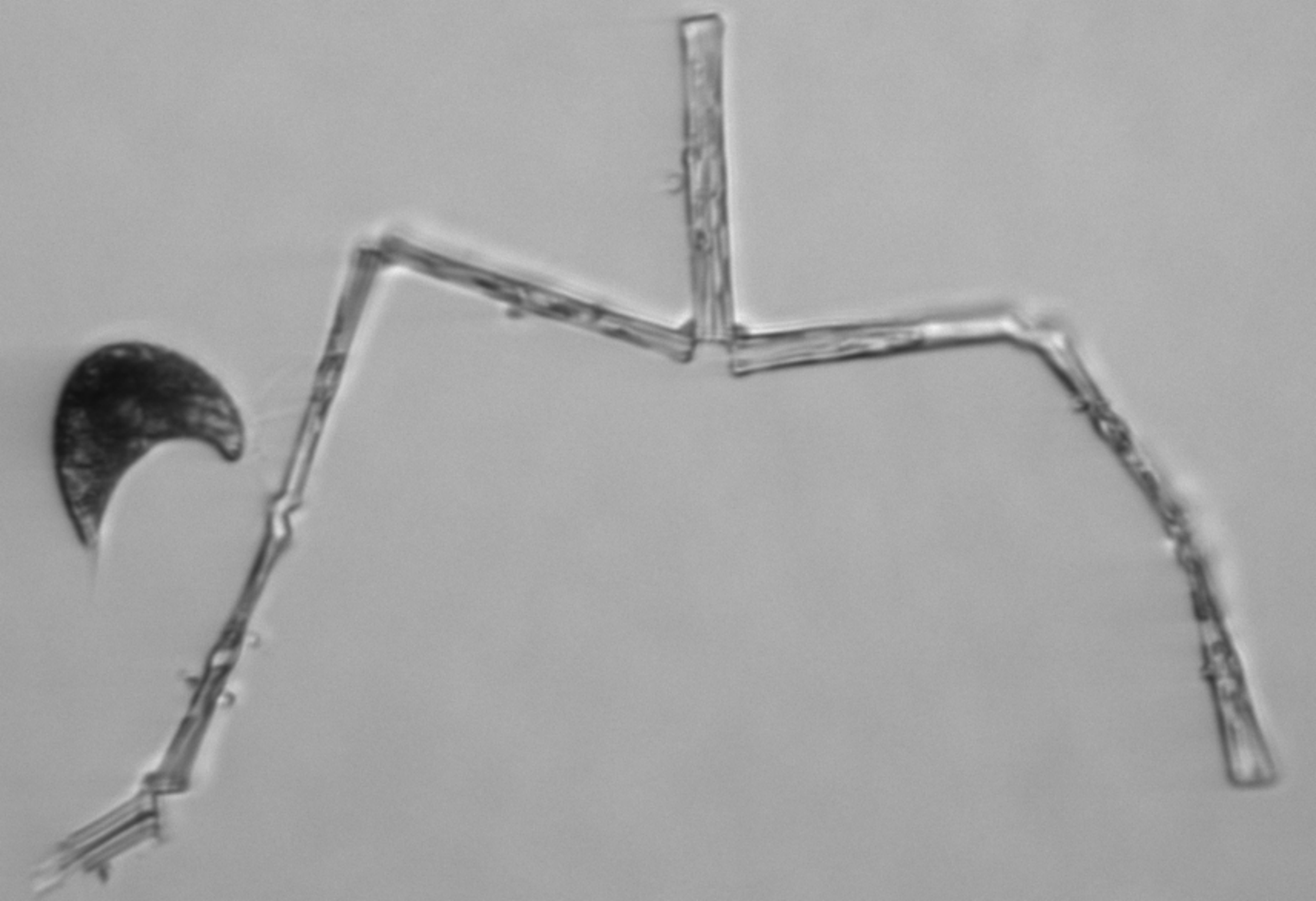

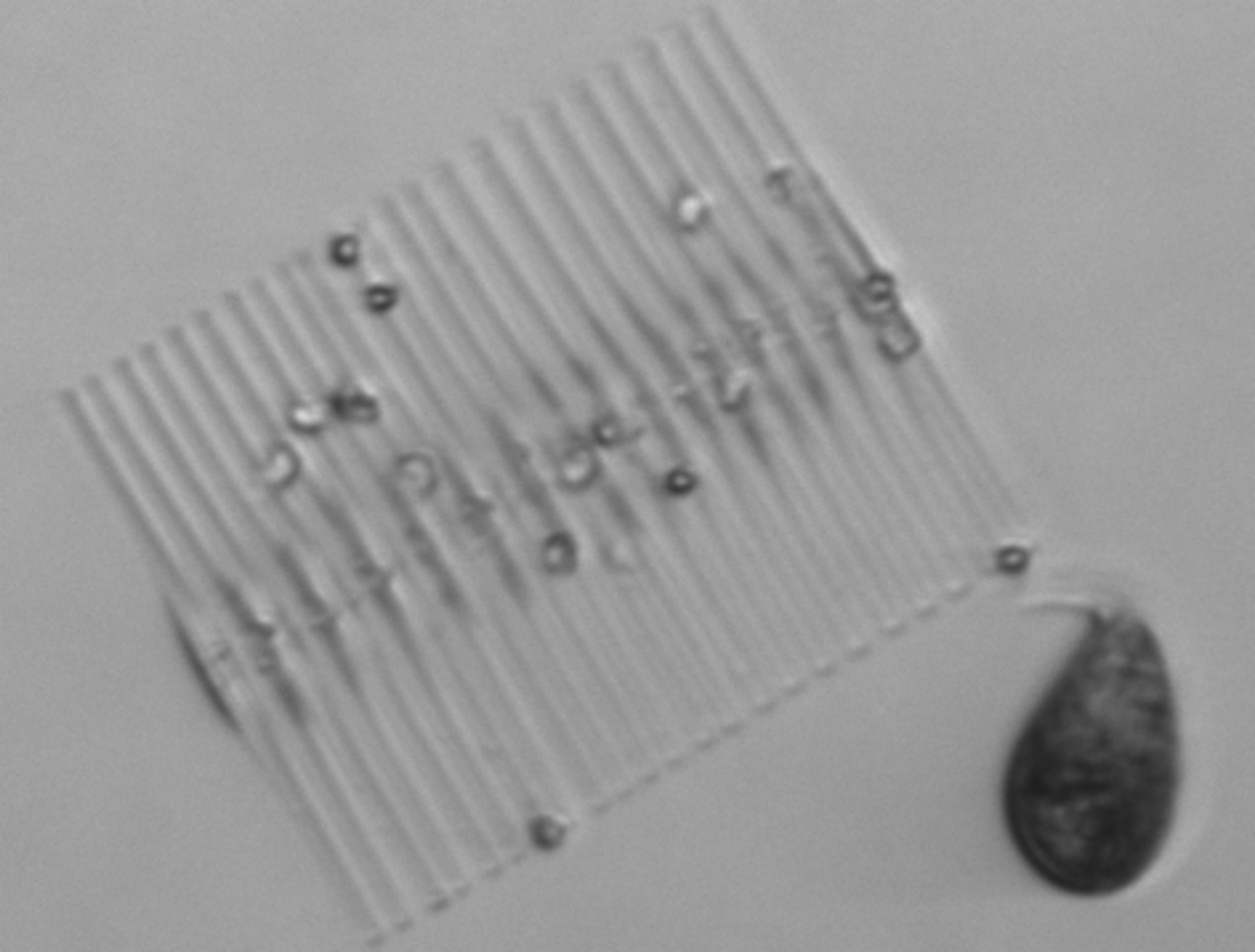

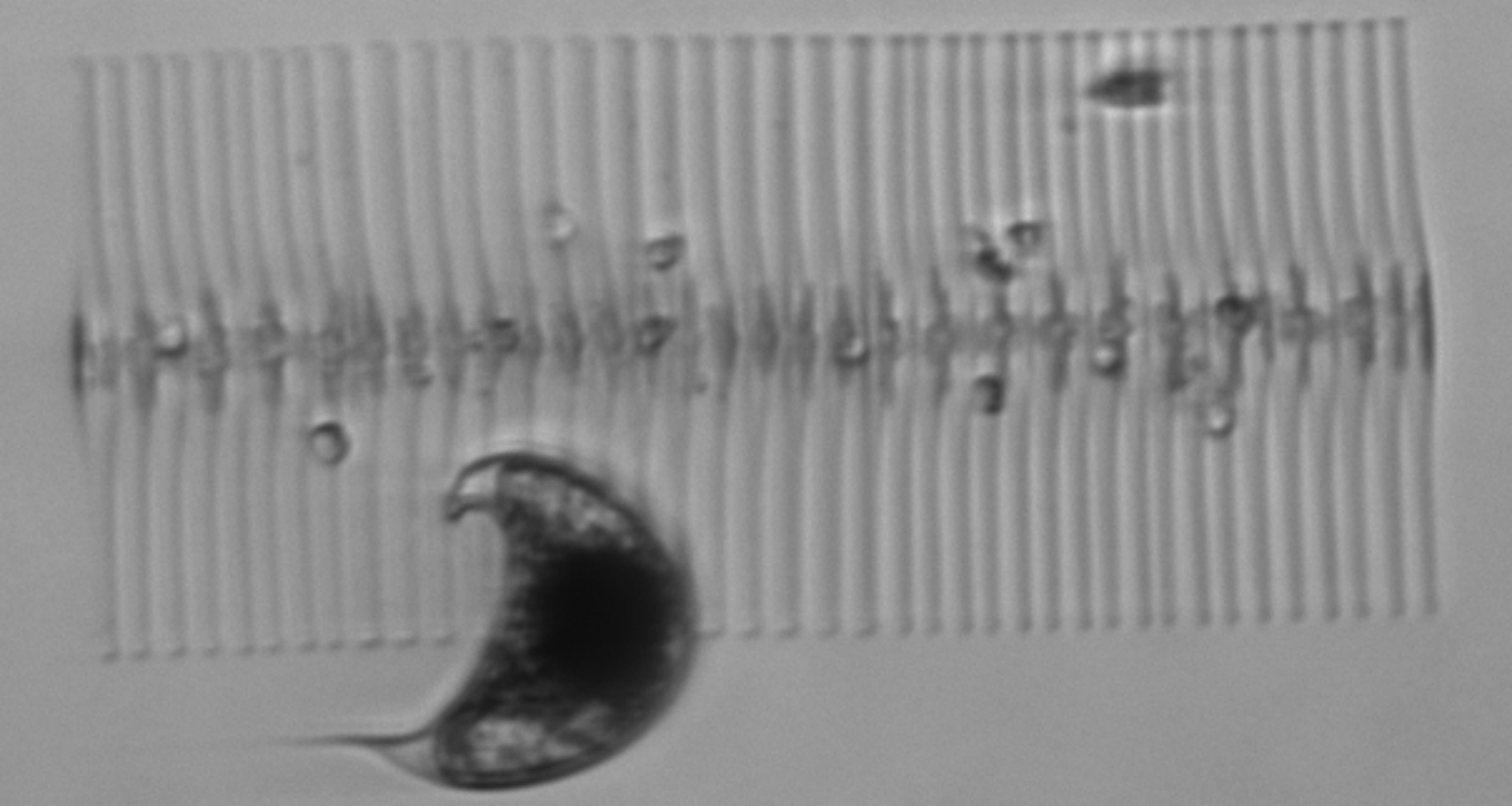

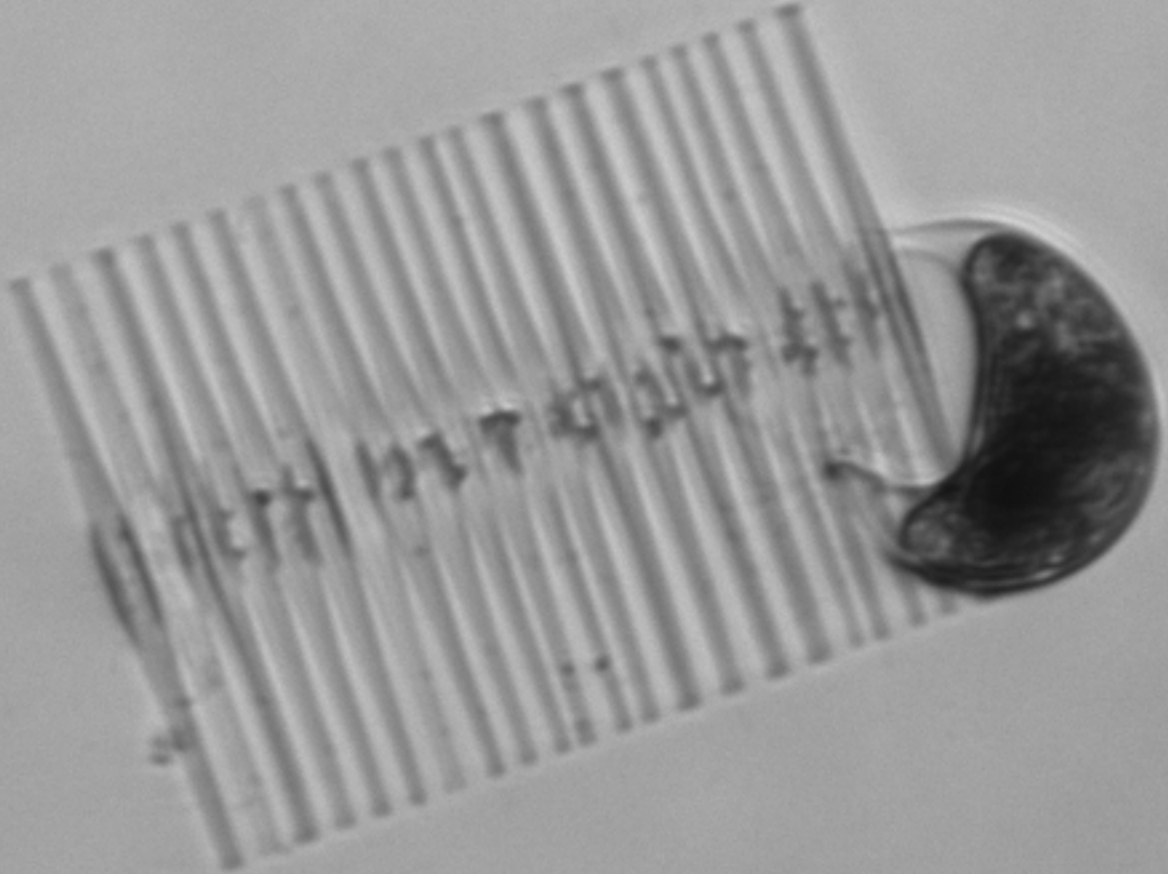

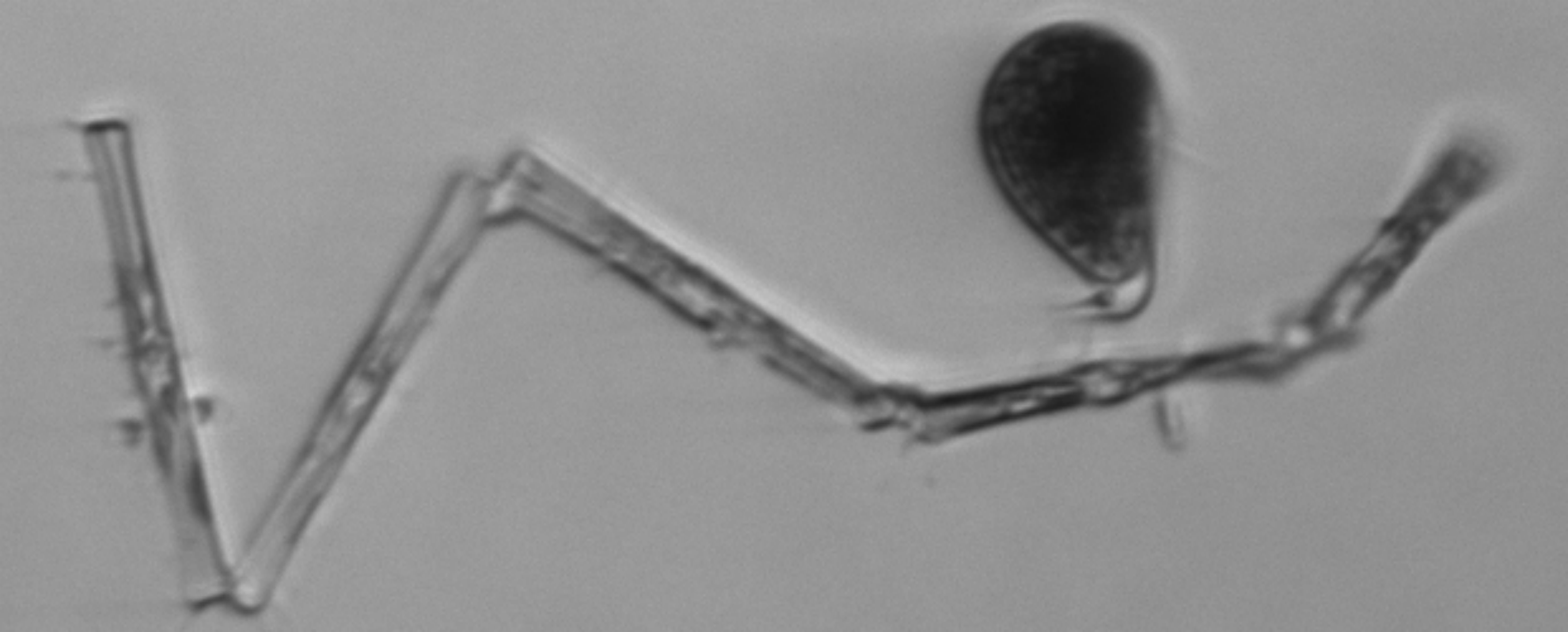

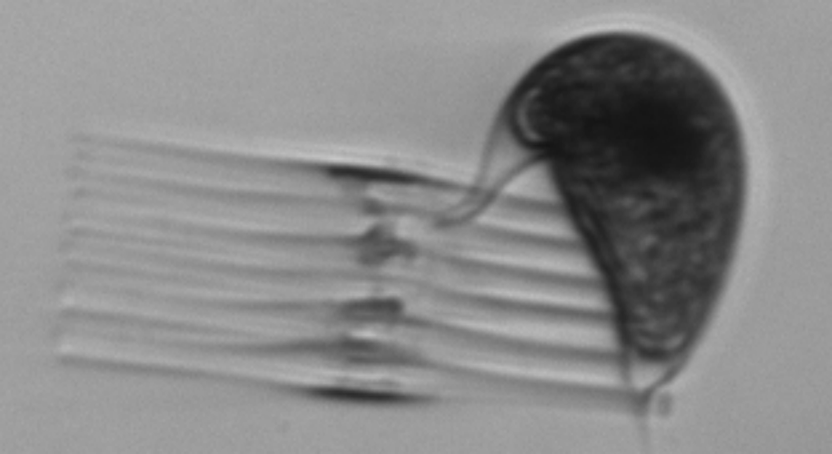

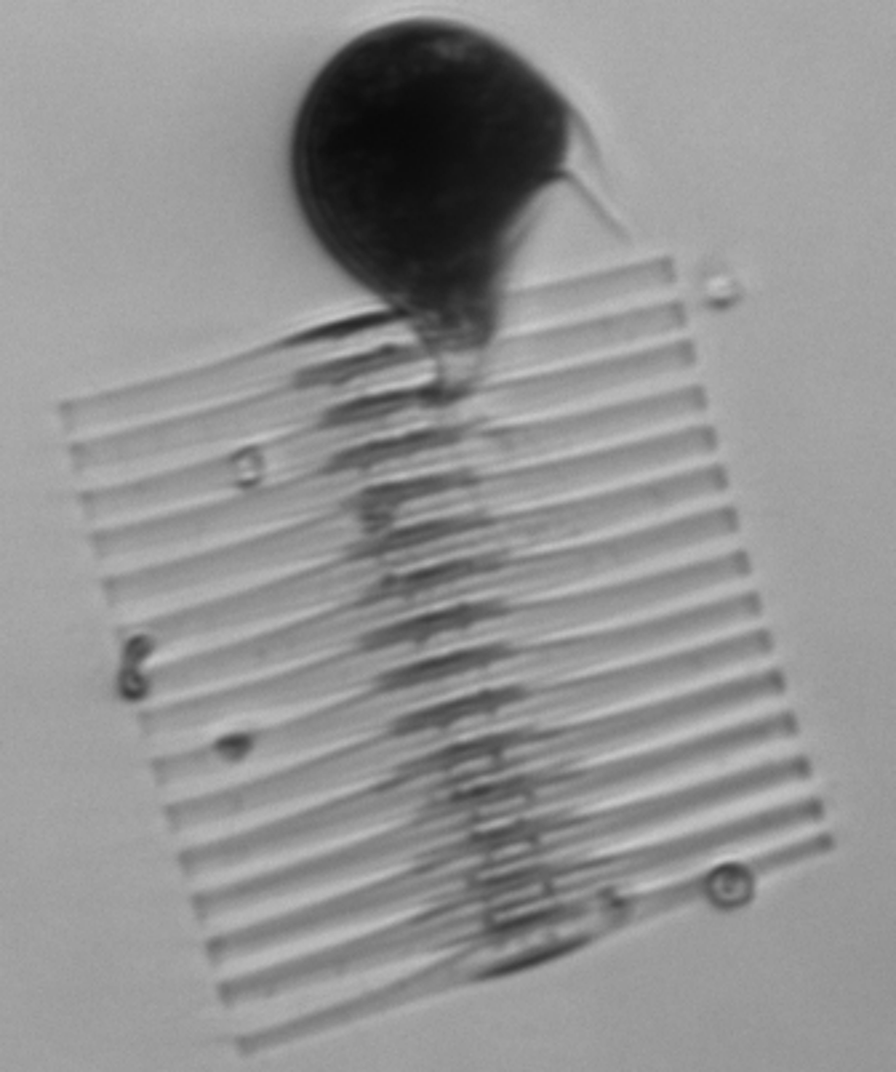

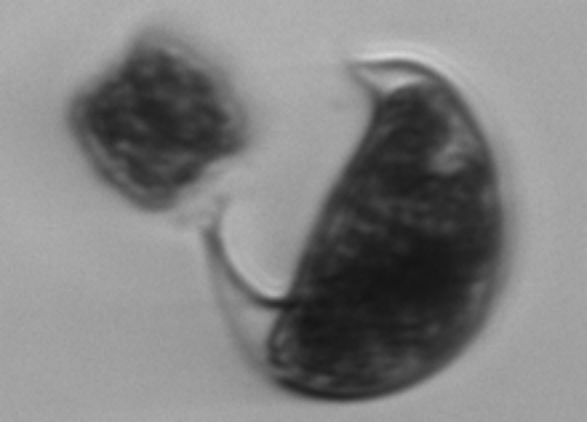

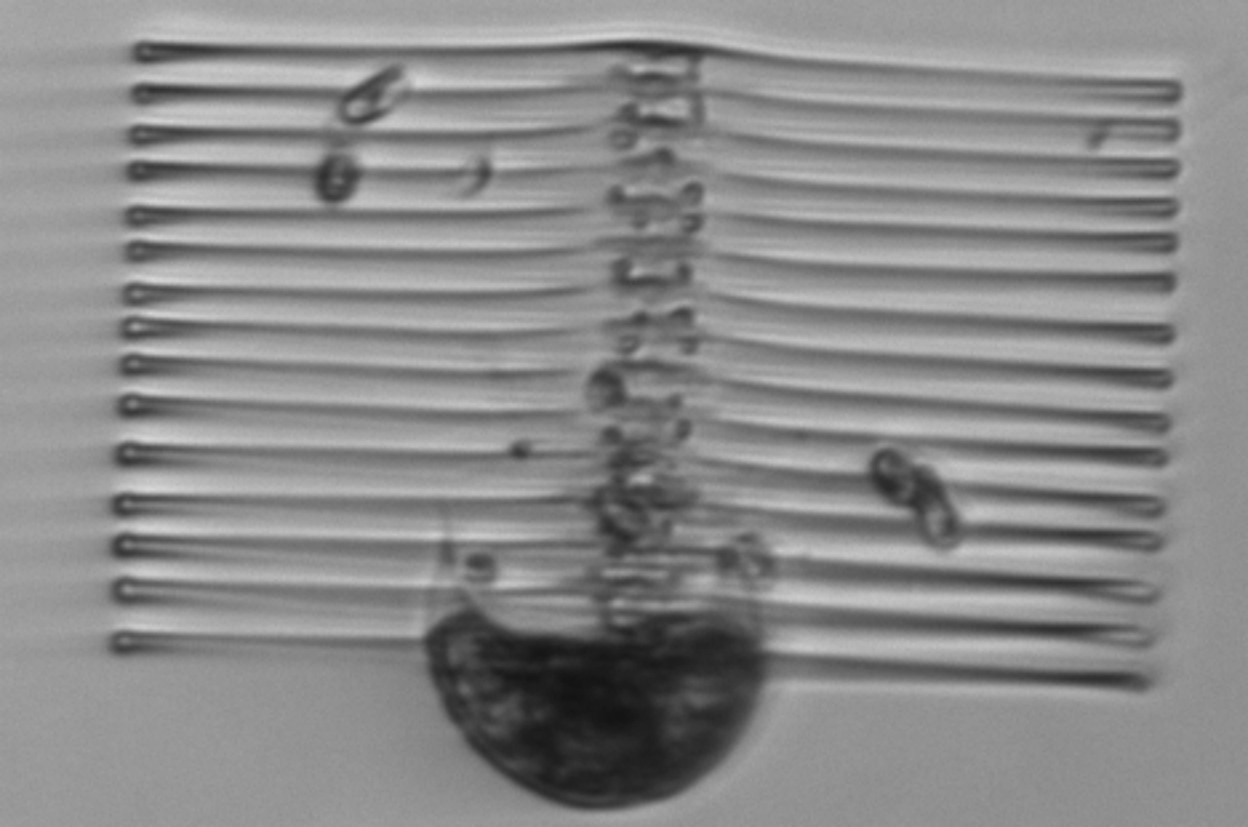

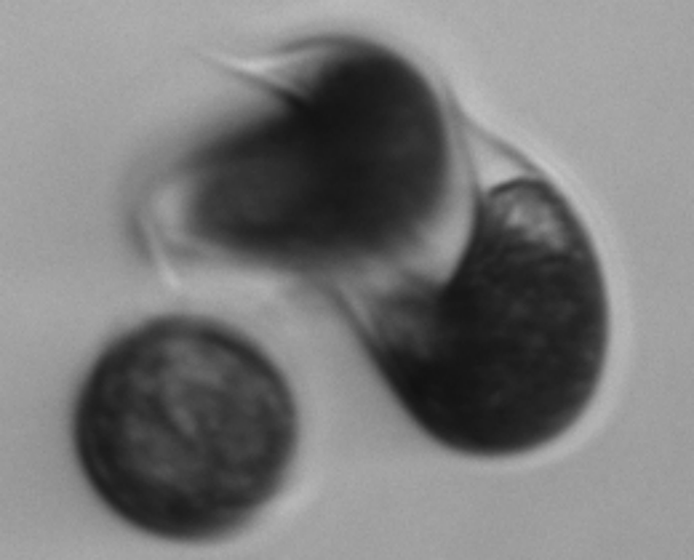

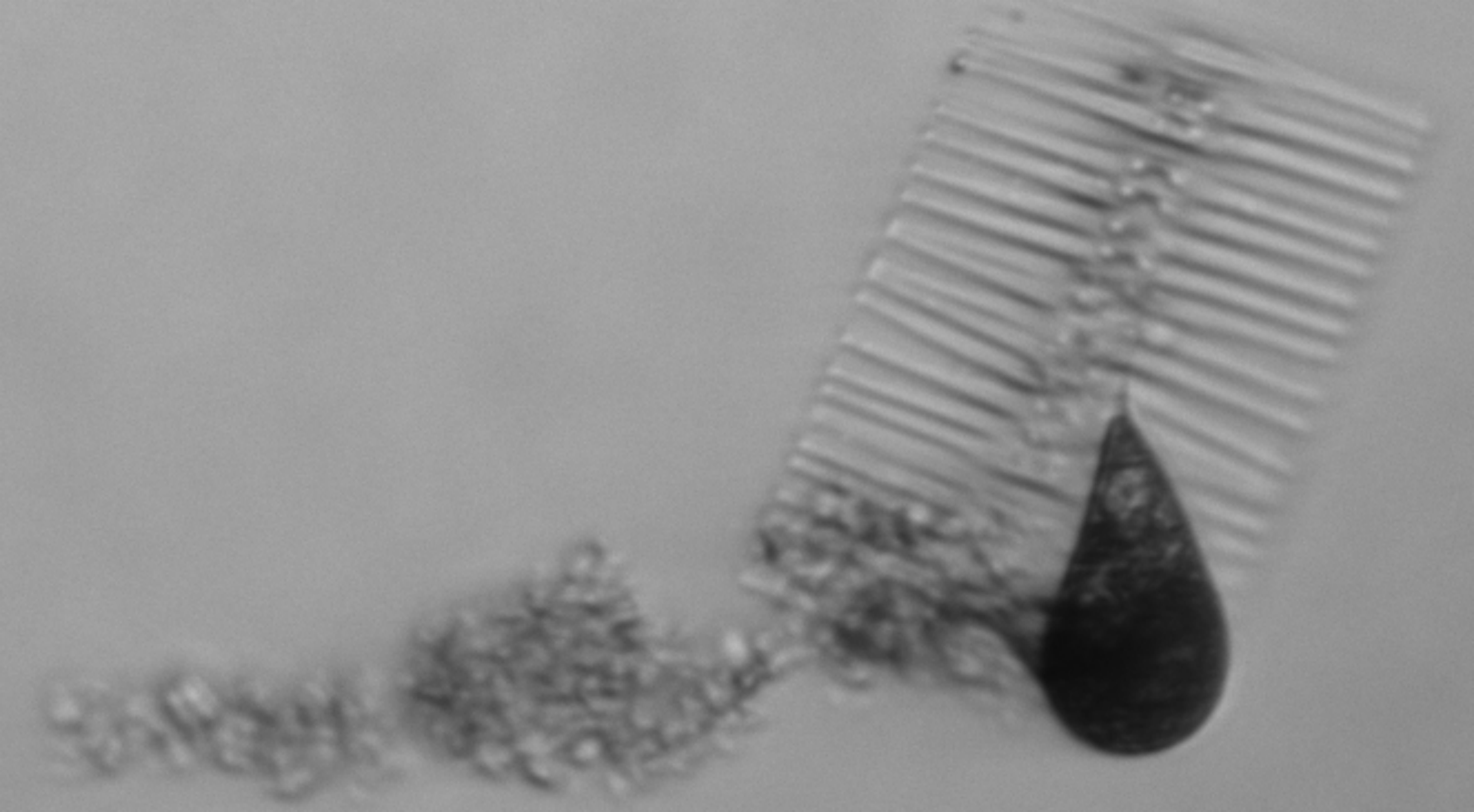

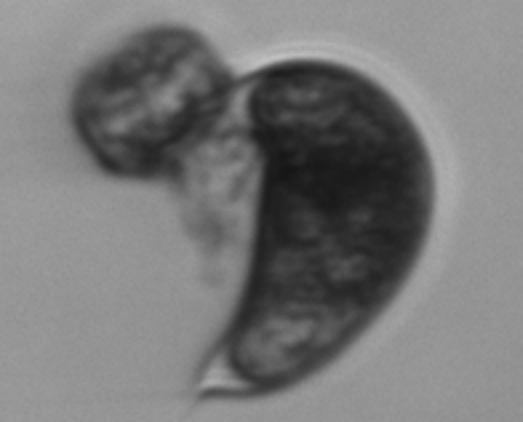

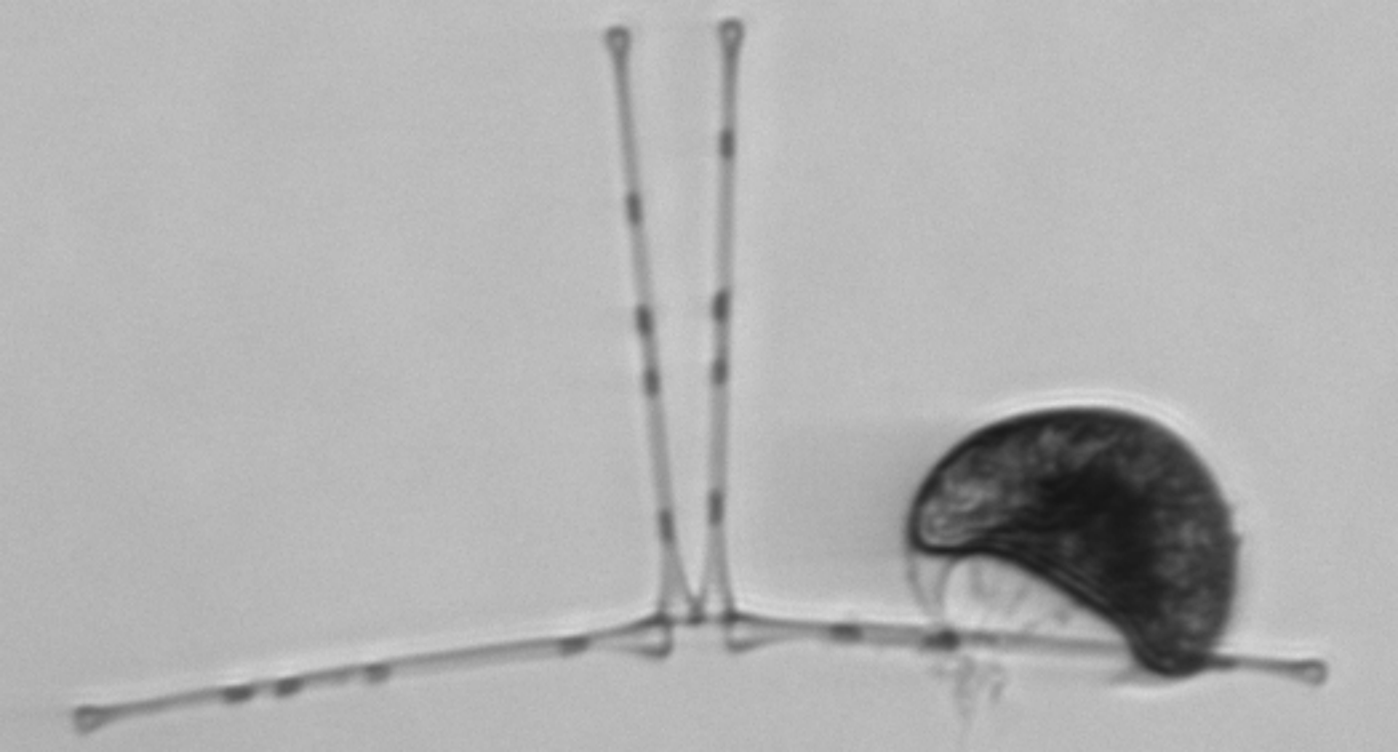

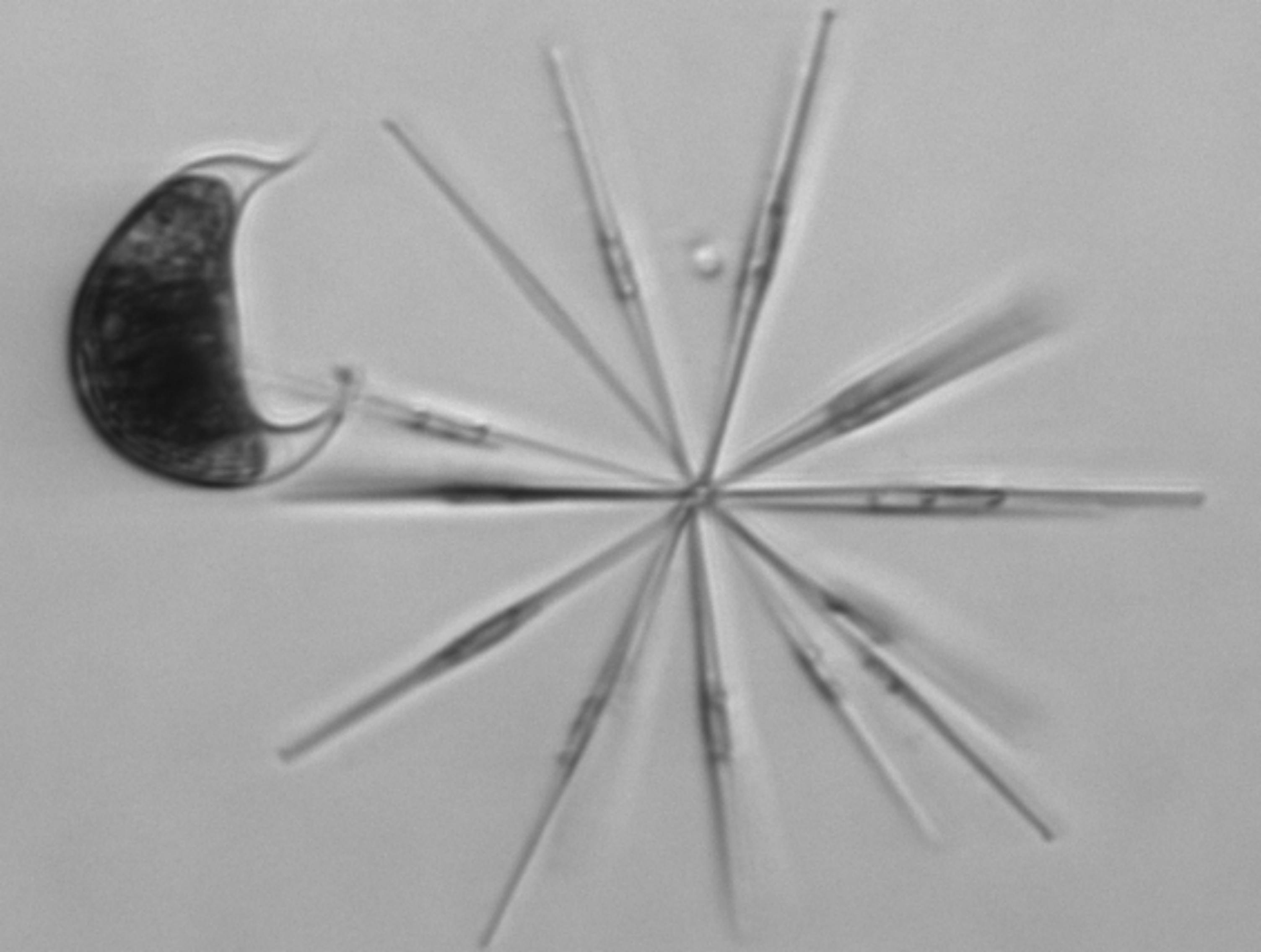

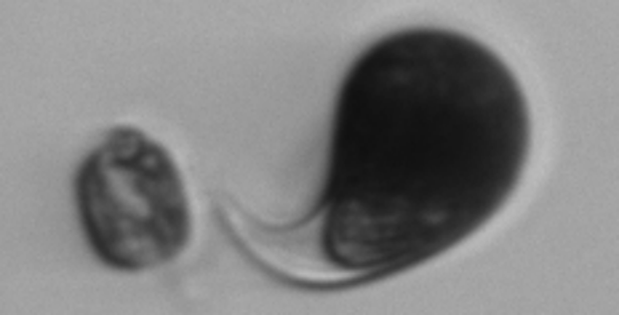

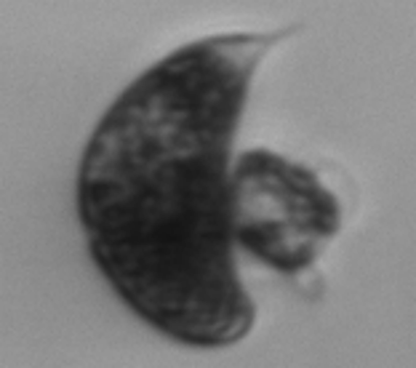

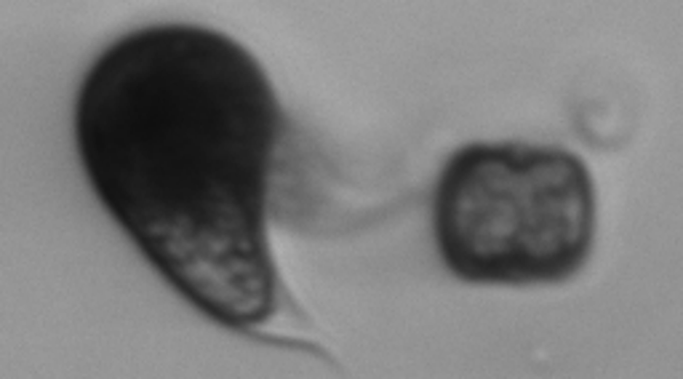

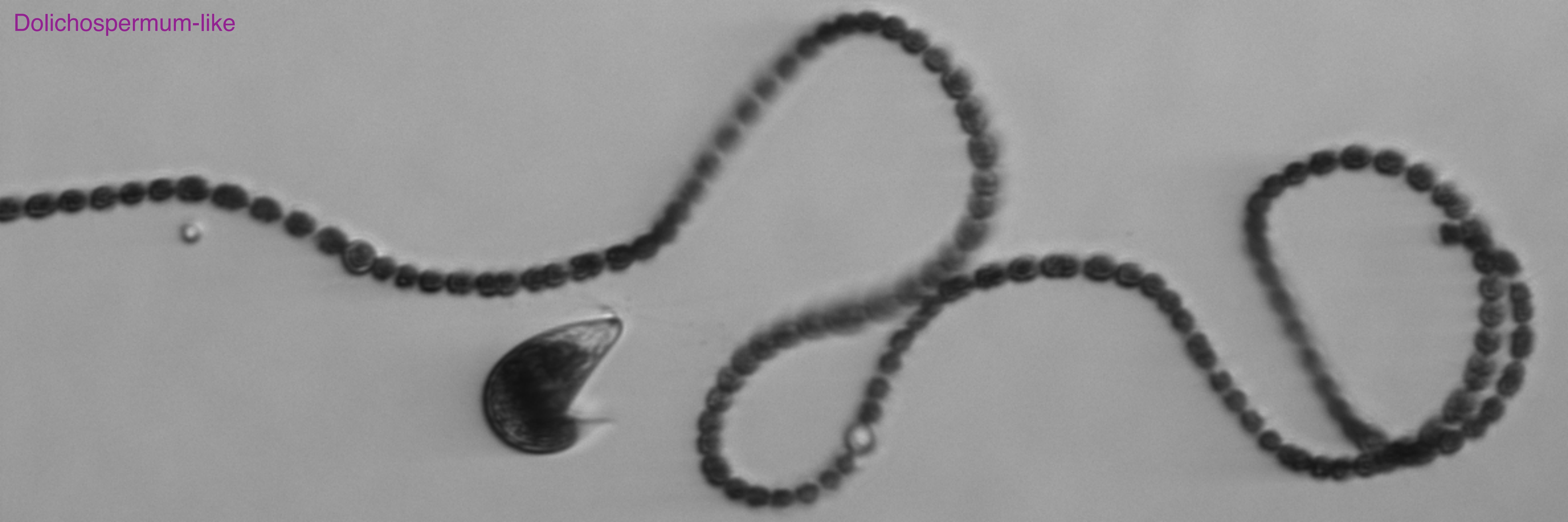
Dolichospermum-like

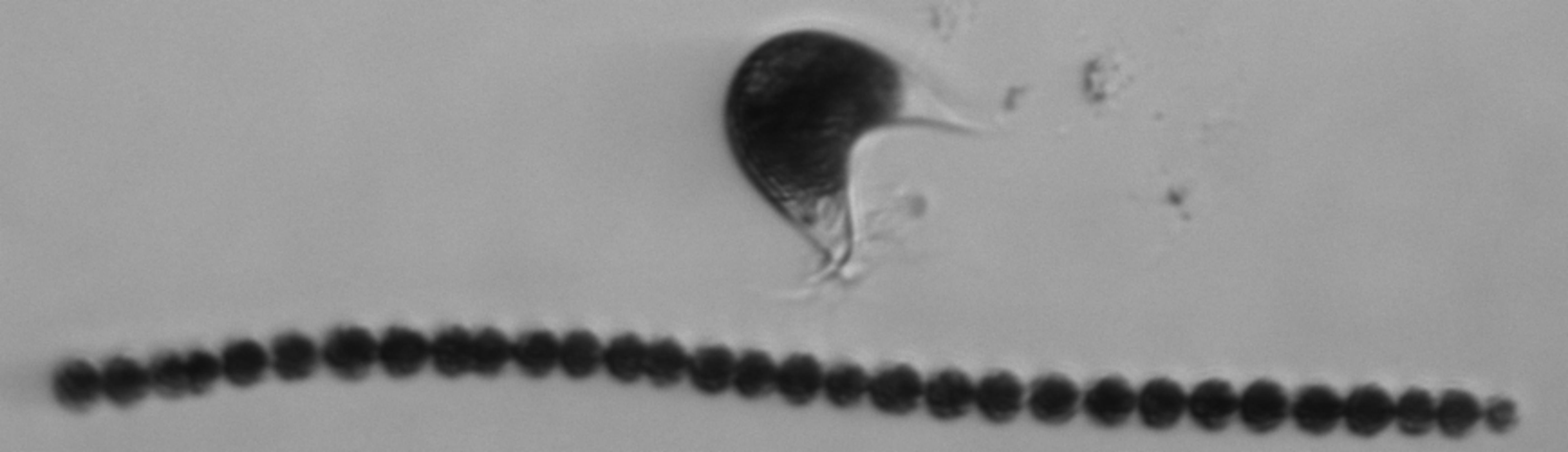

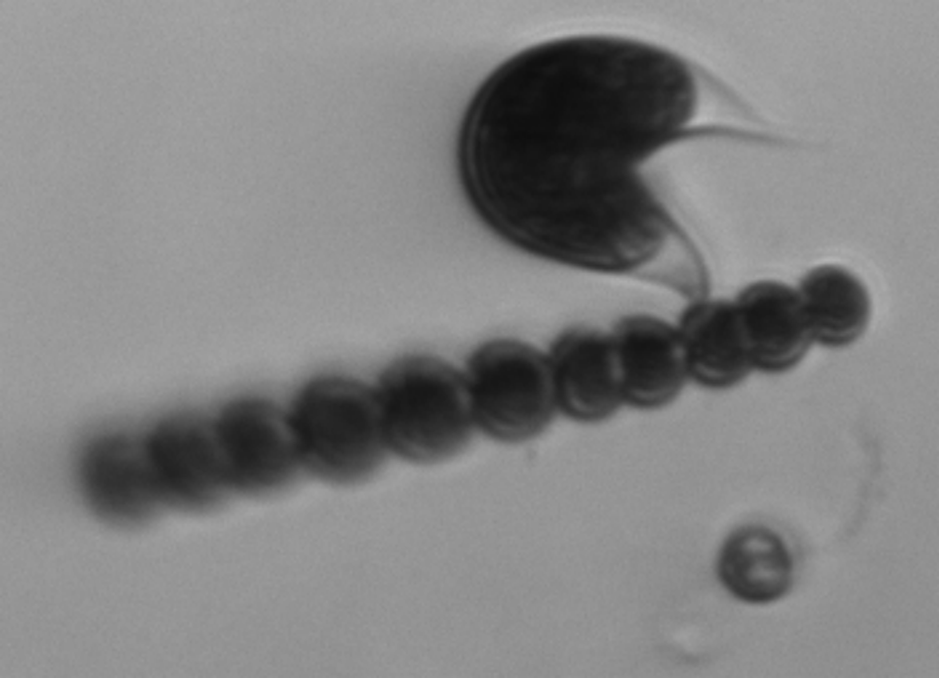

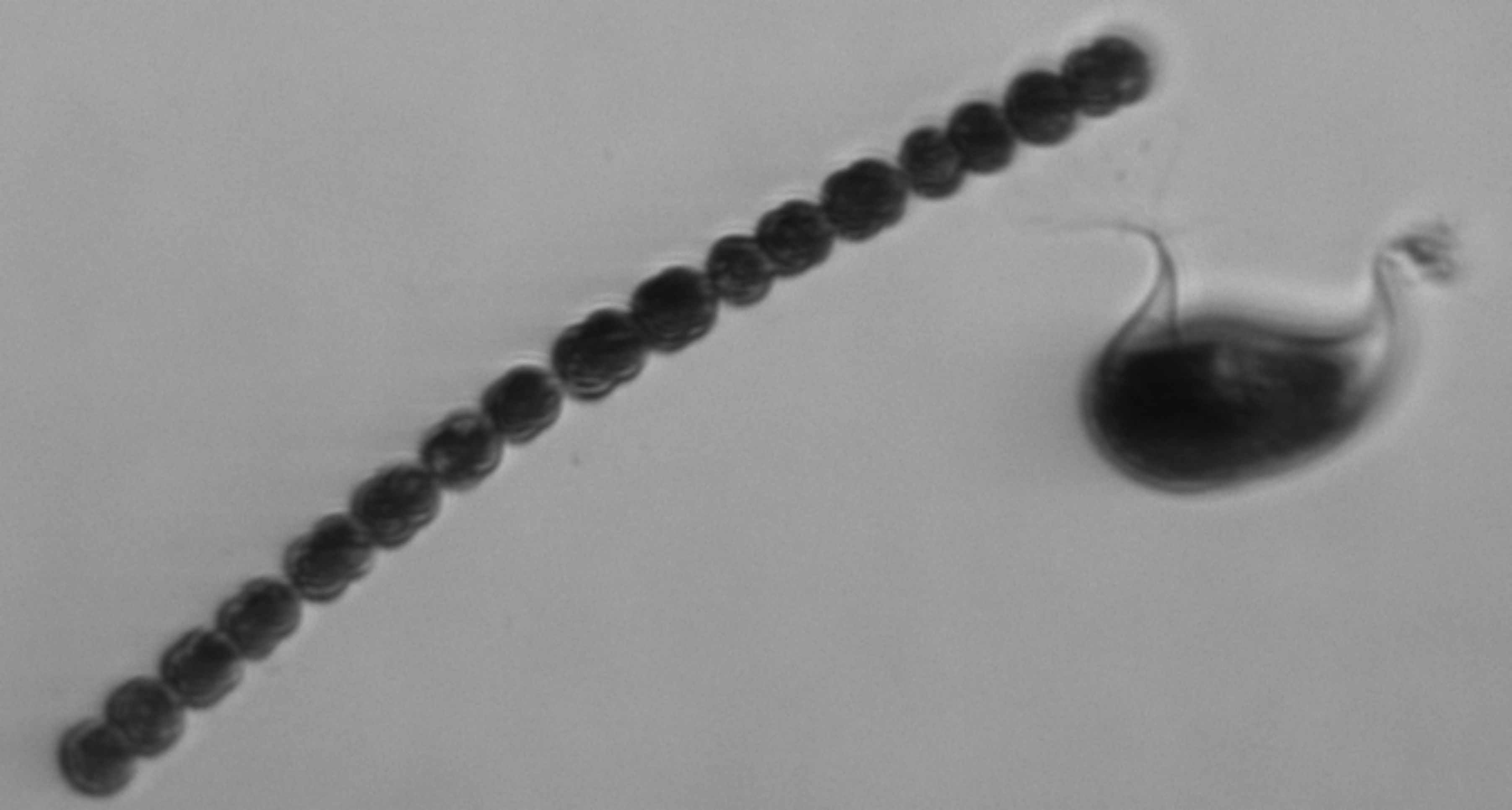

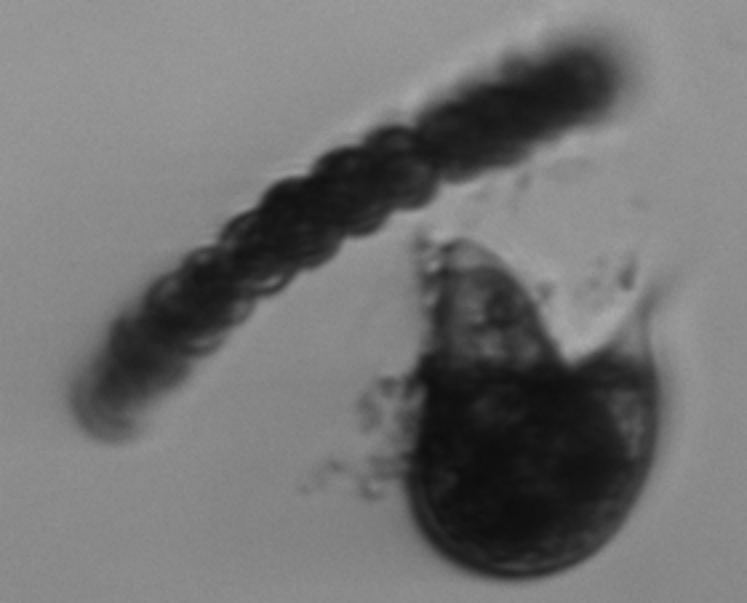

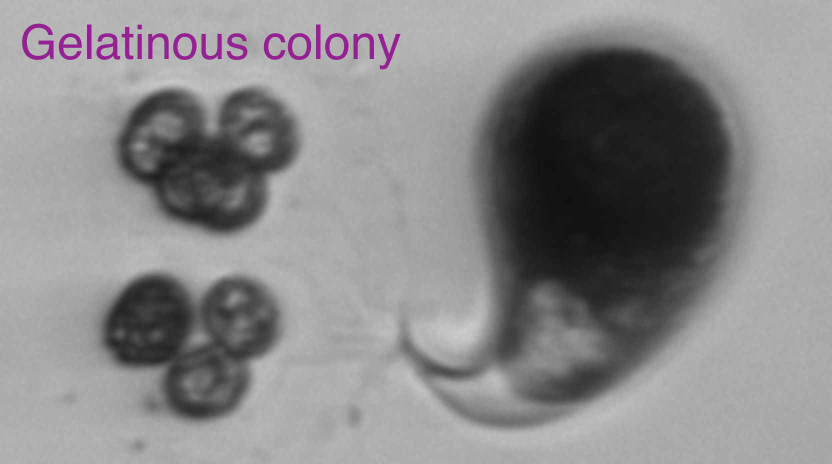
Gelatinous colony

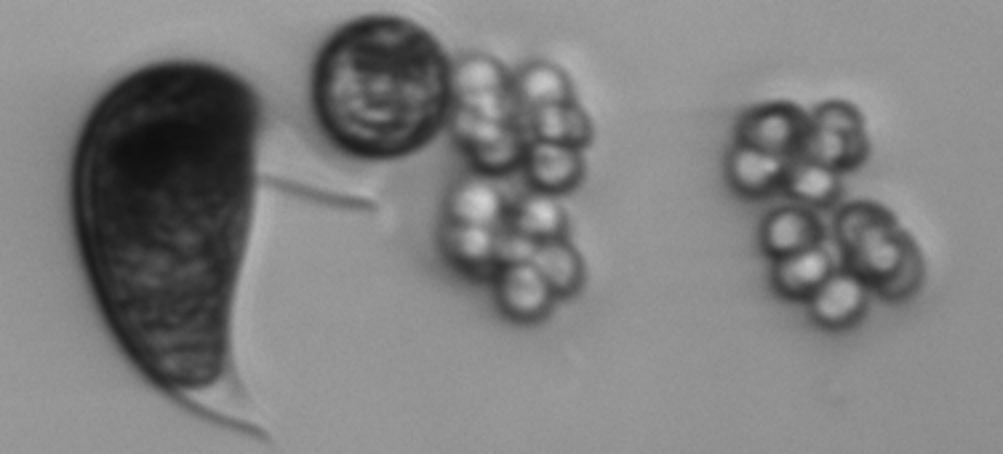

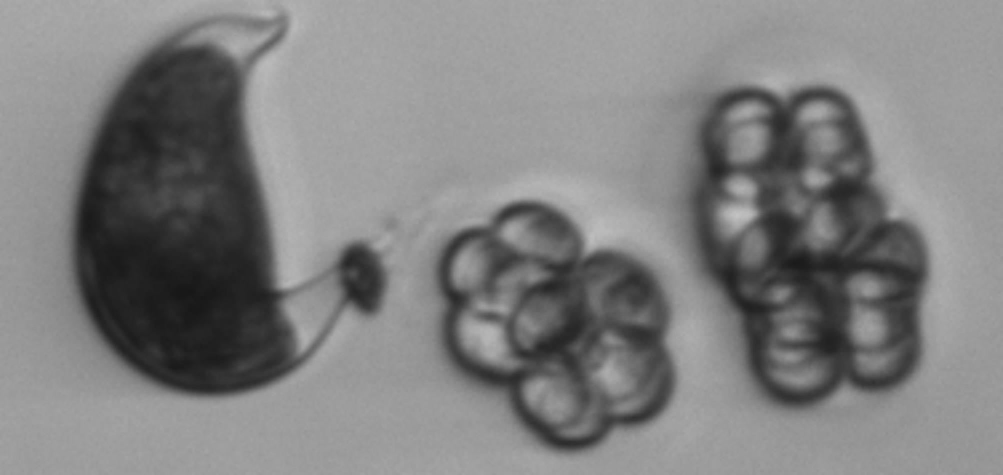

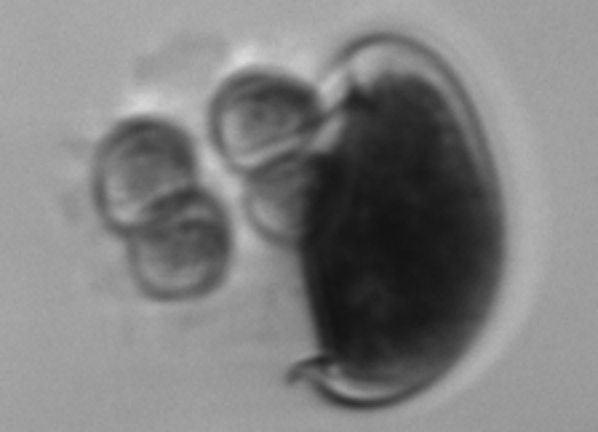

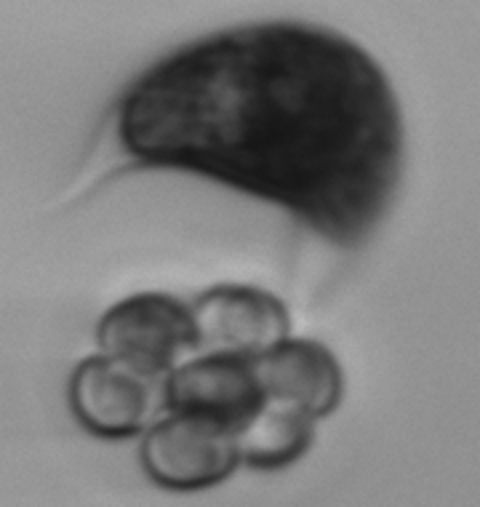

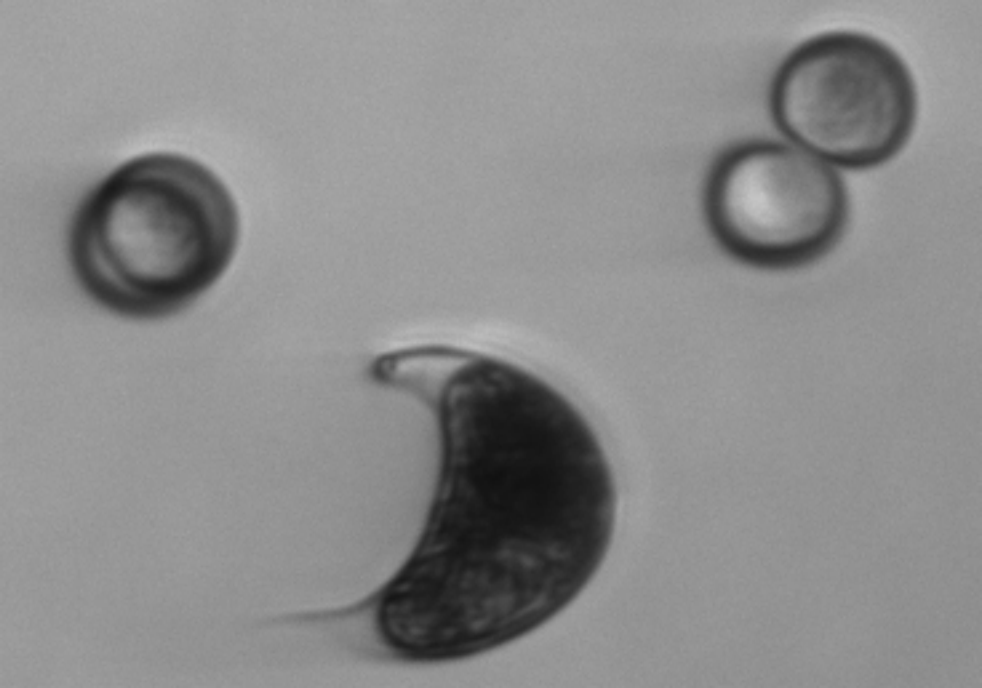

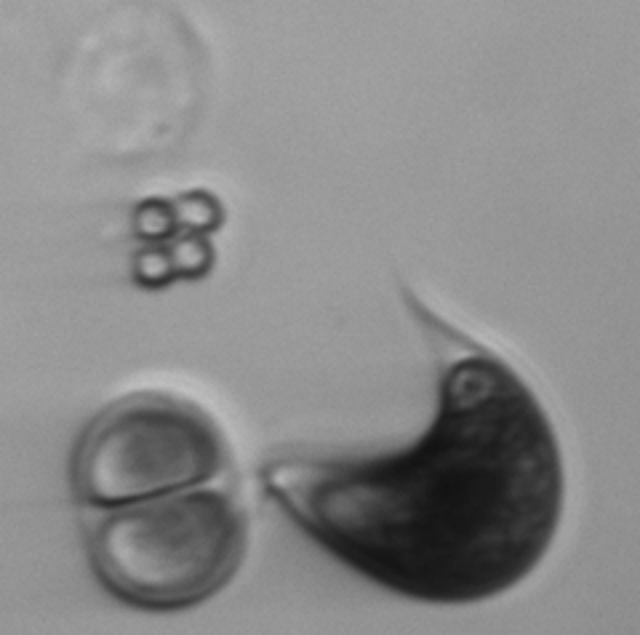

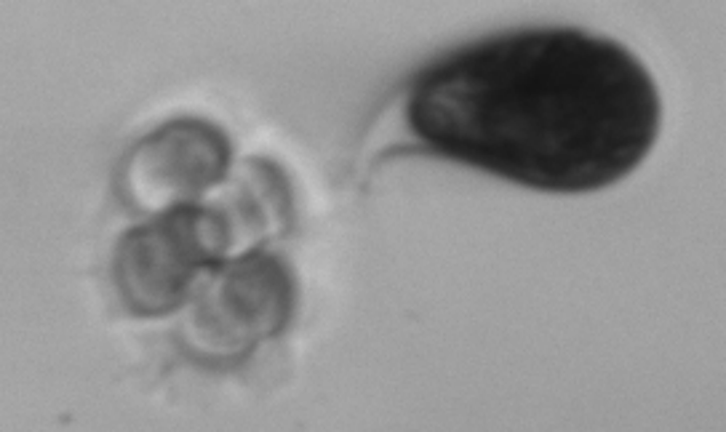

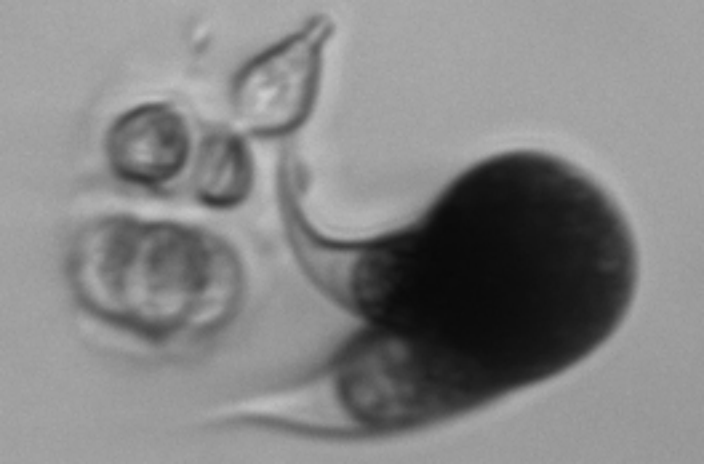

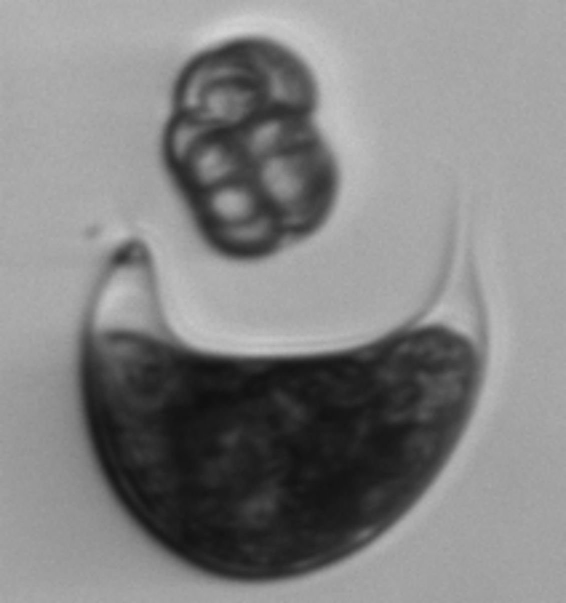

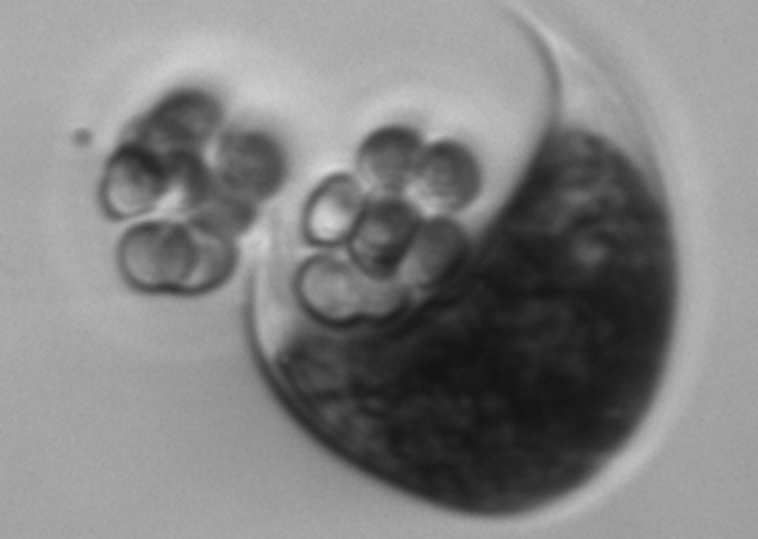

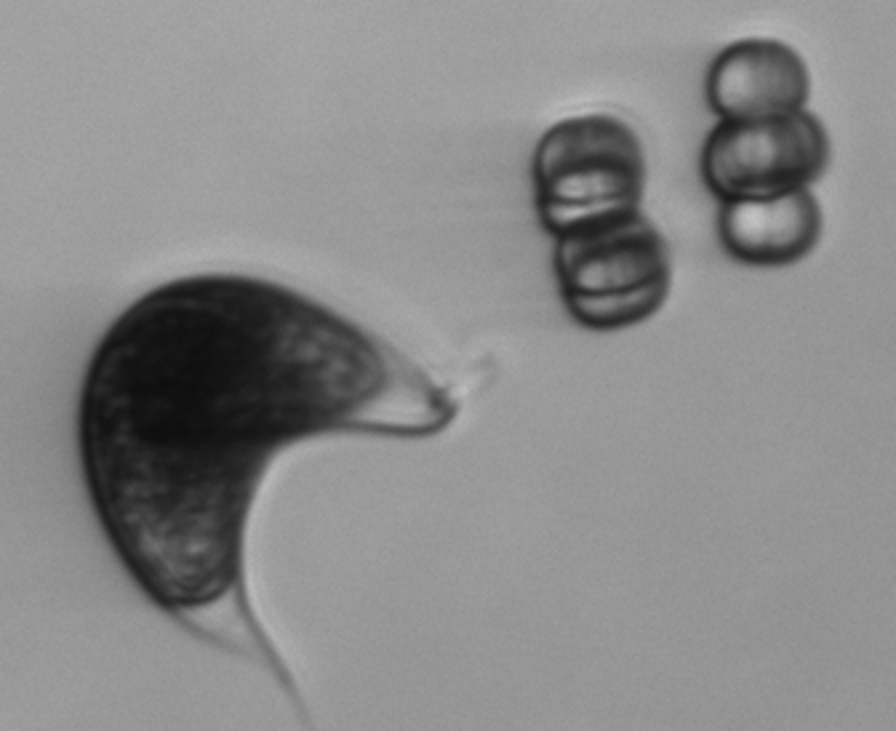

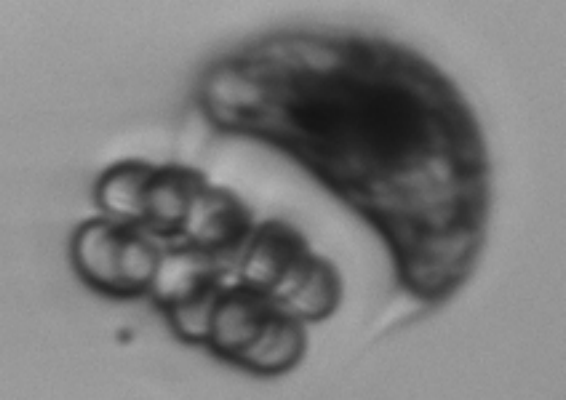

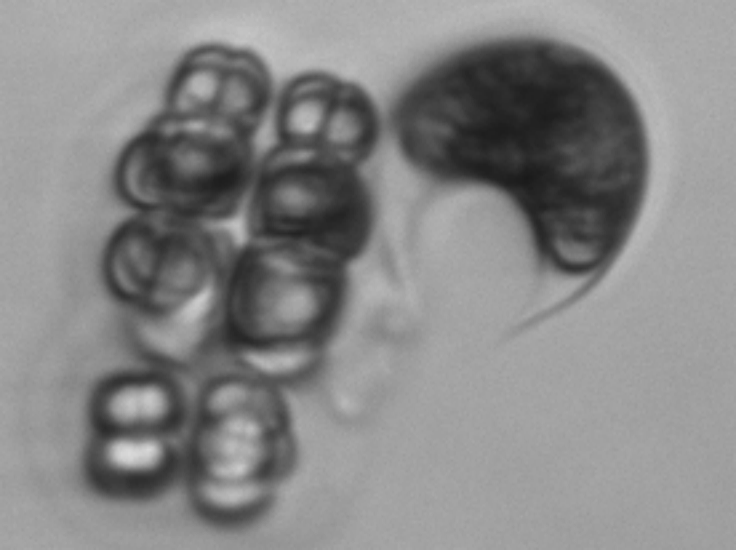

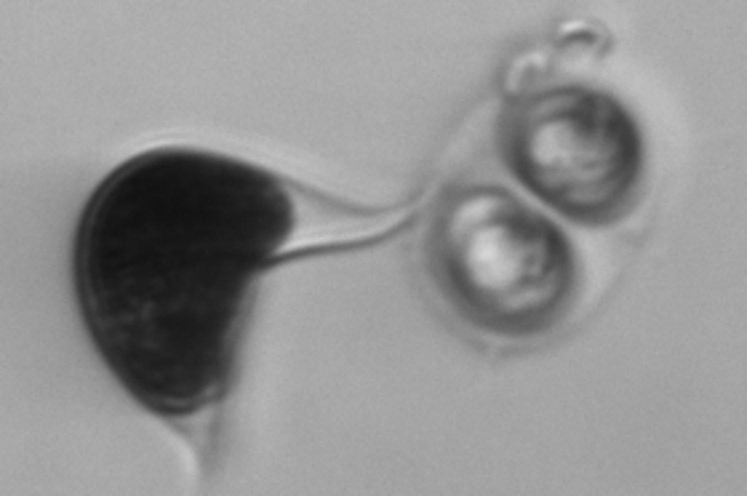

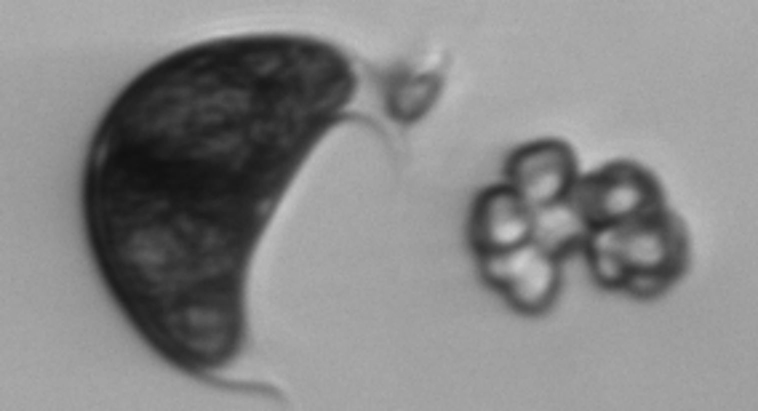

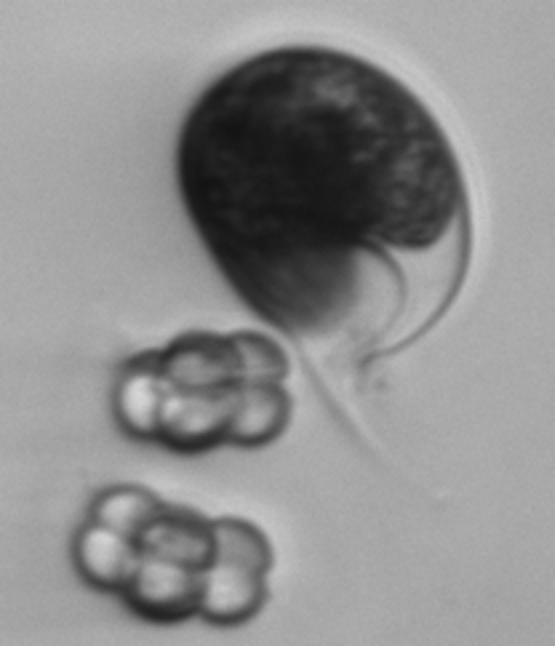

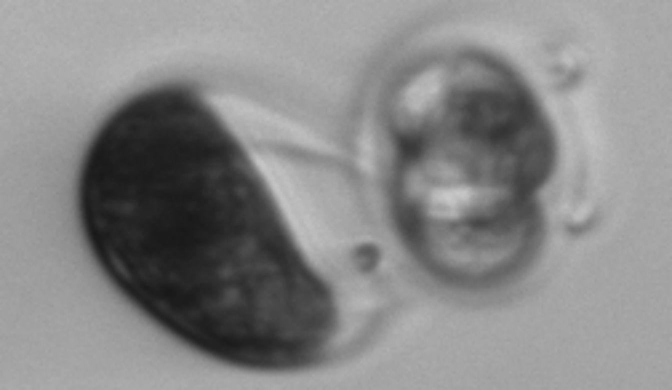

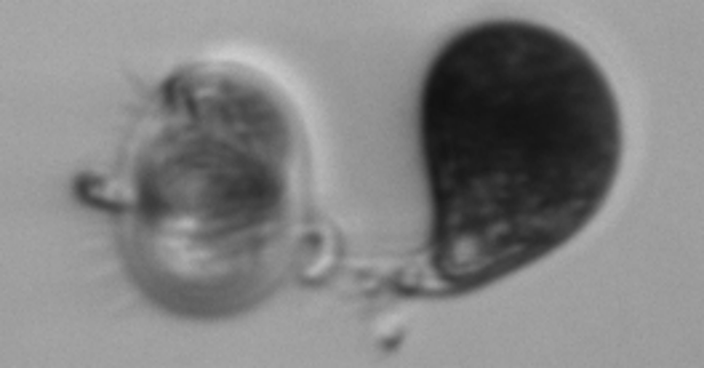

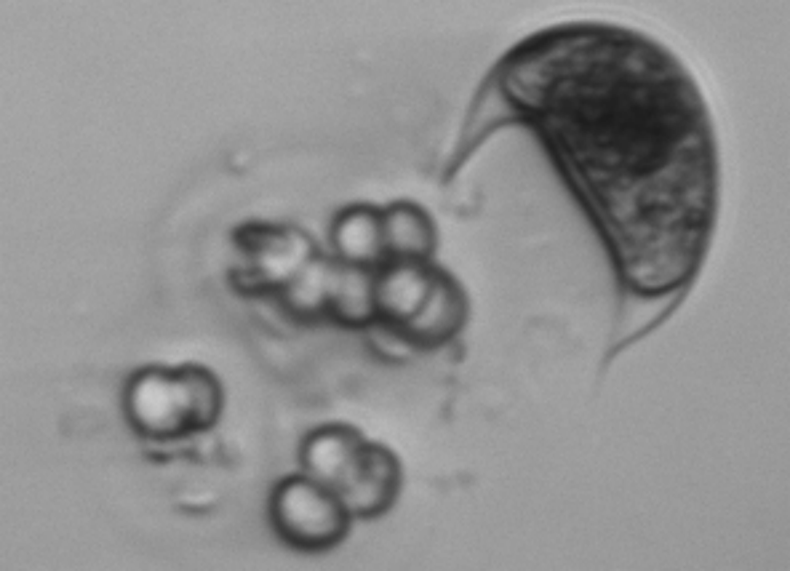

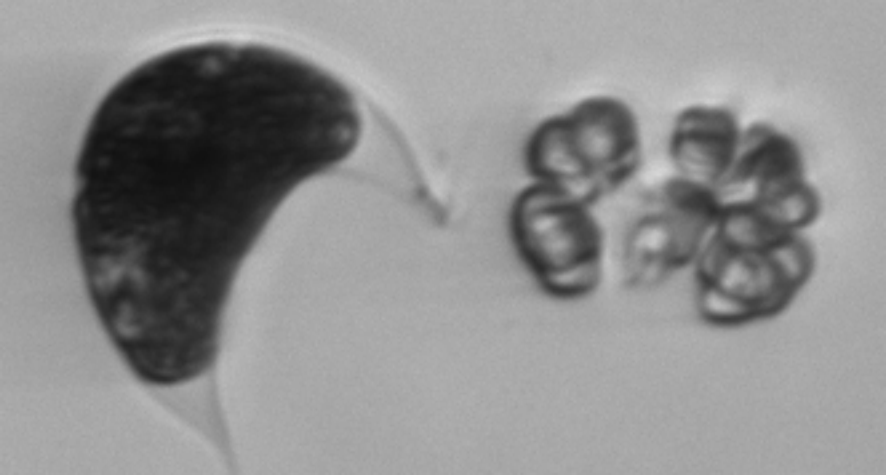

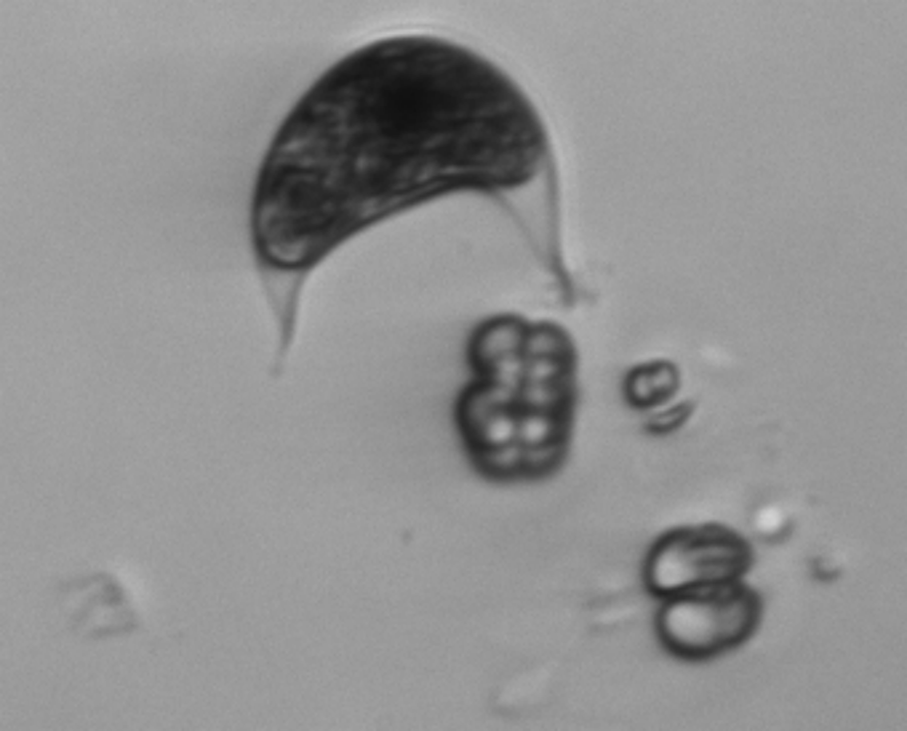

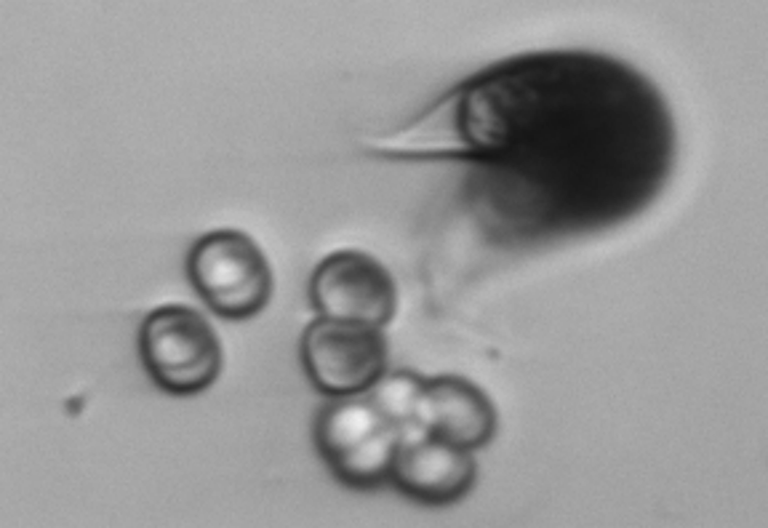

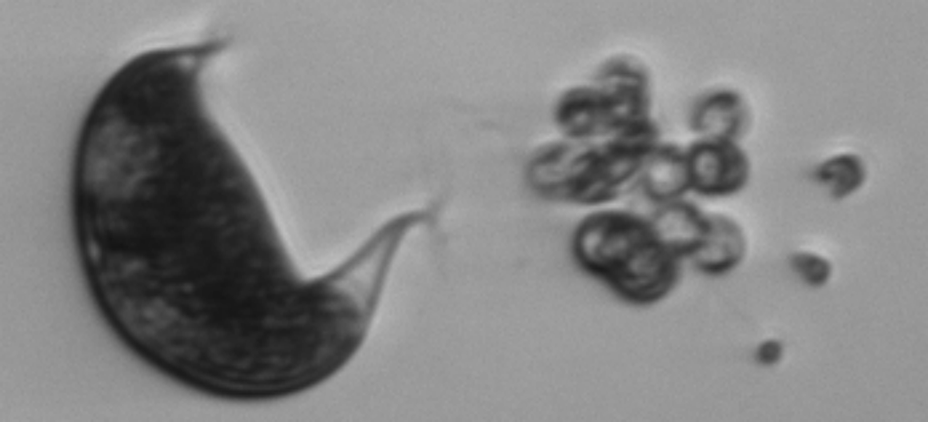

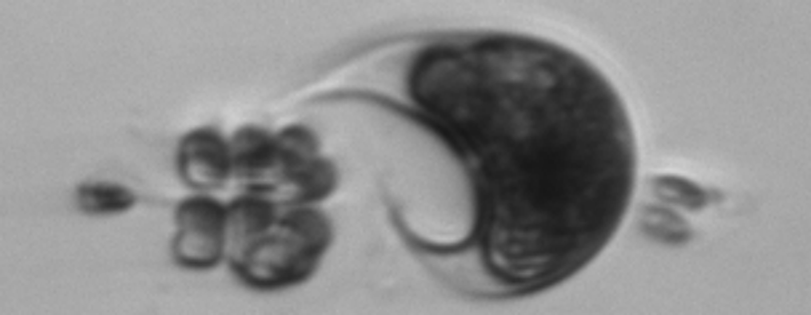

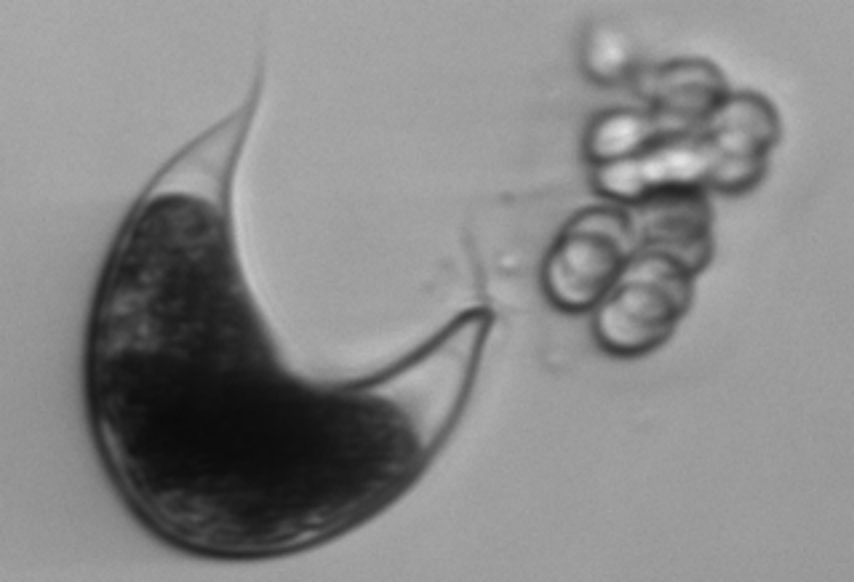

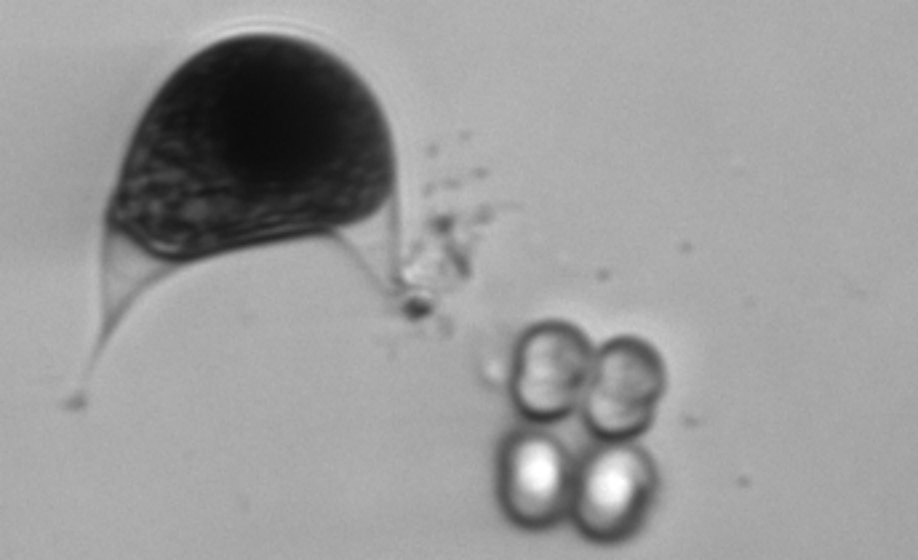

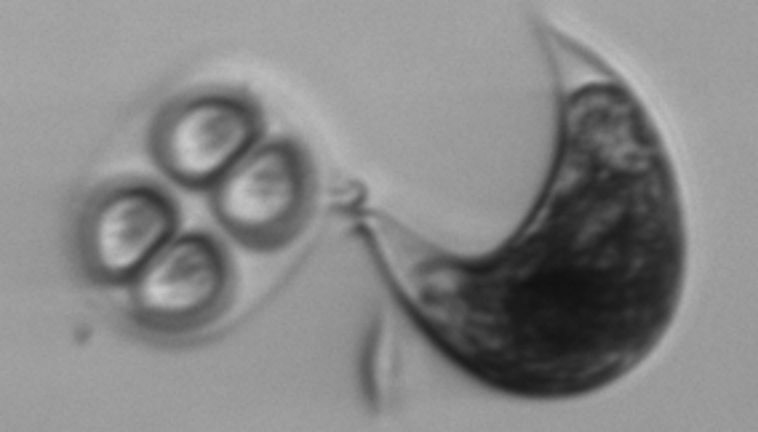

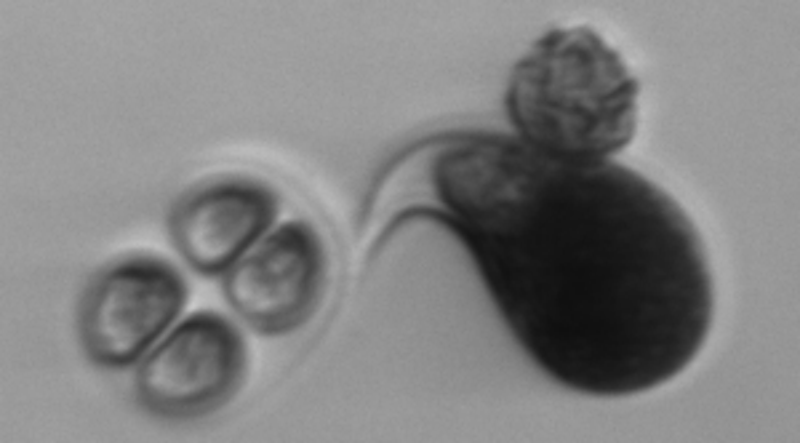

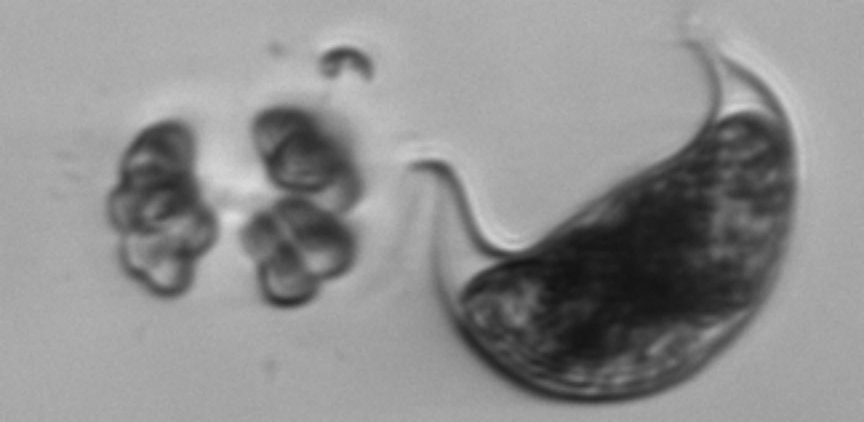

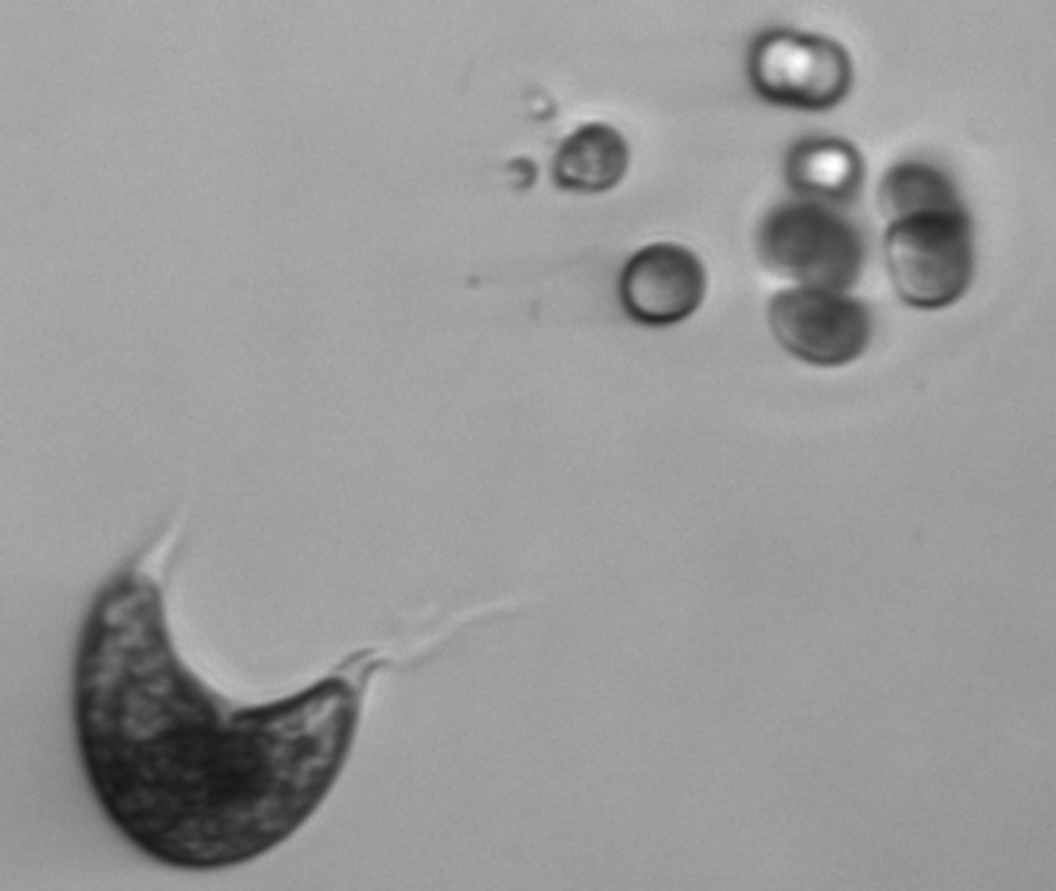

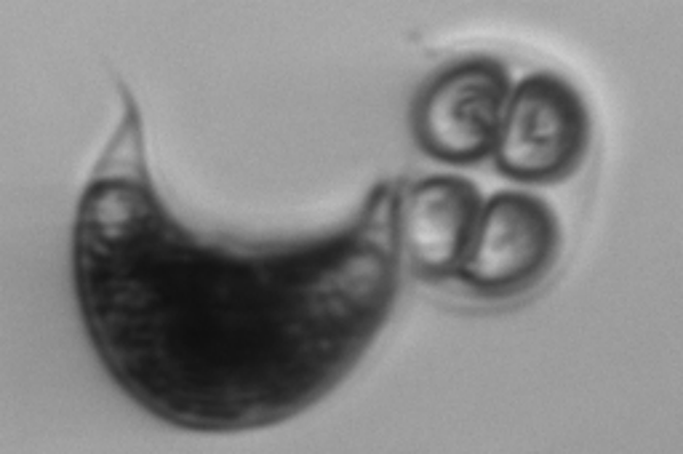

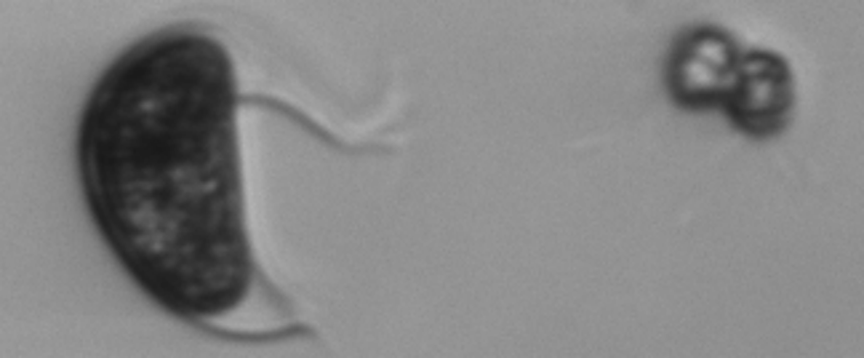

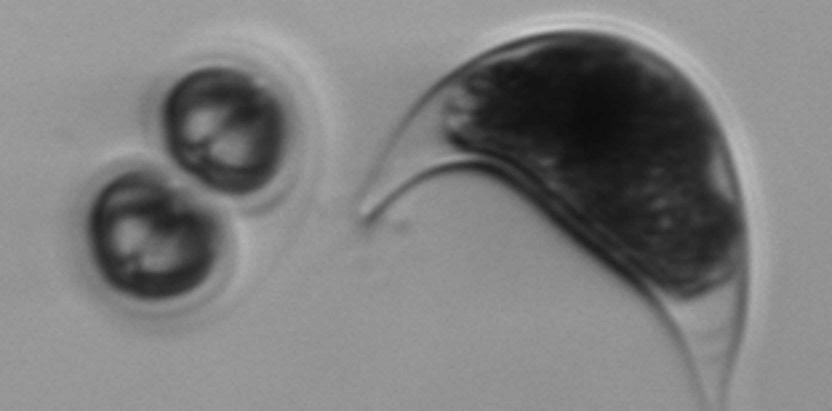

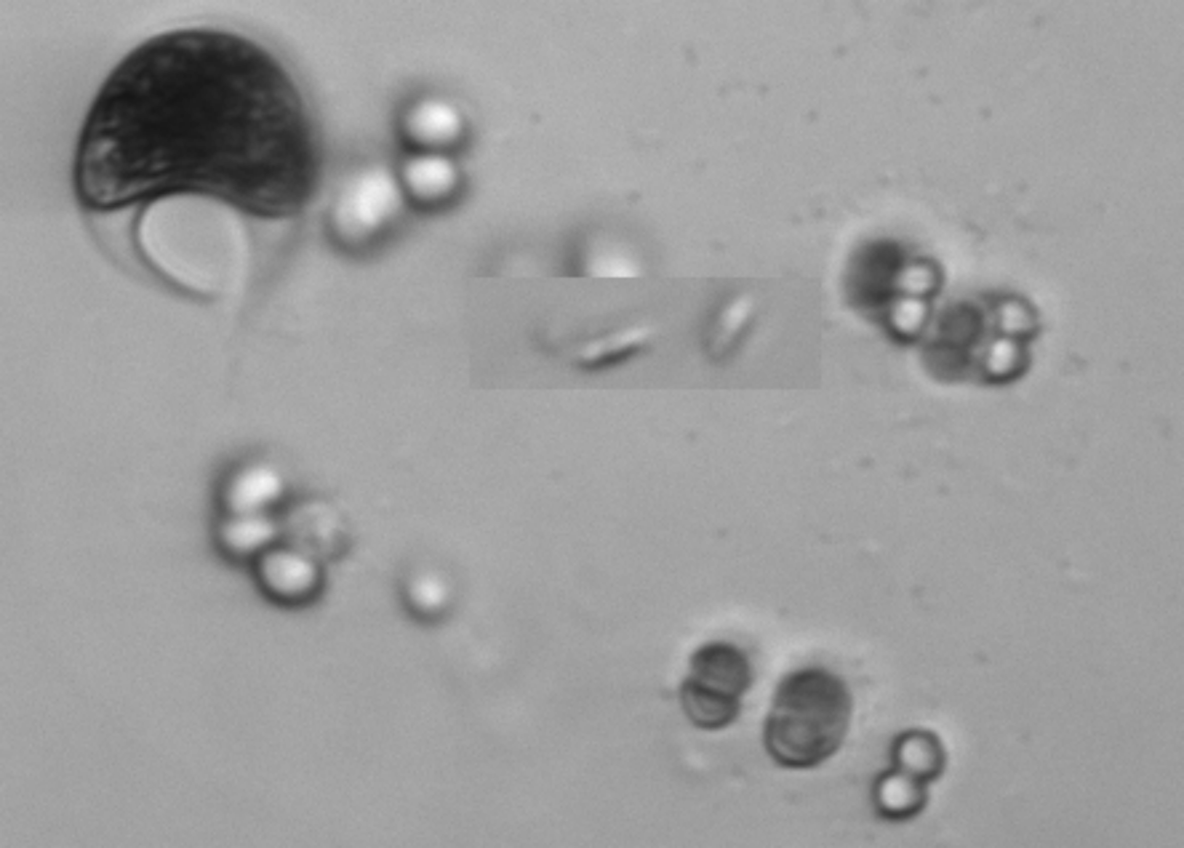

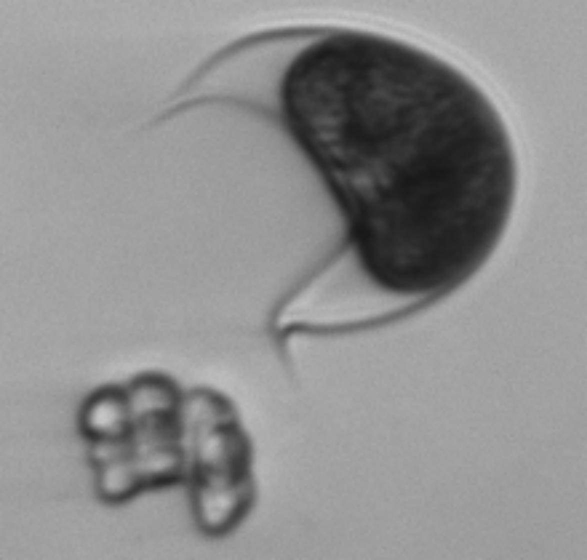

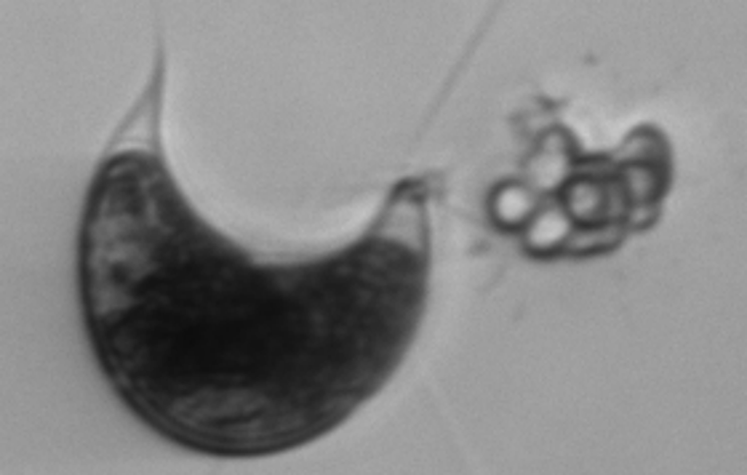

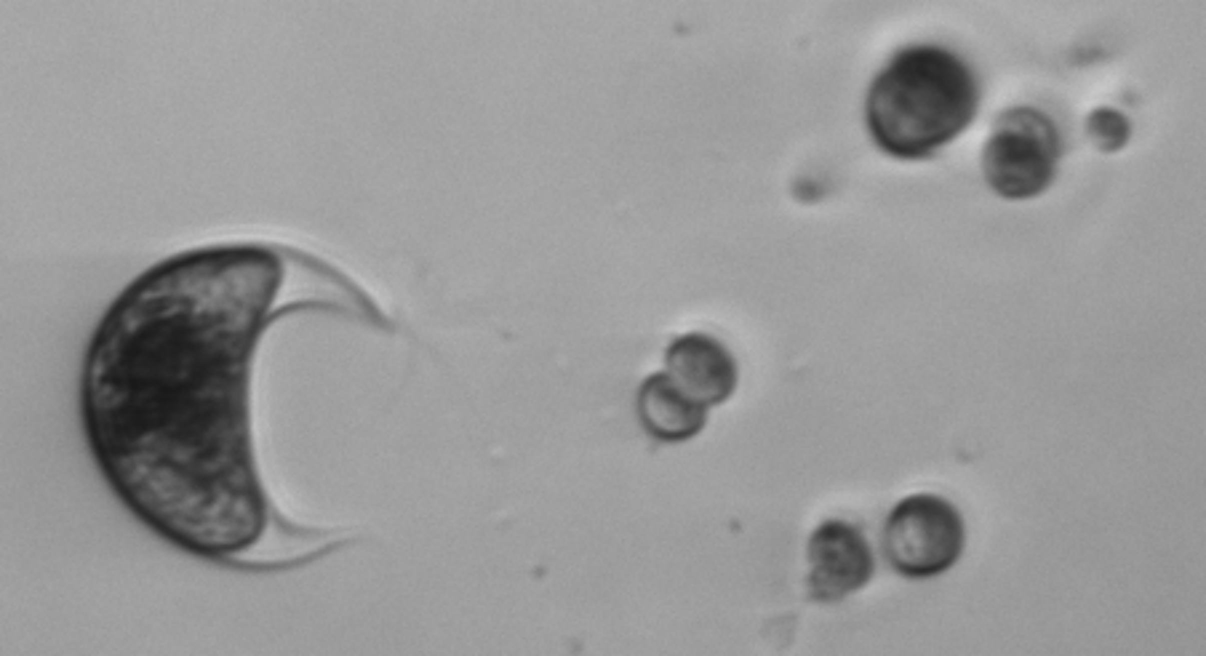

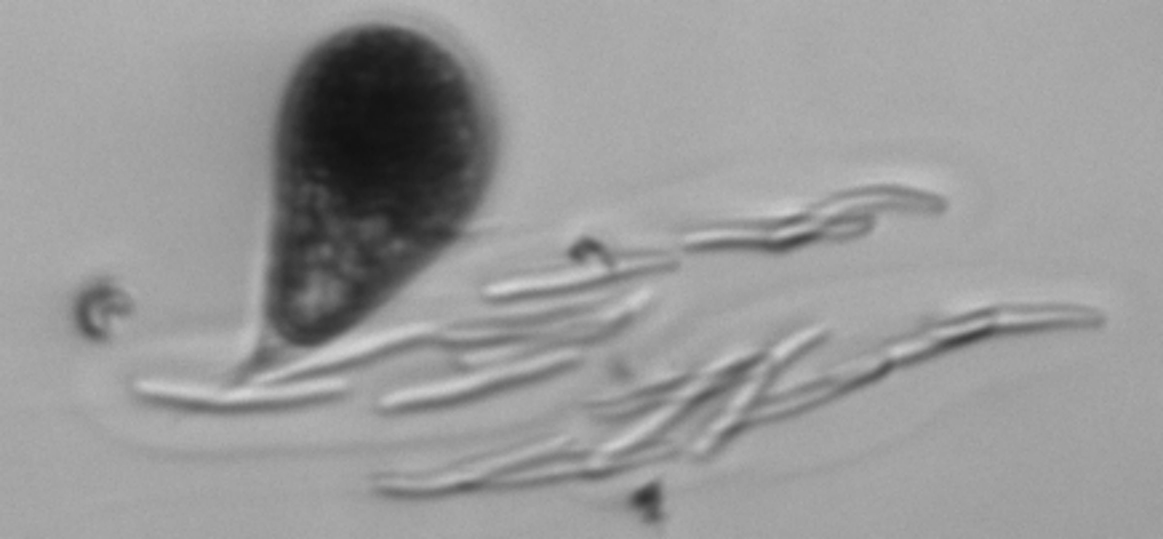

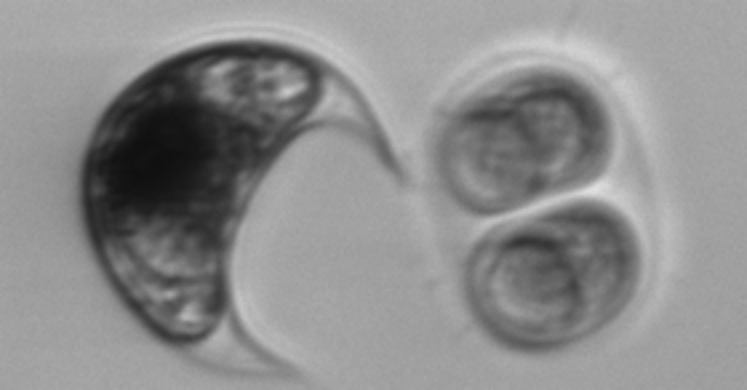

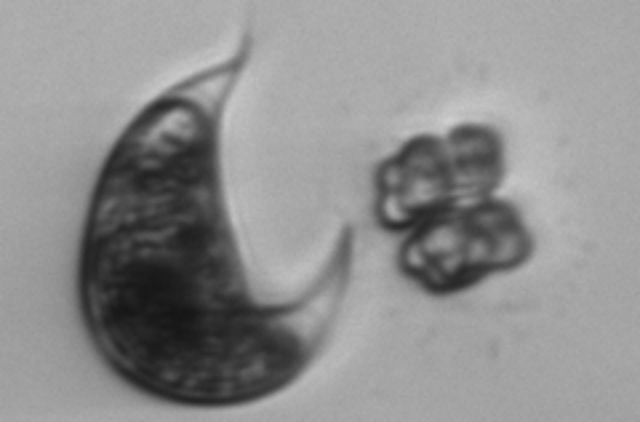

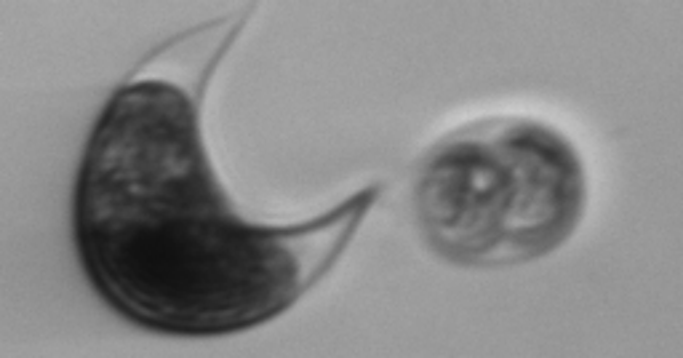

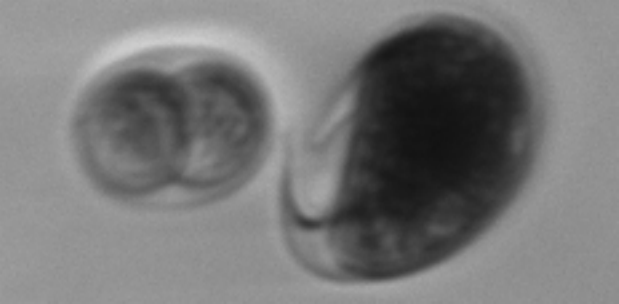

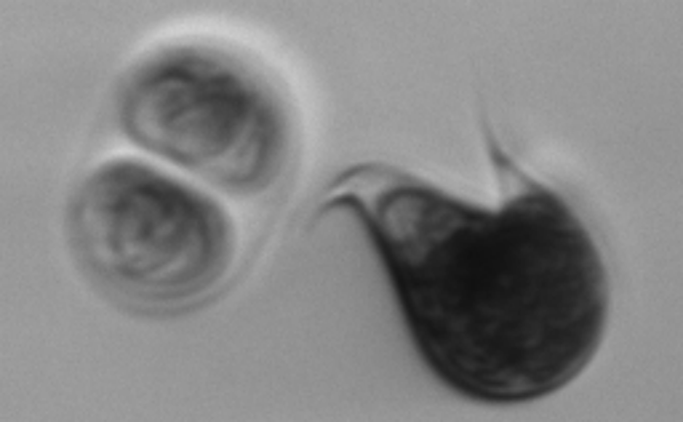

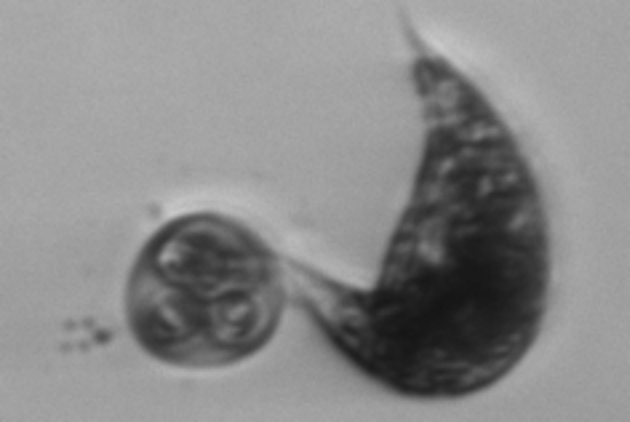

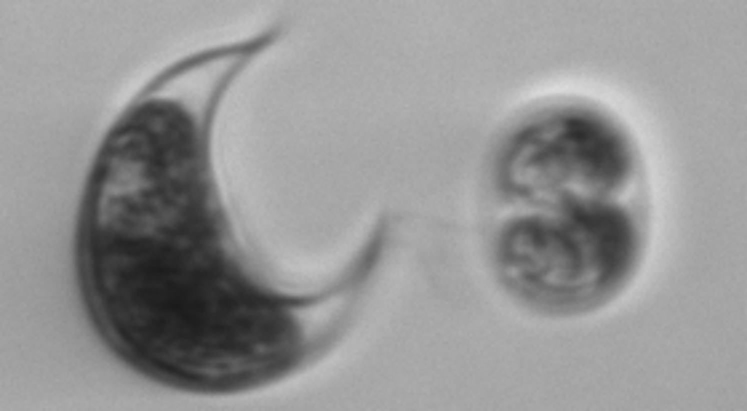

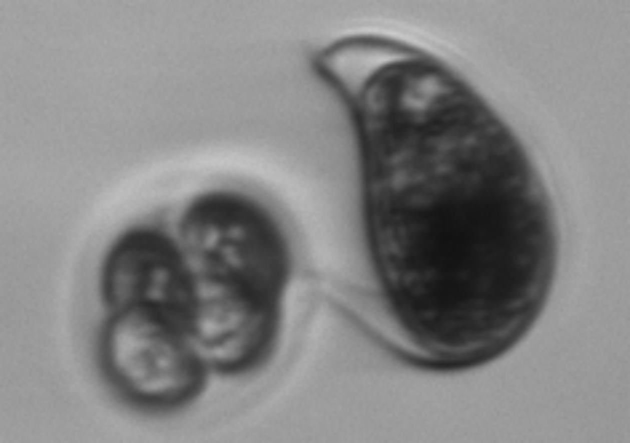

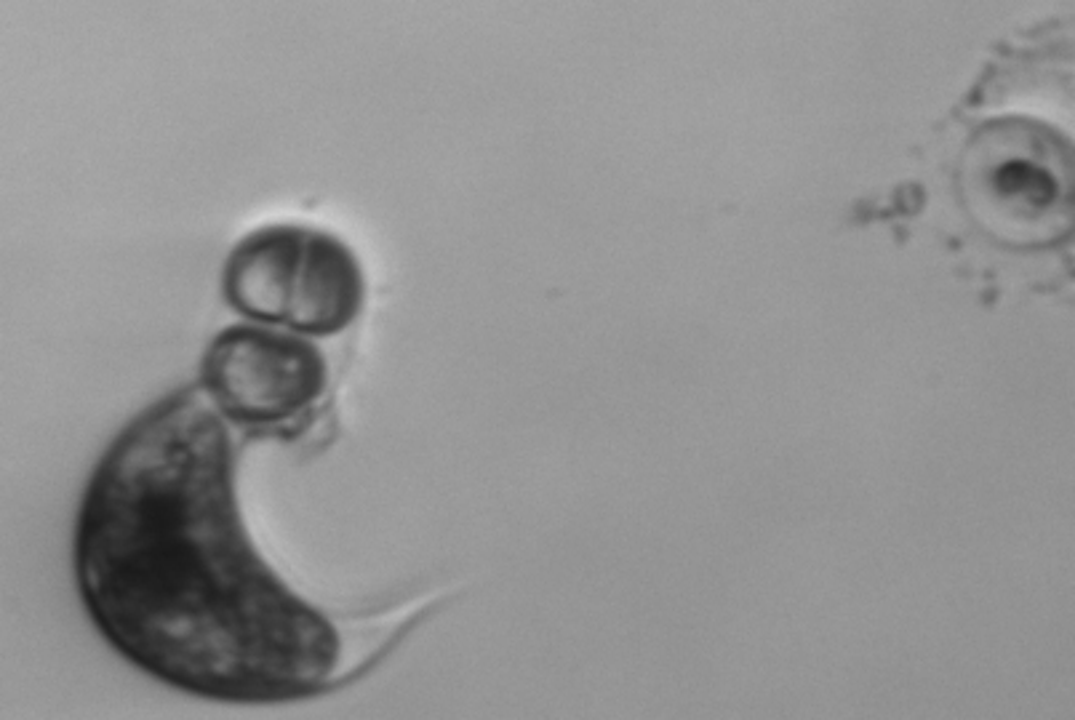

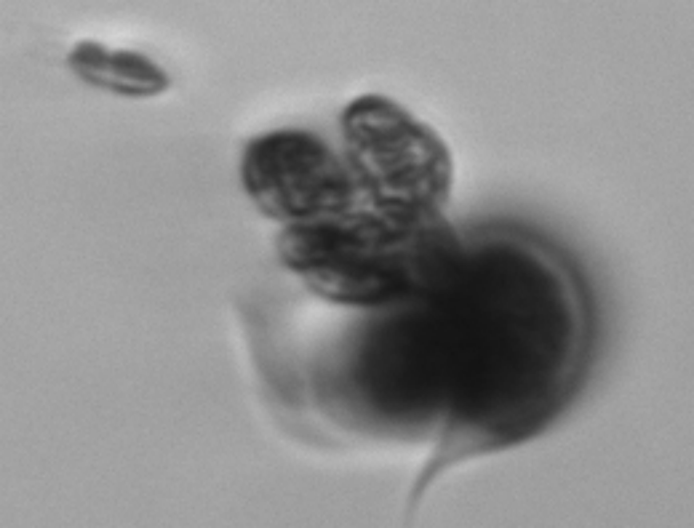

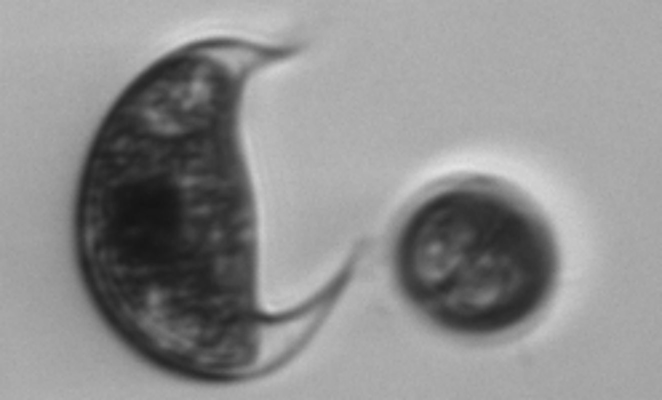

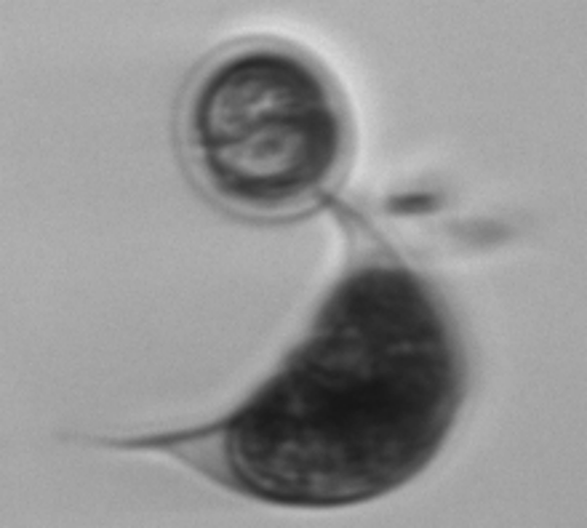

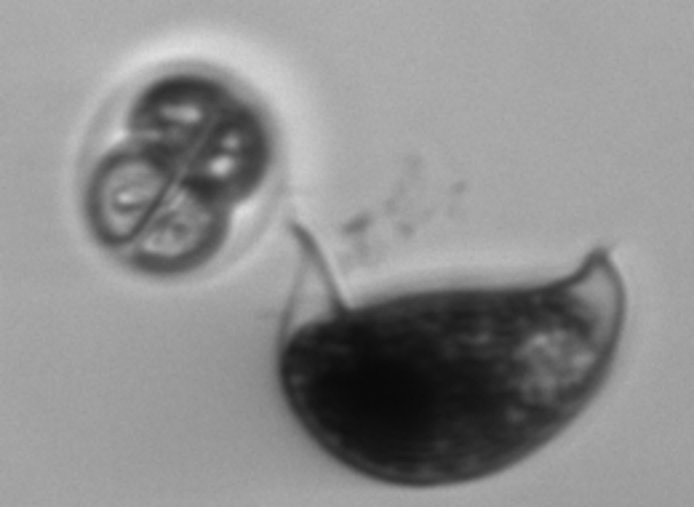

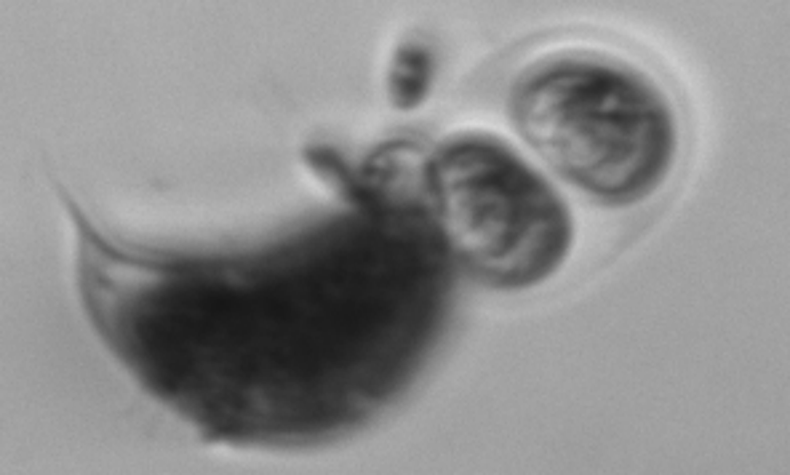

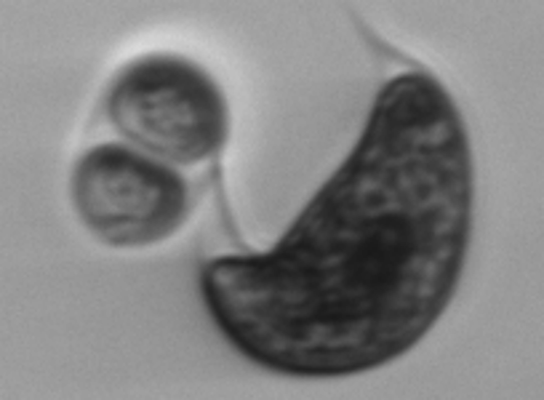

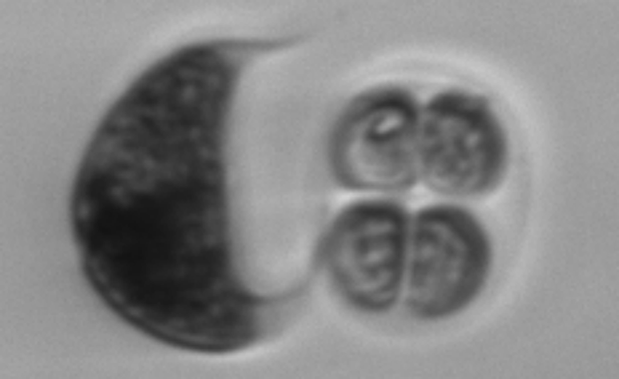

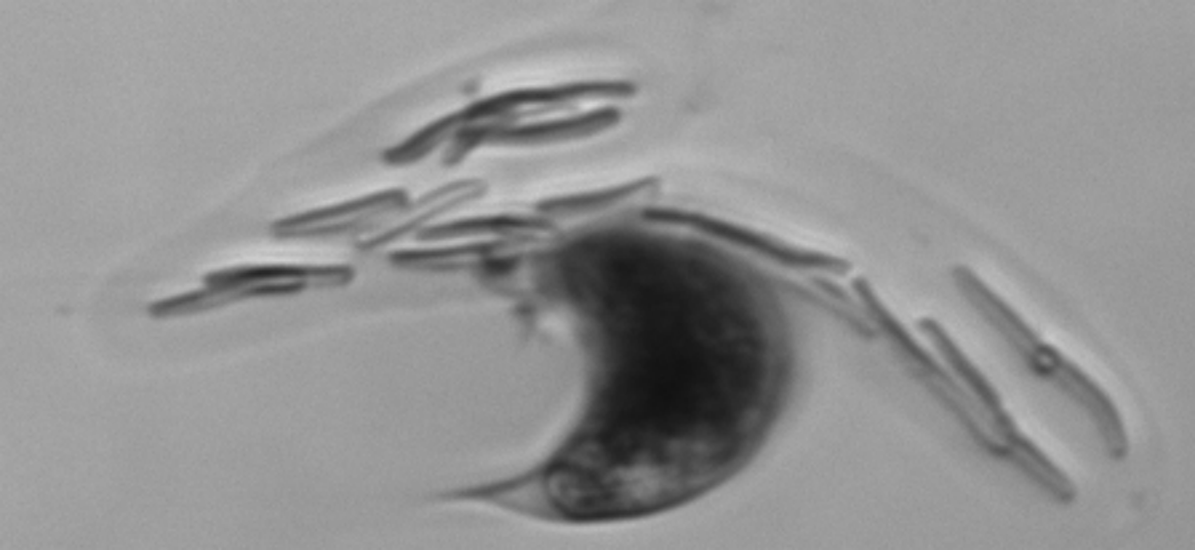

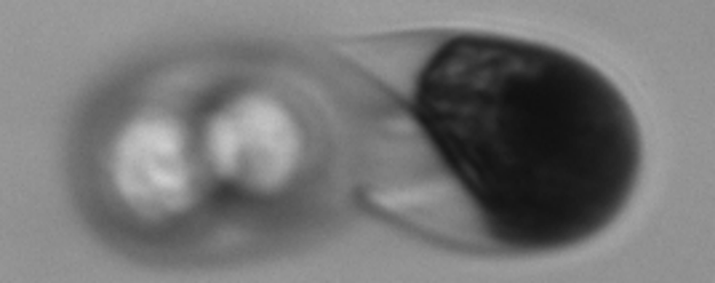

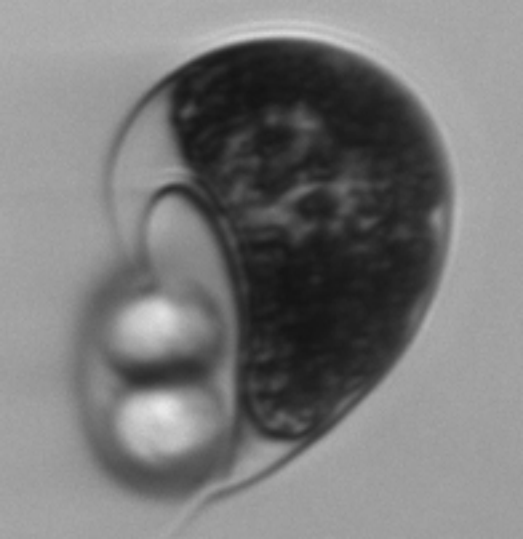

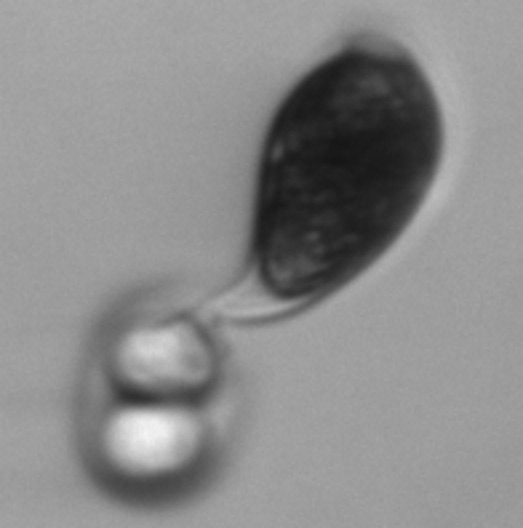

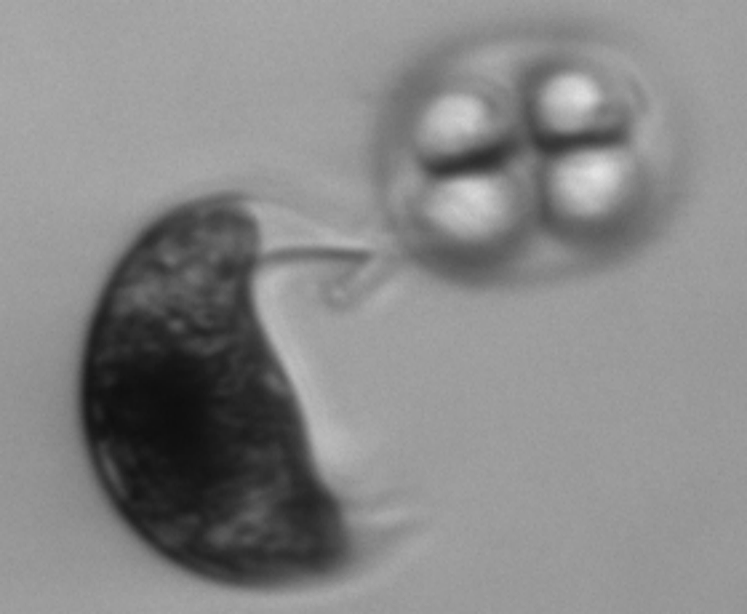

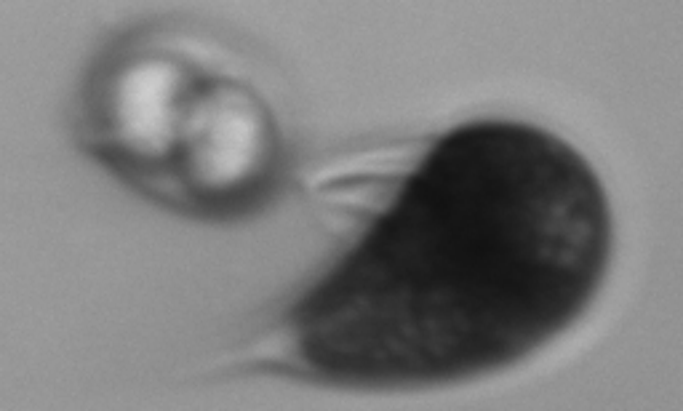

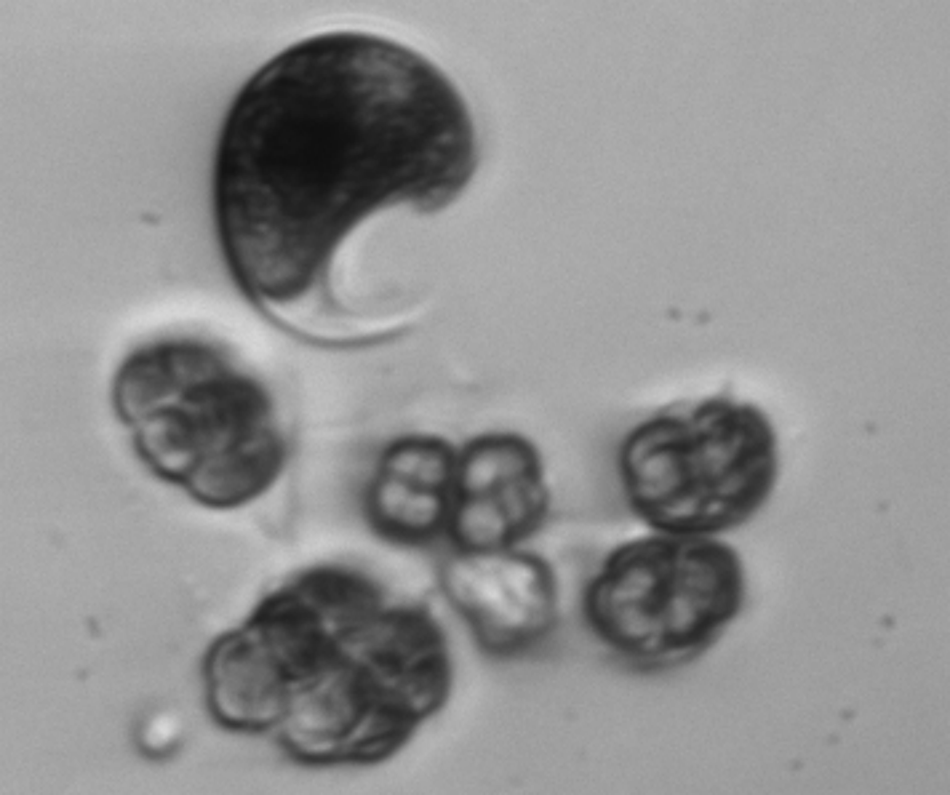

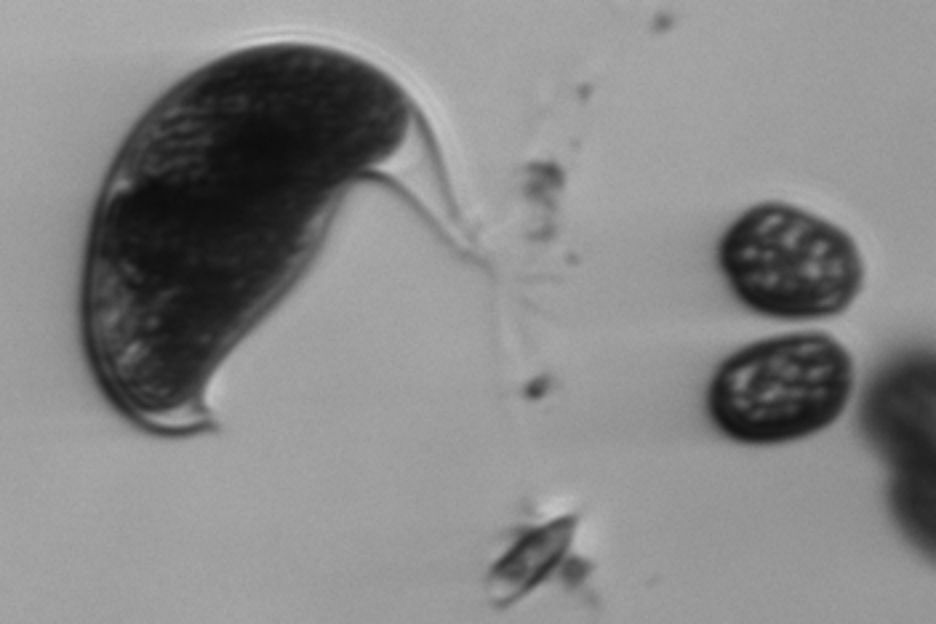

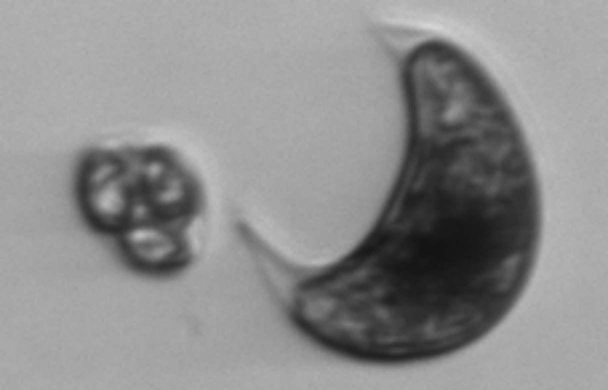

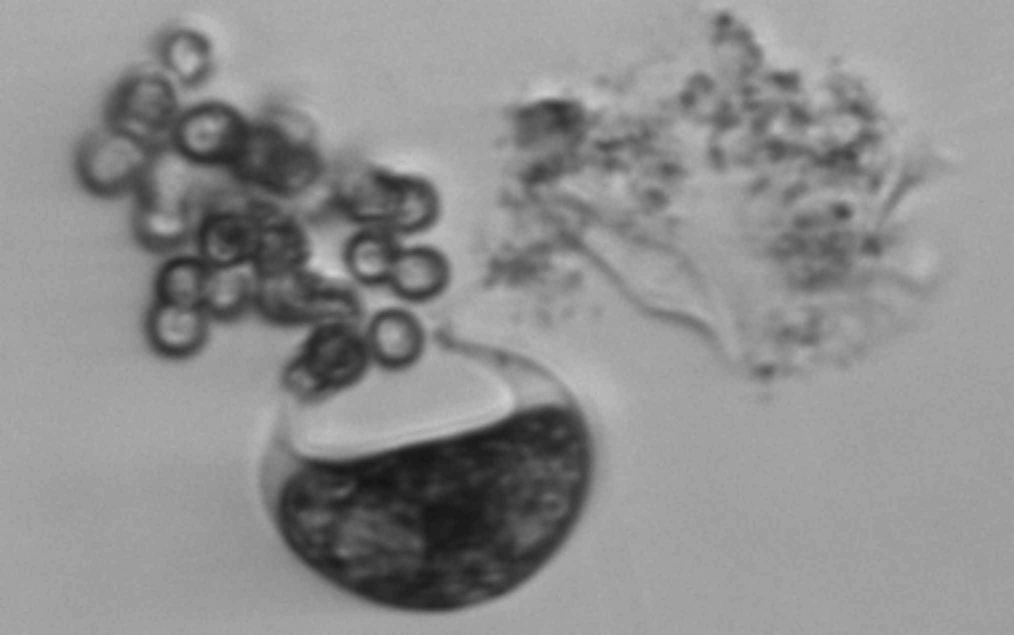

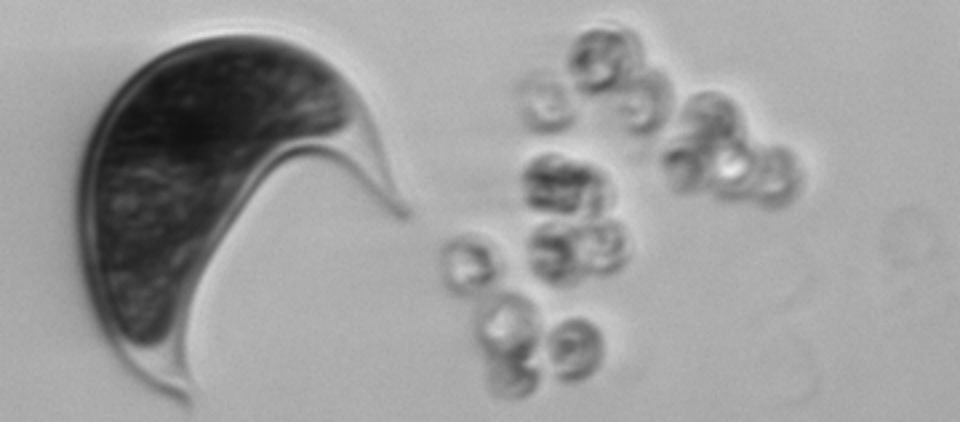

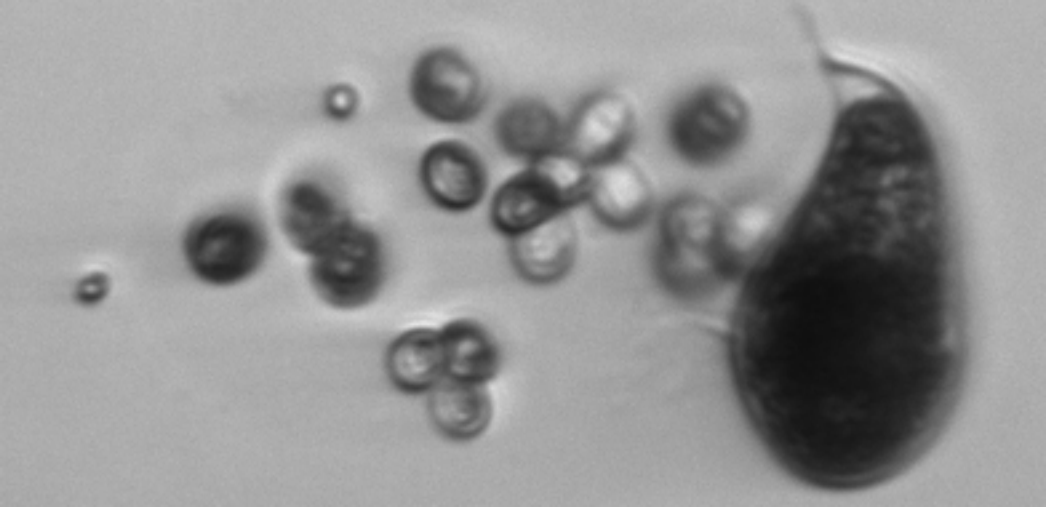

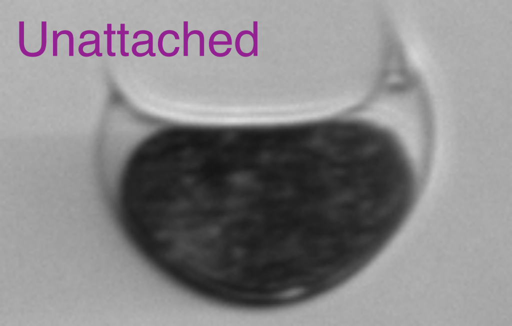
Unattached

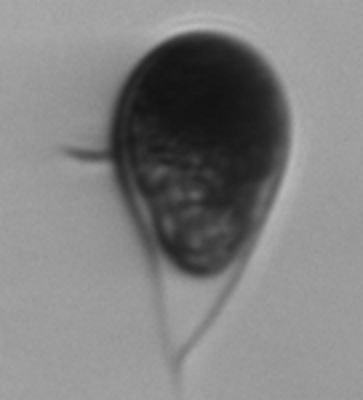

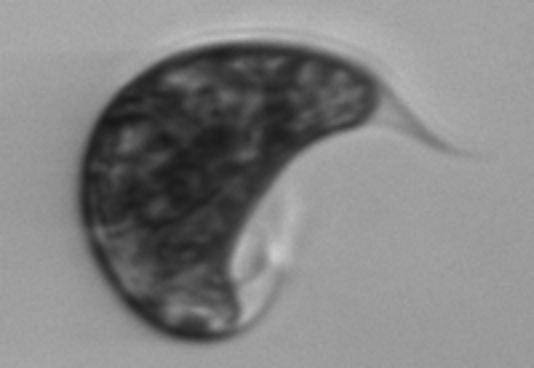

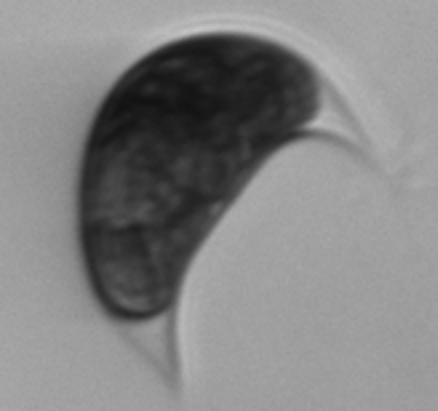

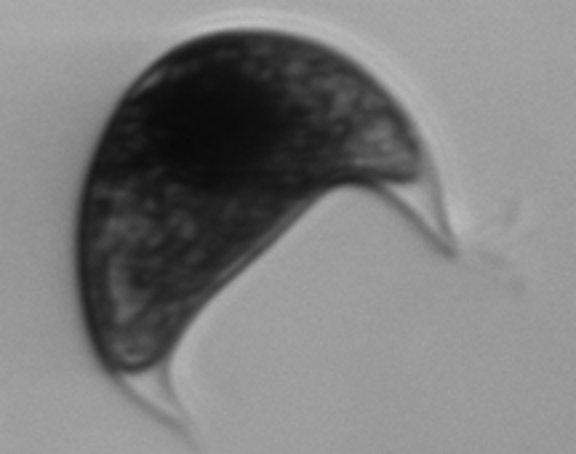

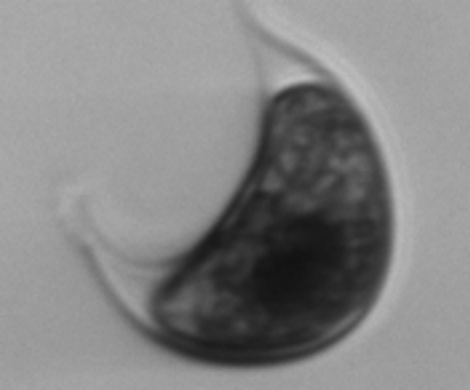

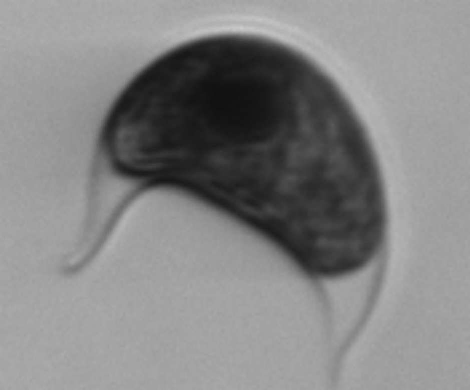

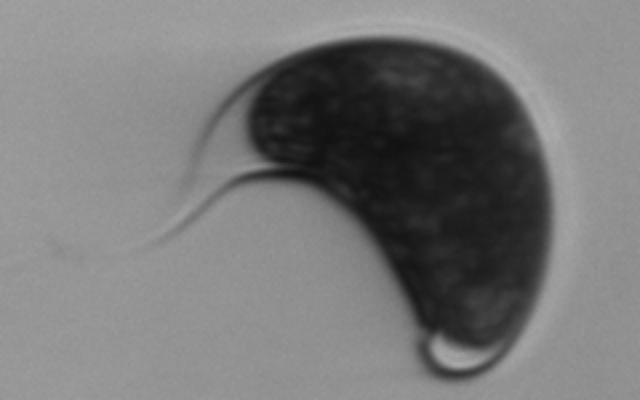

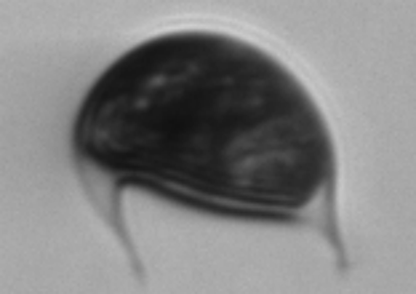

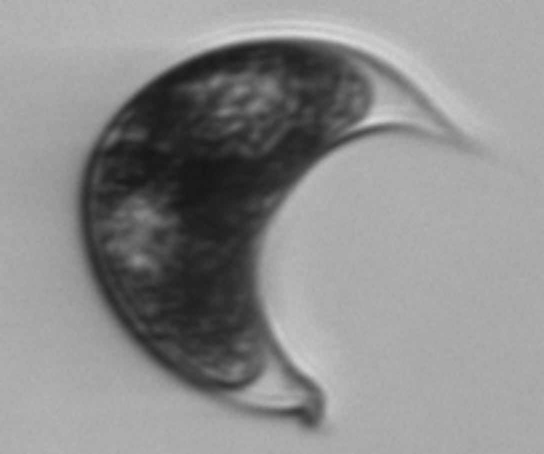

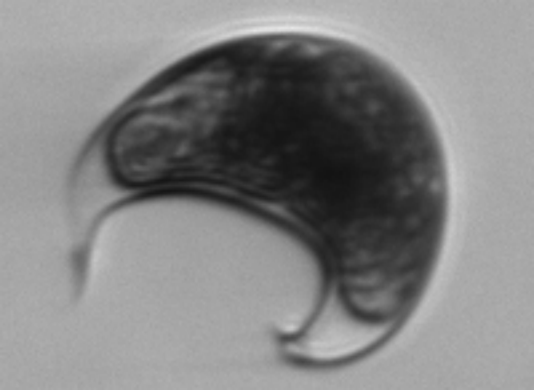

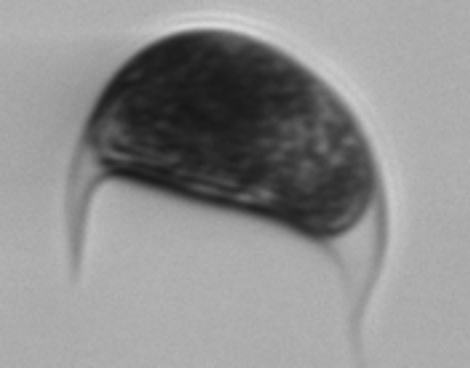

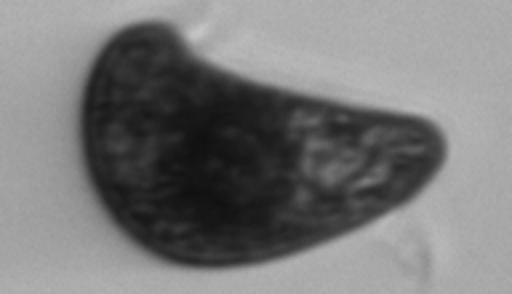

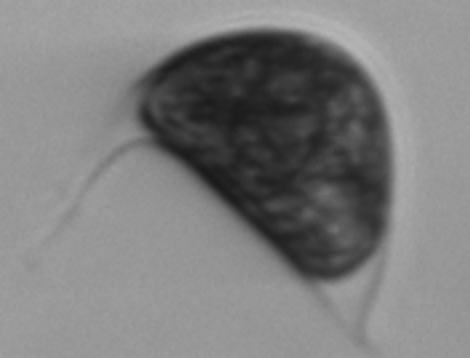

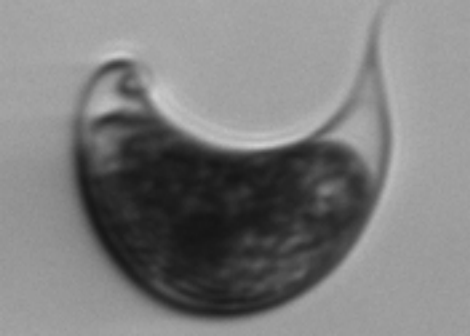

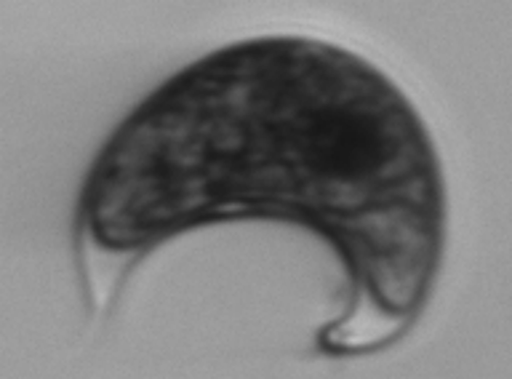

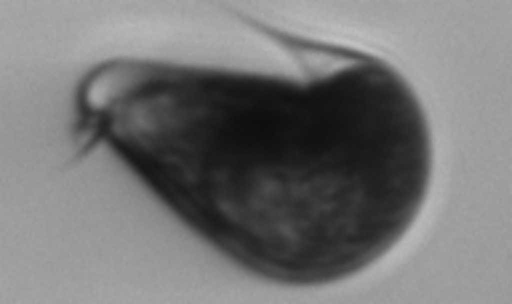

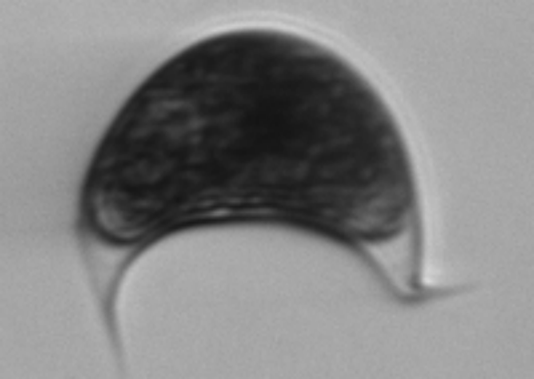

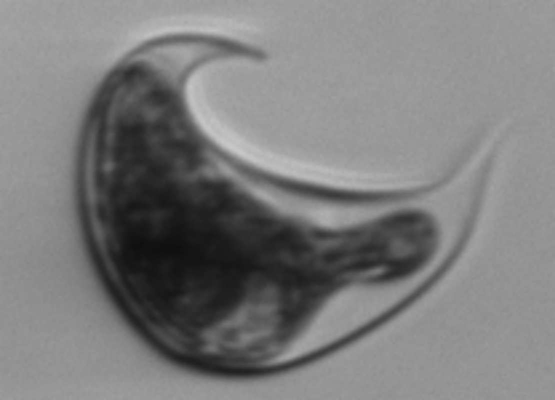

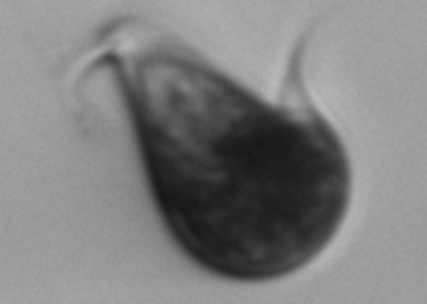

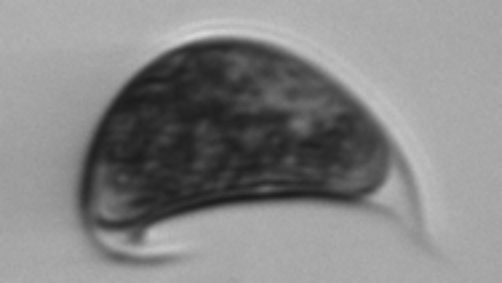

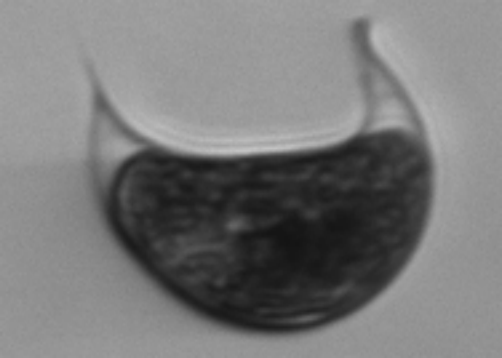

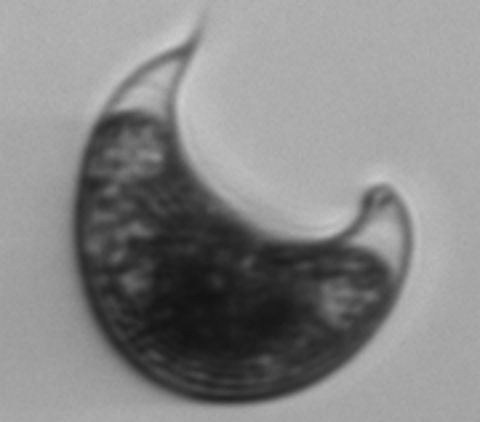

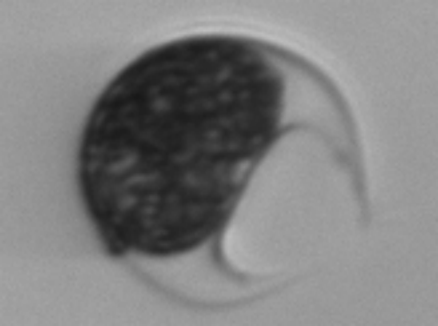

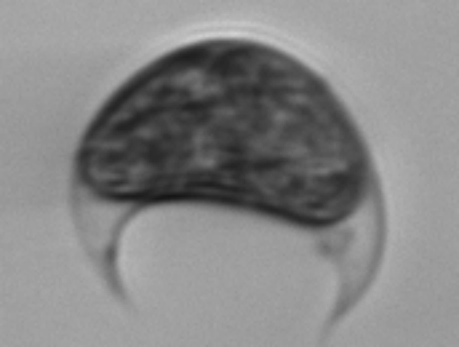

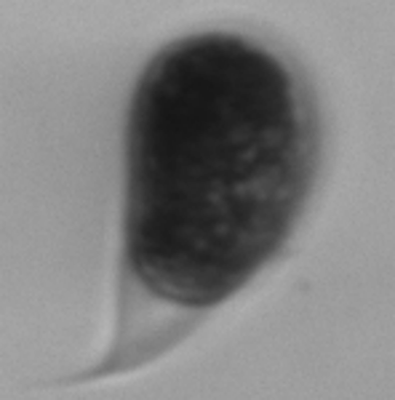

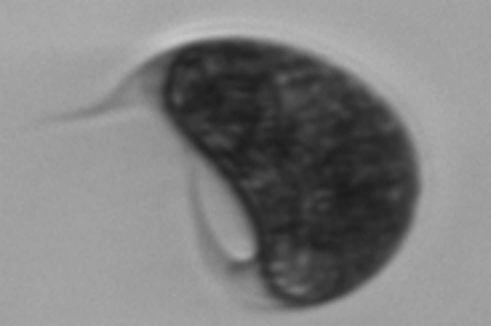

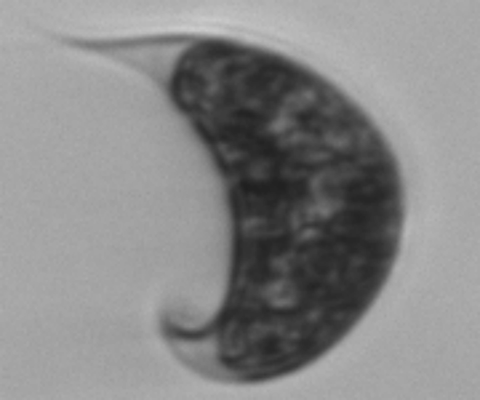

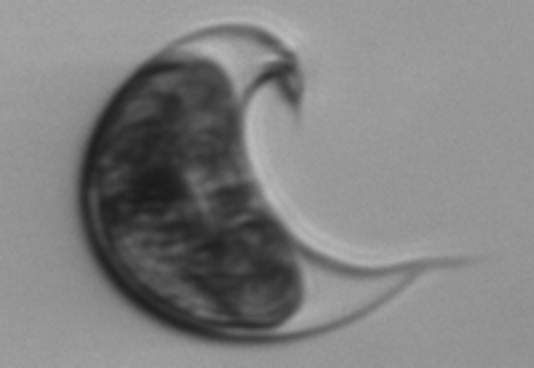

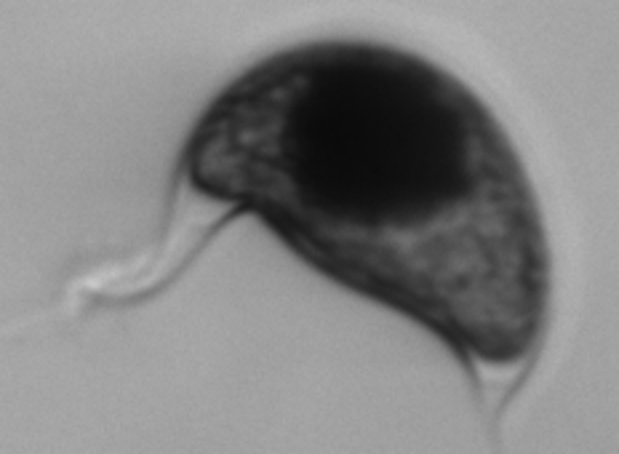

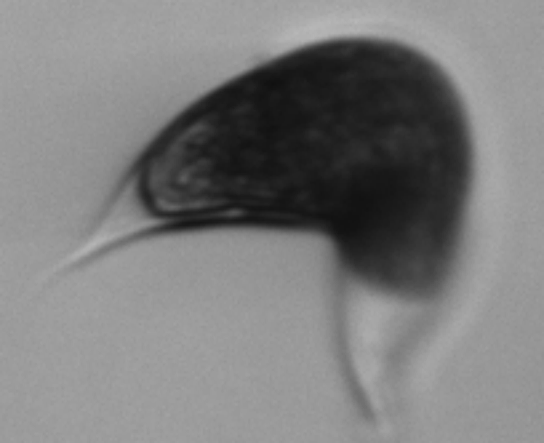

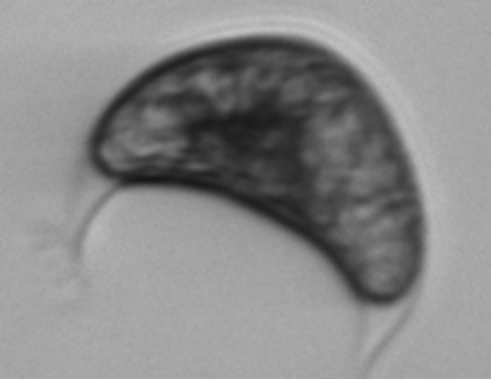

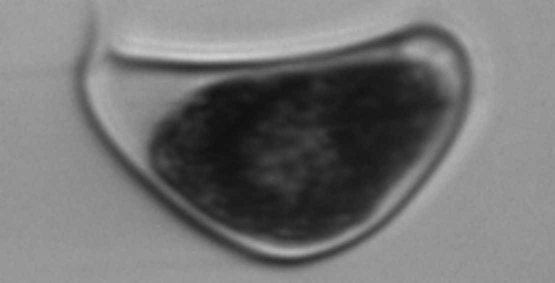

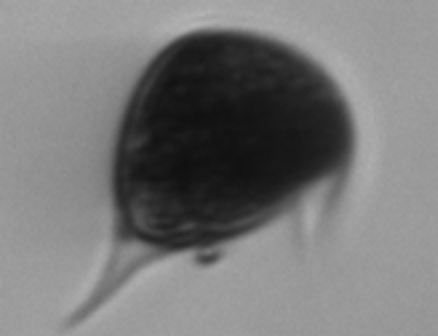

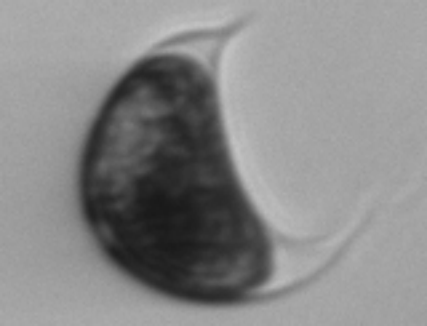

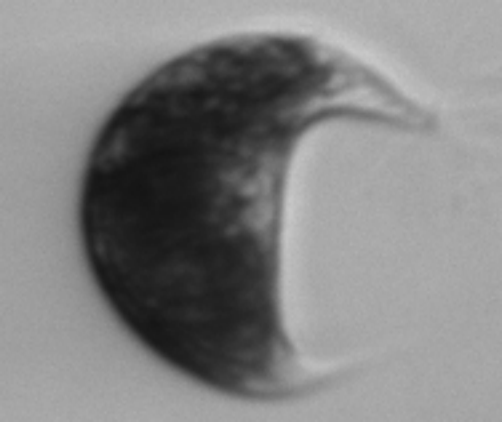

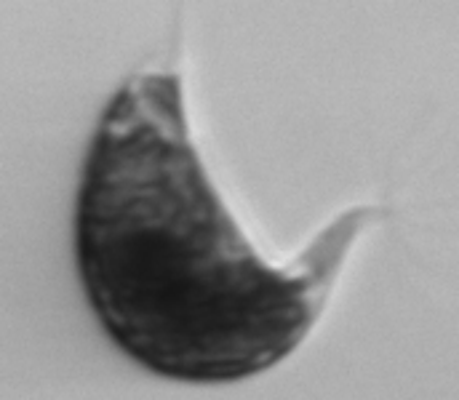

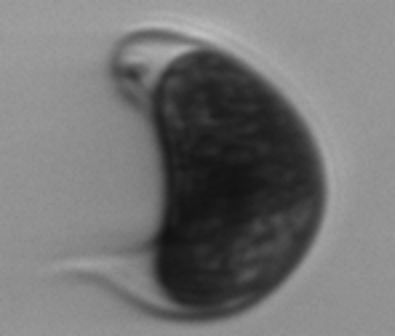

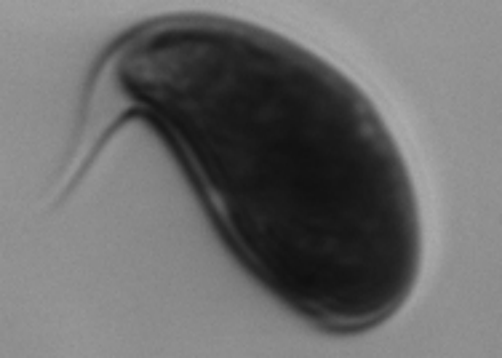

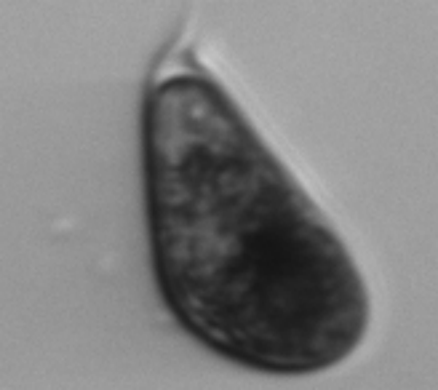

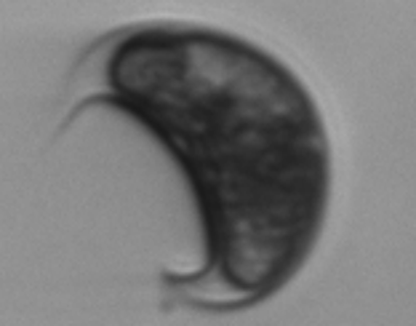

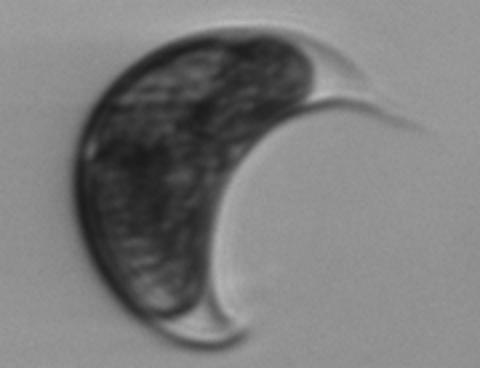

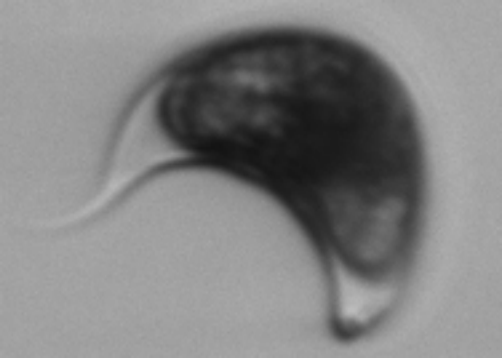

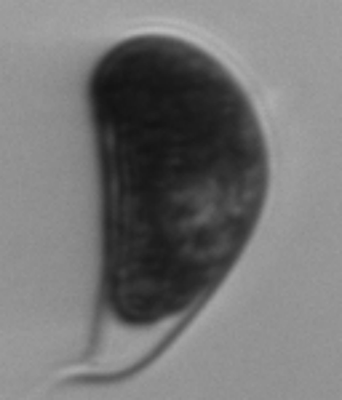

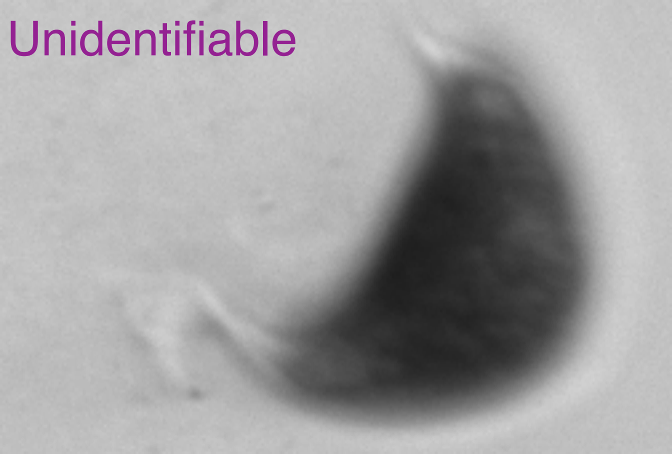
Unidentifiable

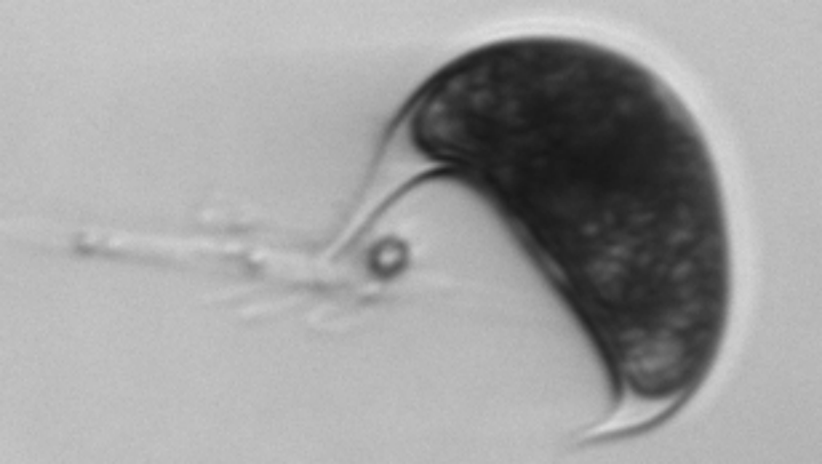

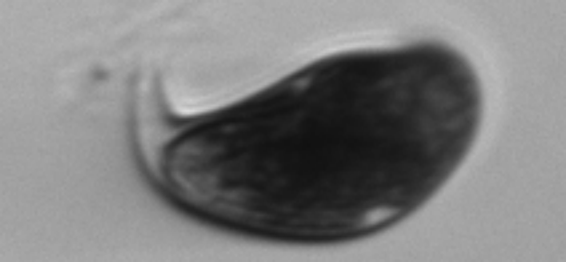

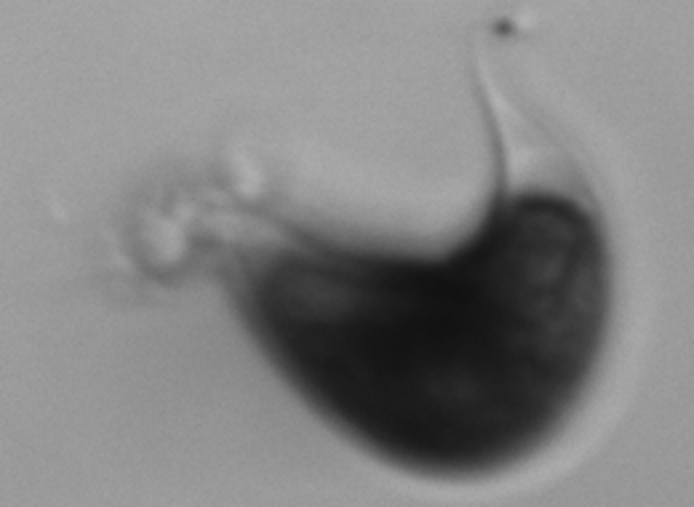

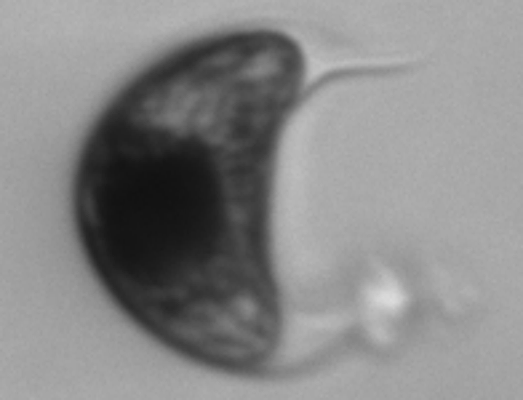

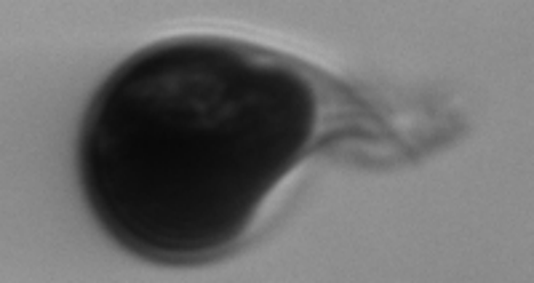

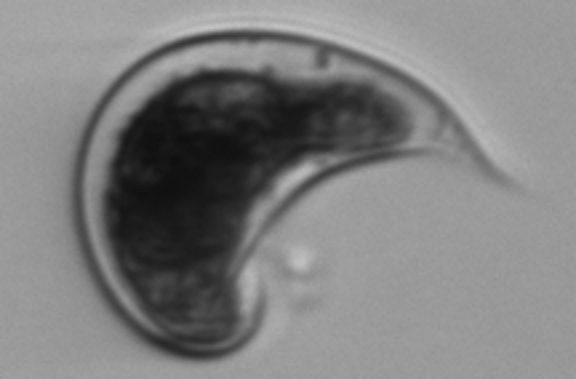

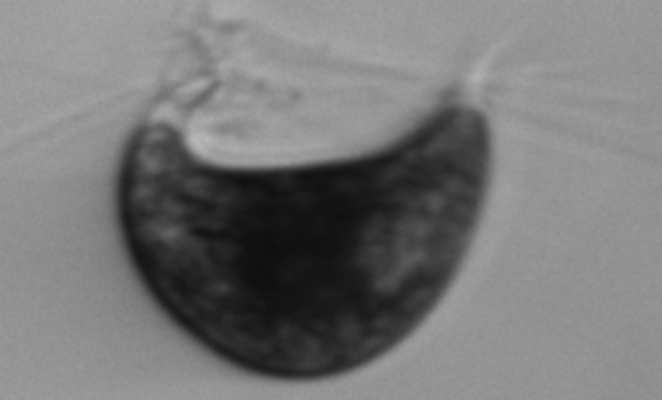

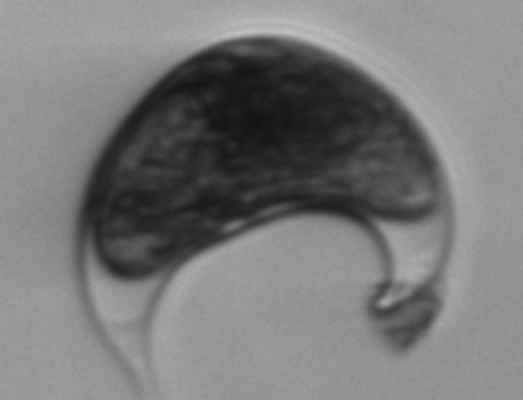

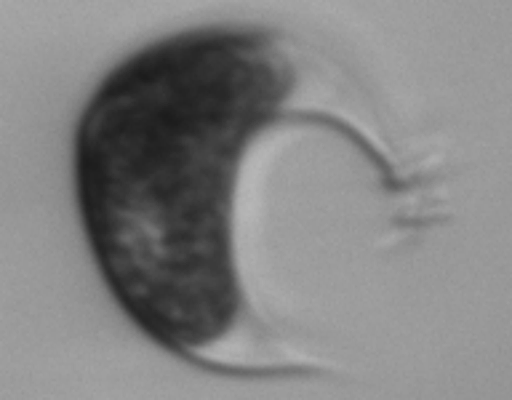

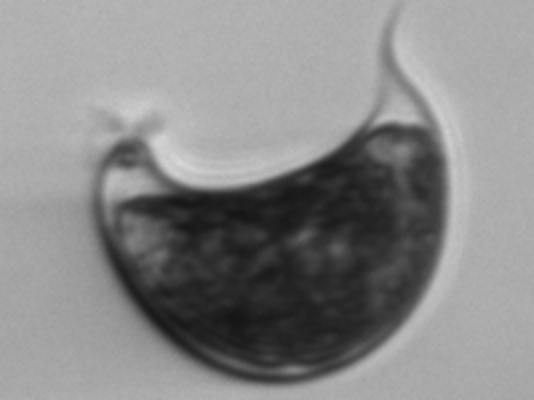

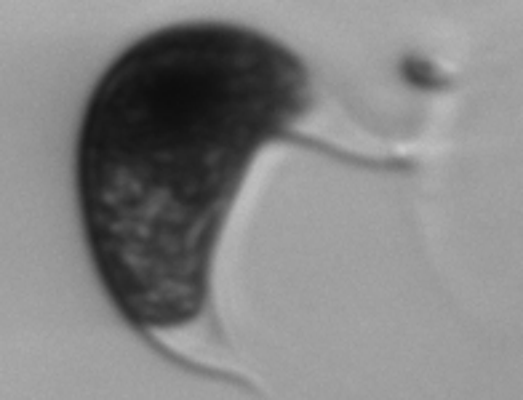

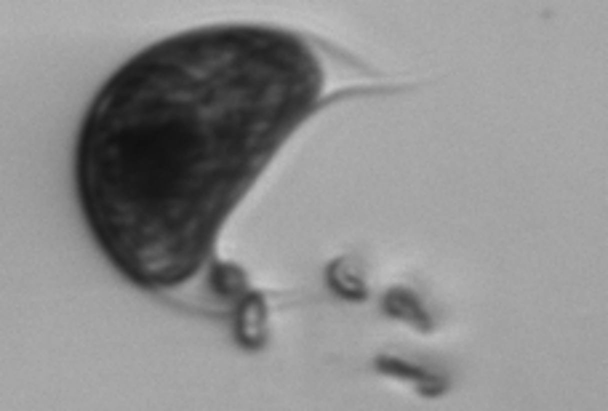

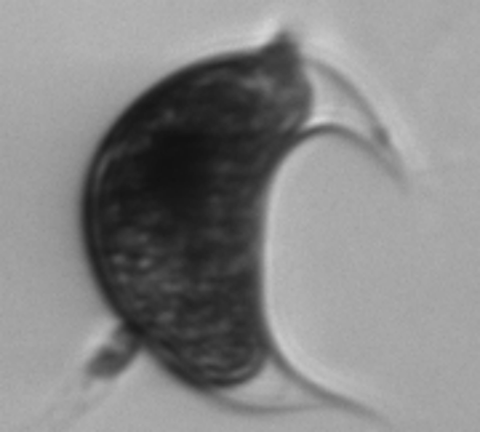

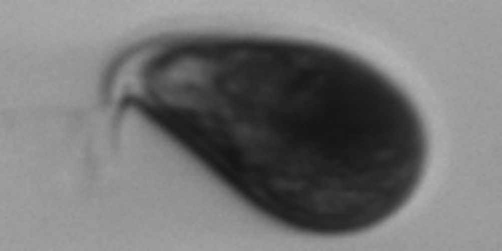

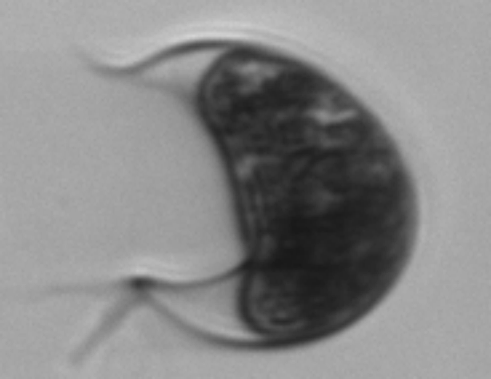

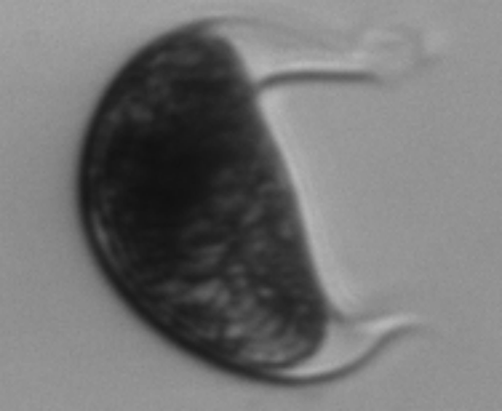

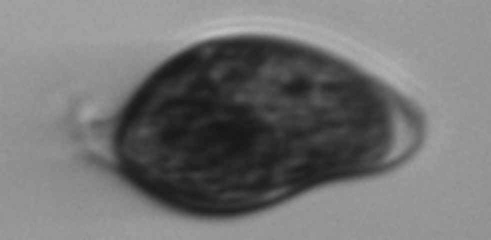

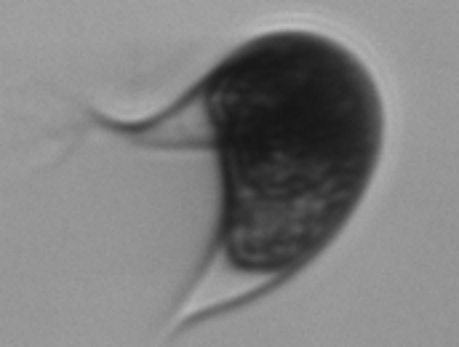

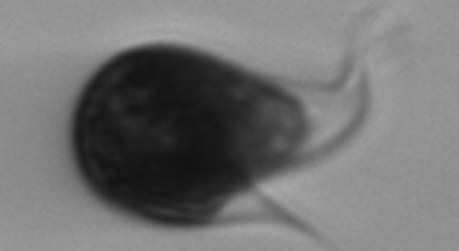

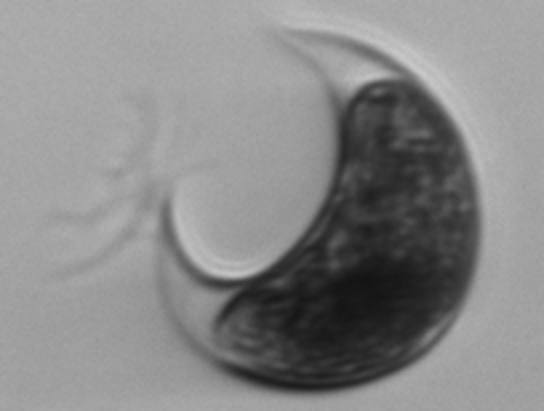

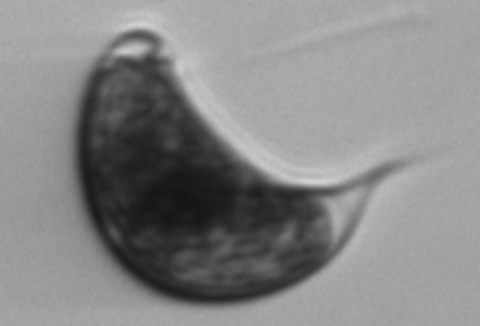

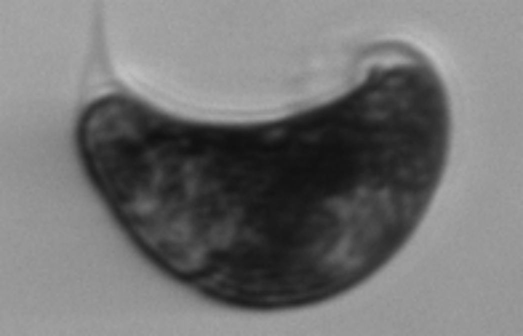

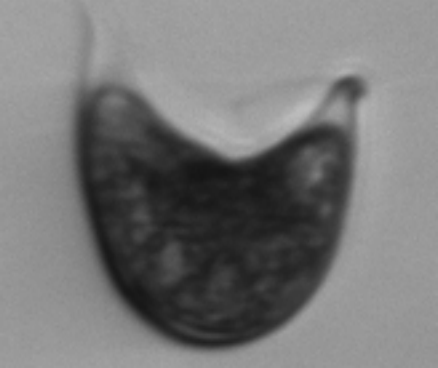

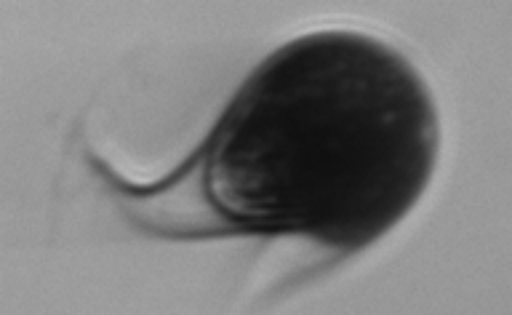

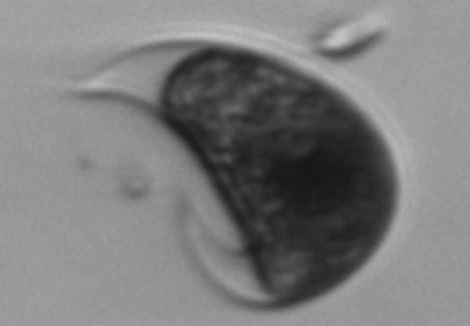

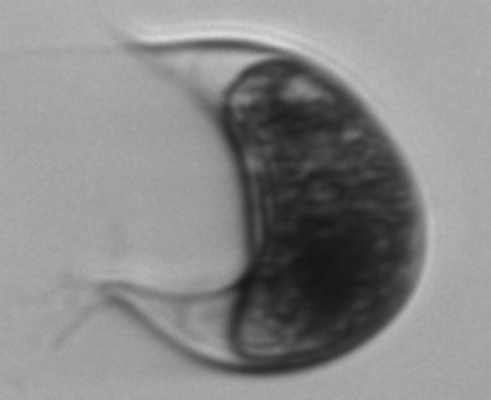

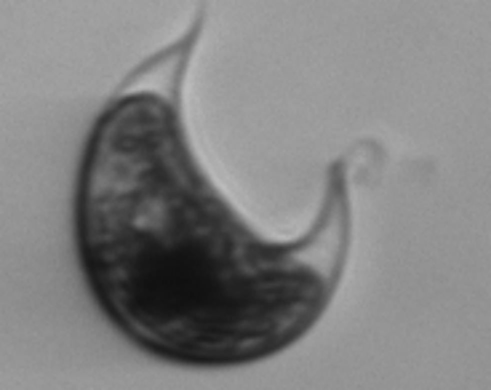

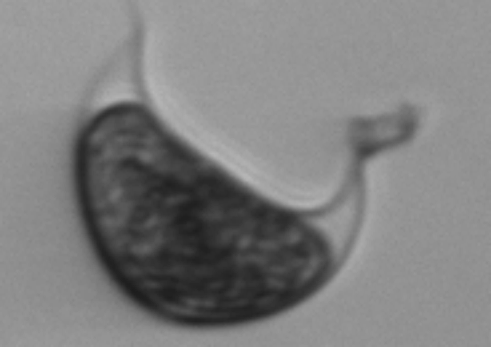

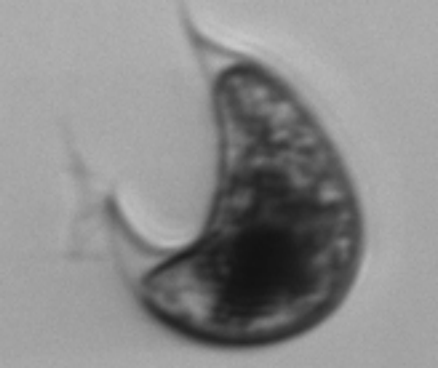

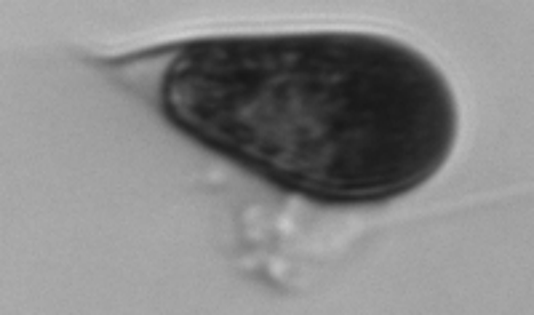

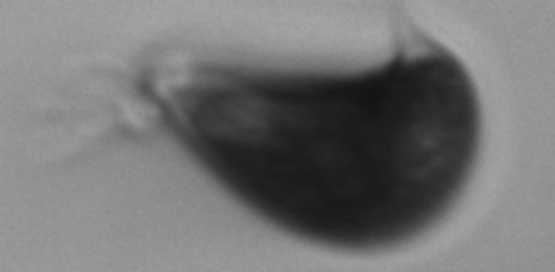

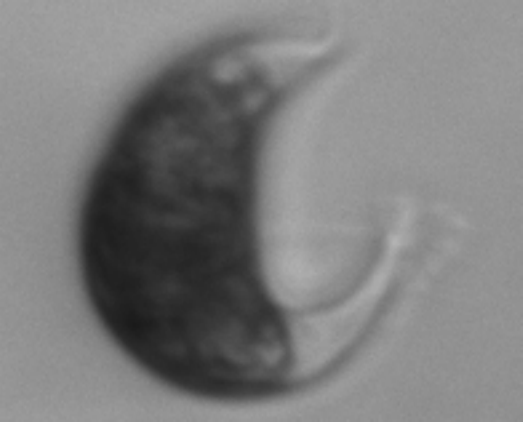

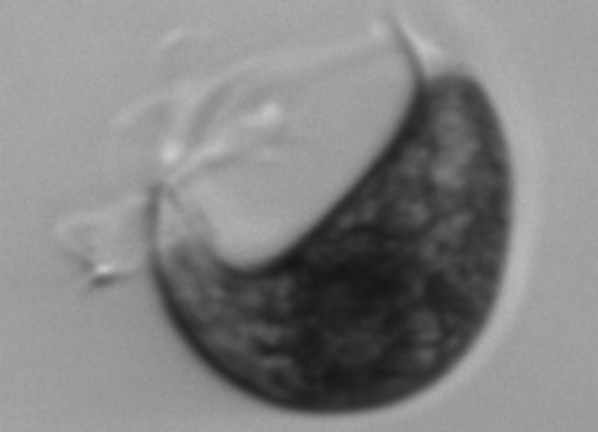

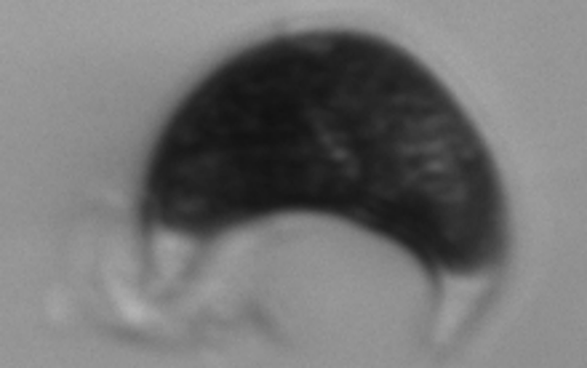

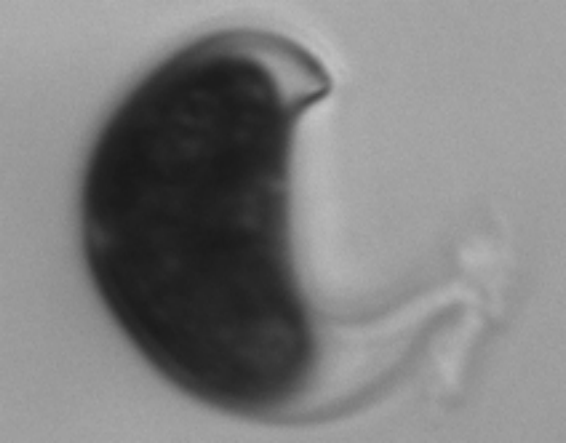

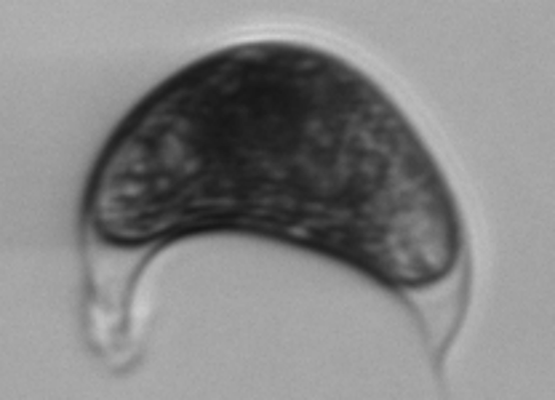

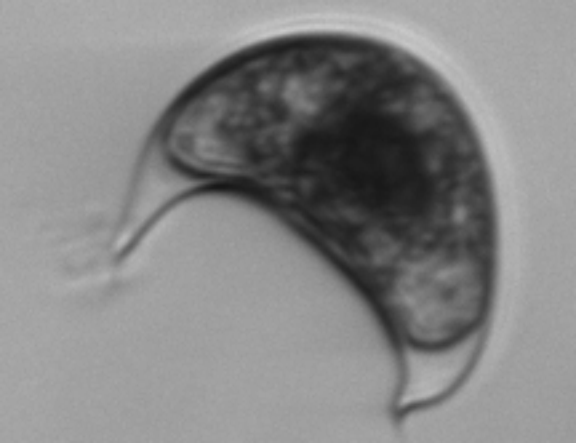

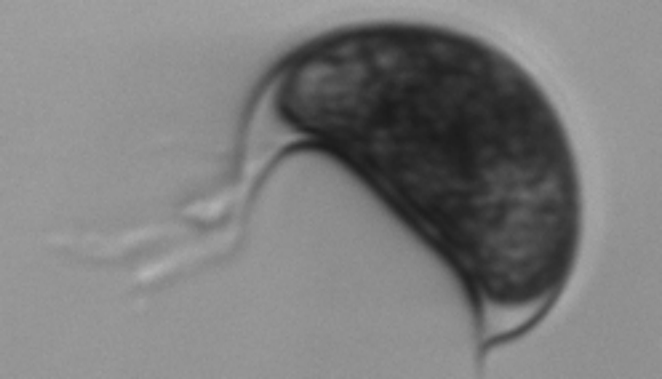

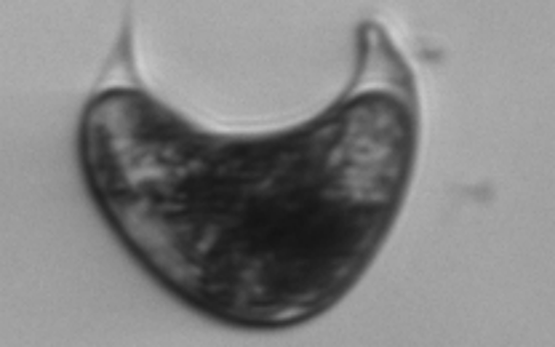

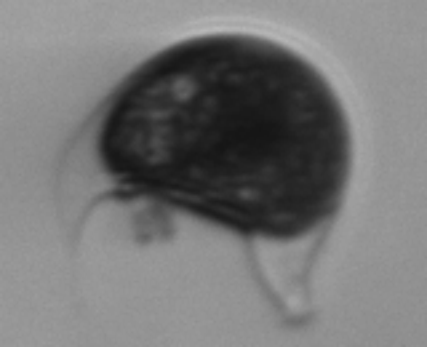

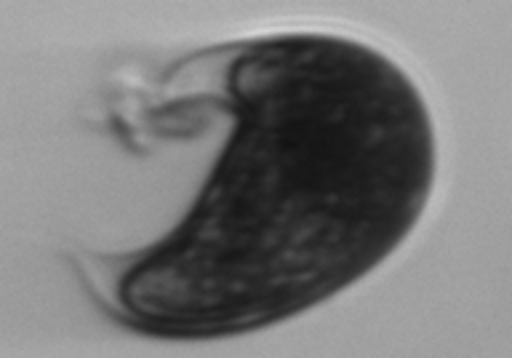

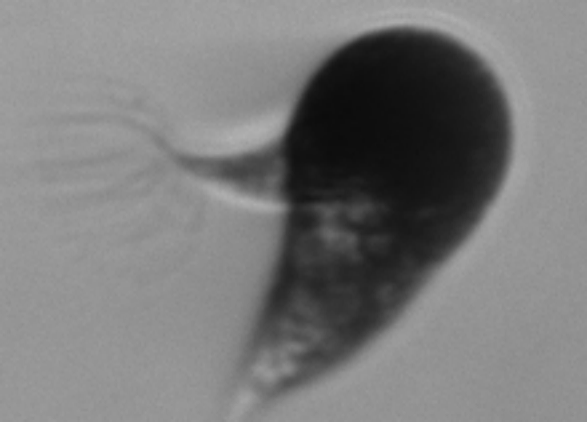

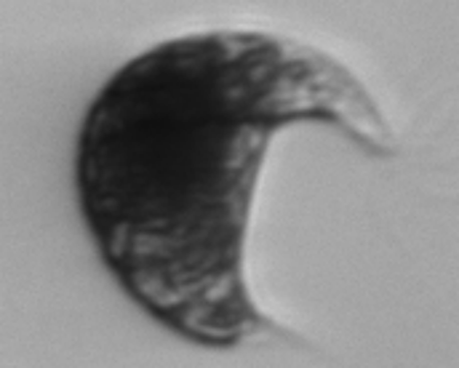

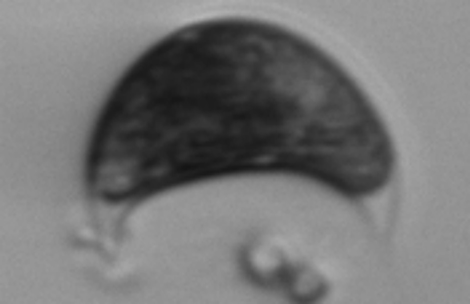

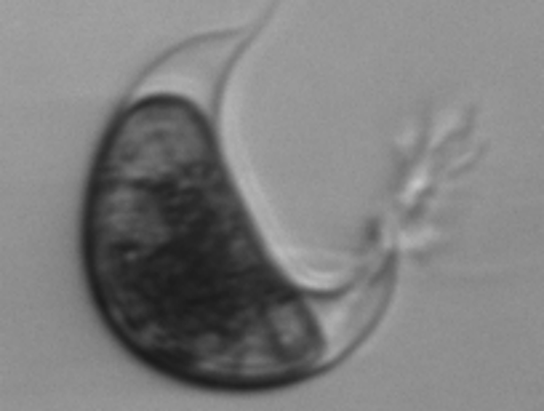

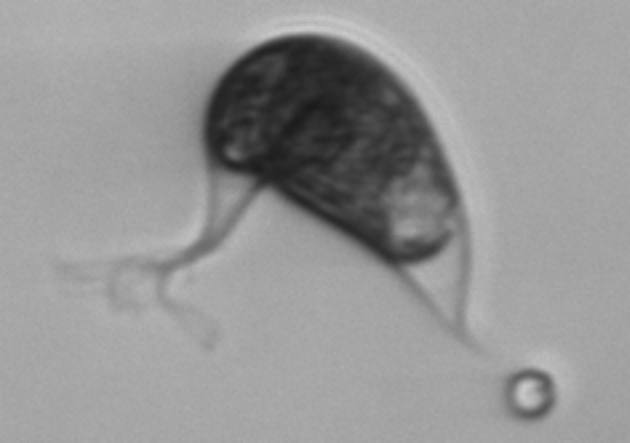

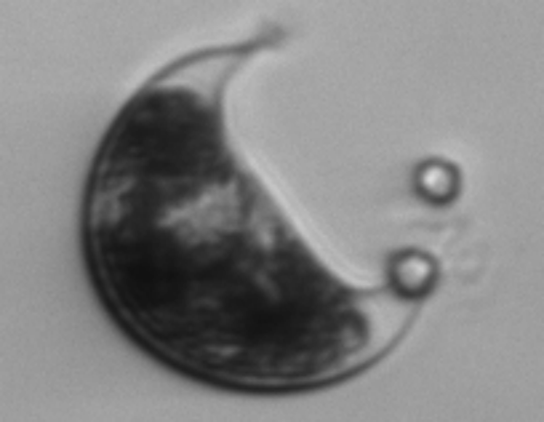

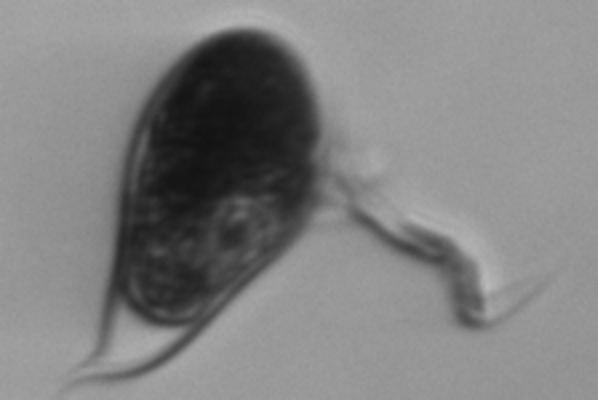

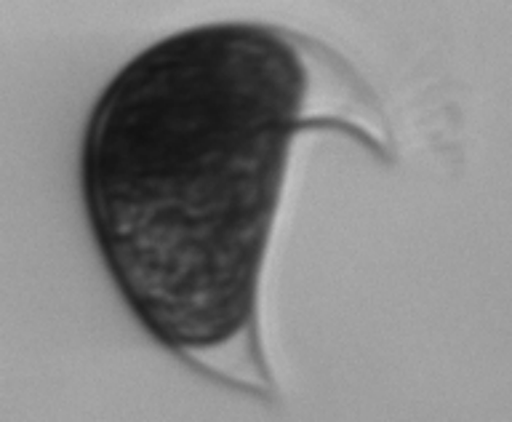

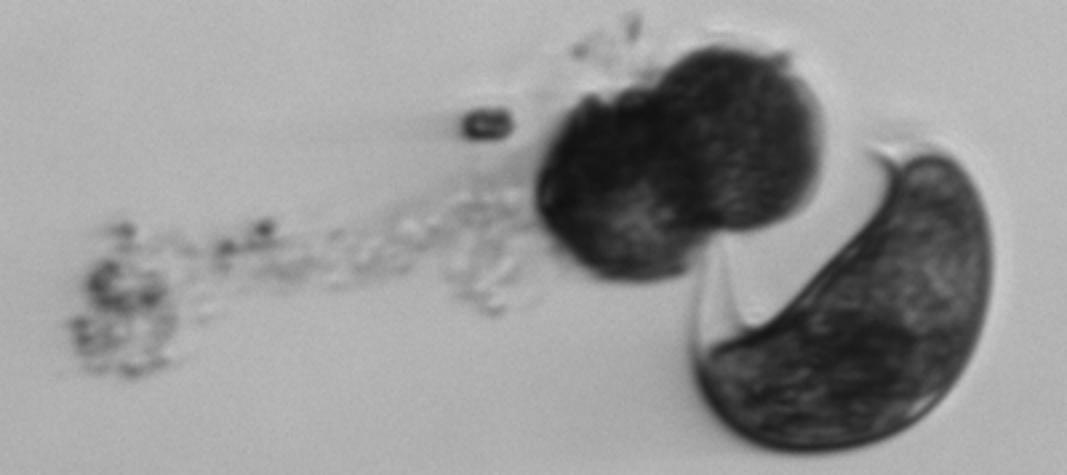

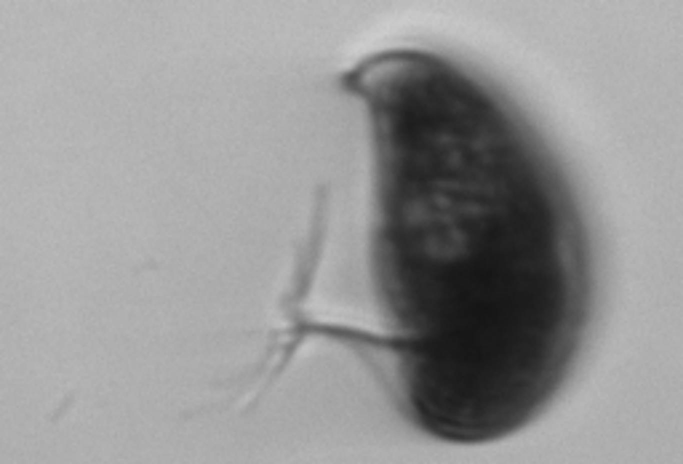

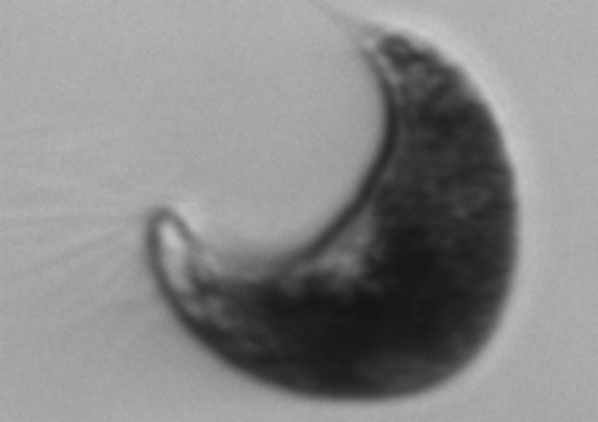

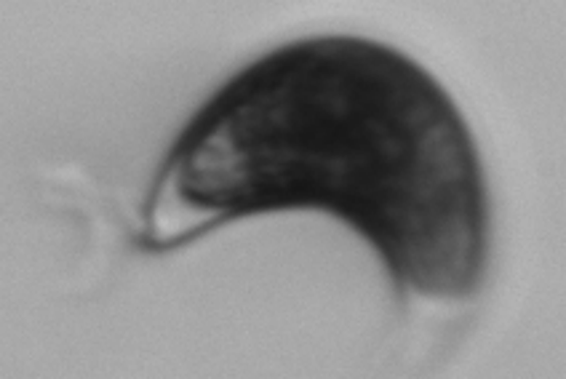

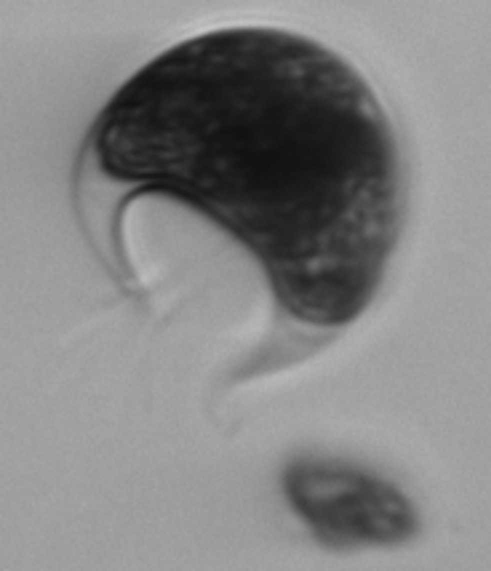

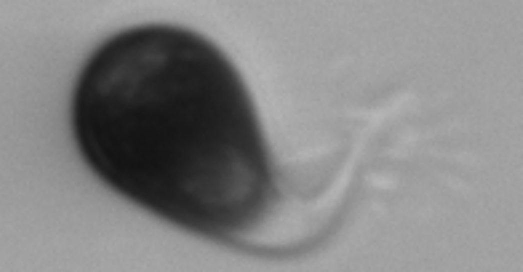

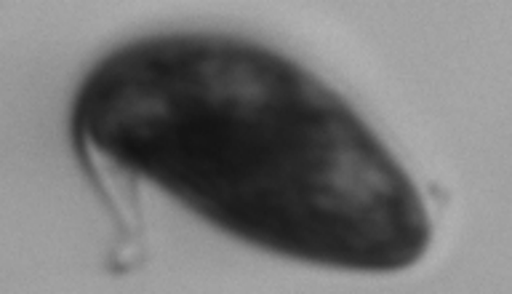

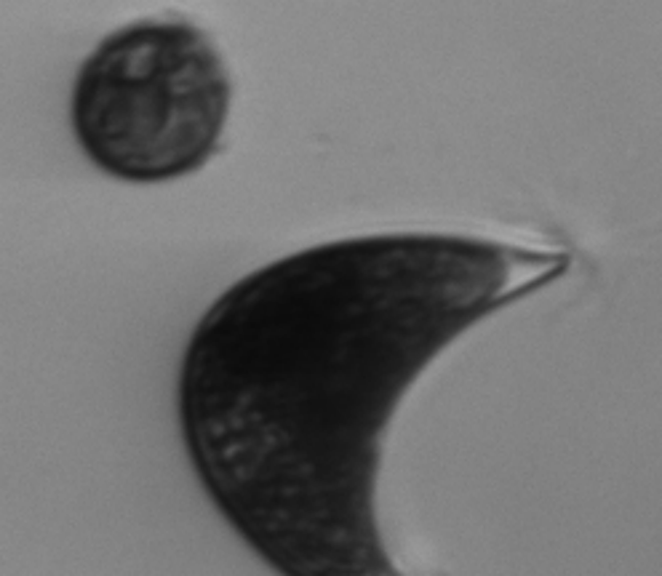

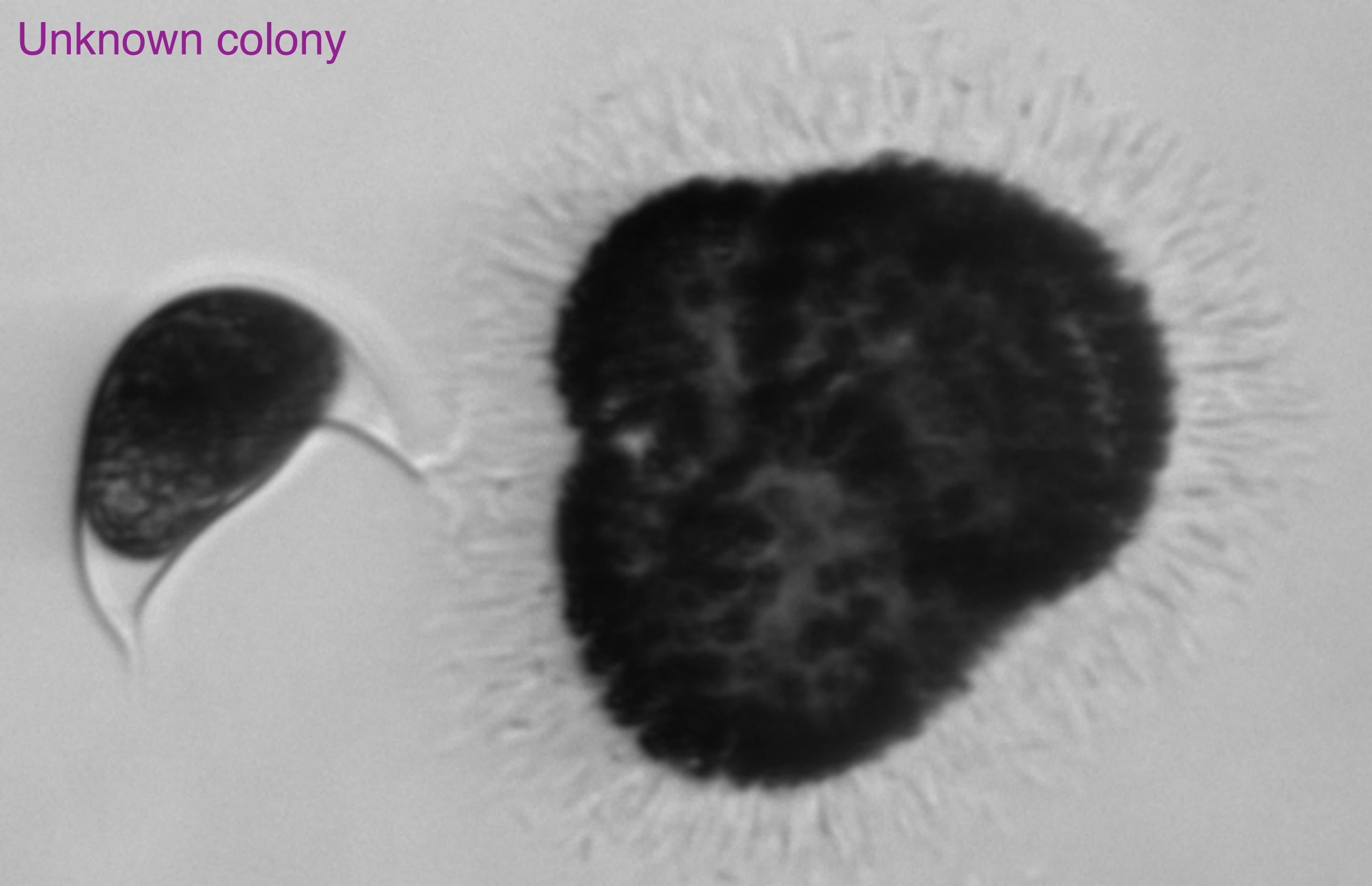
Unknown colony

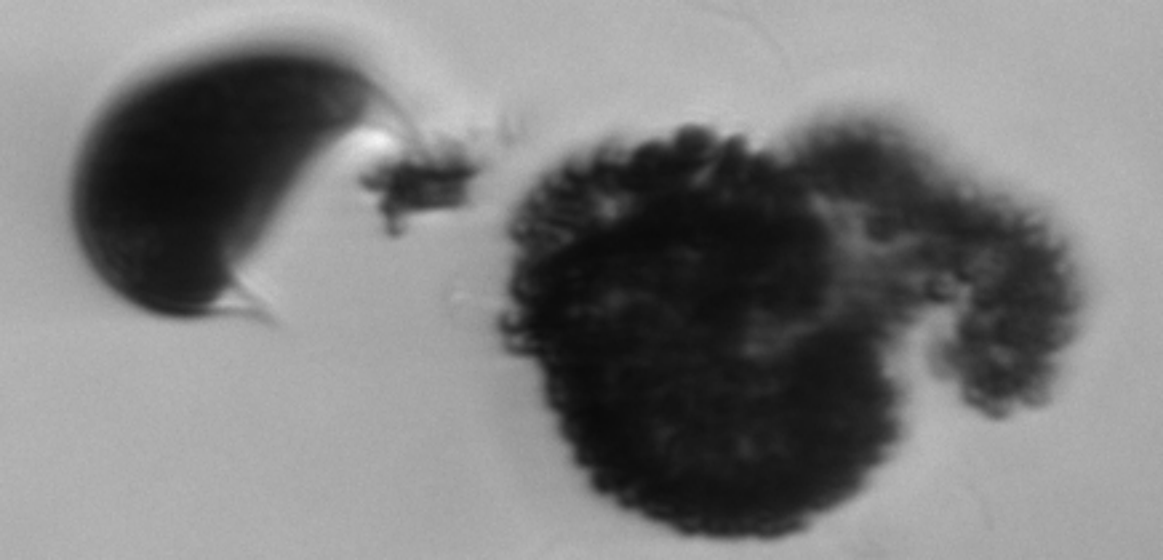

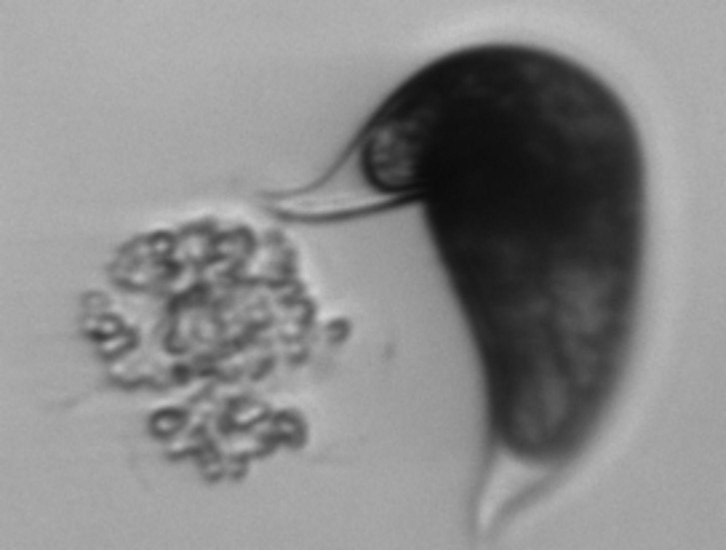

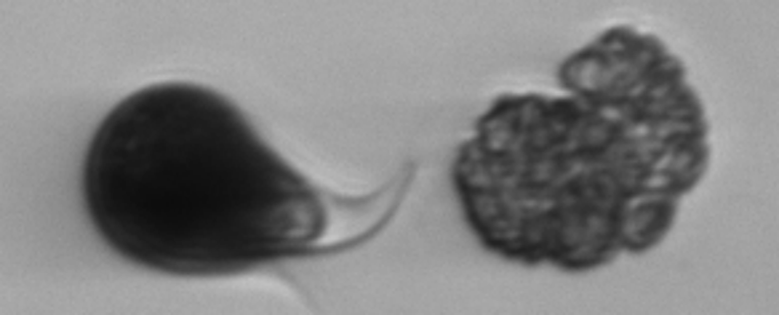

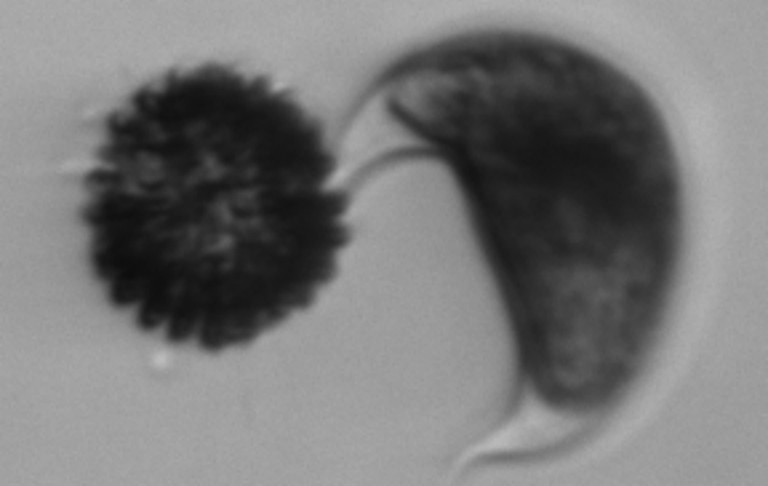

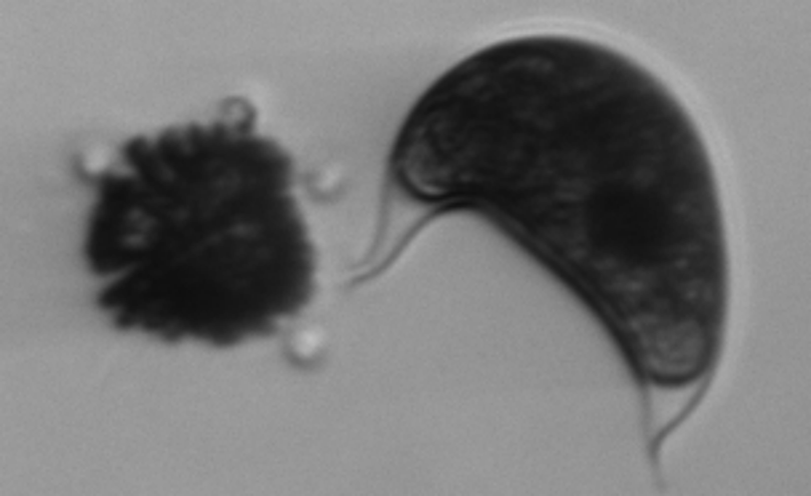

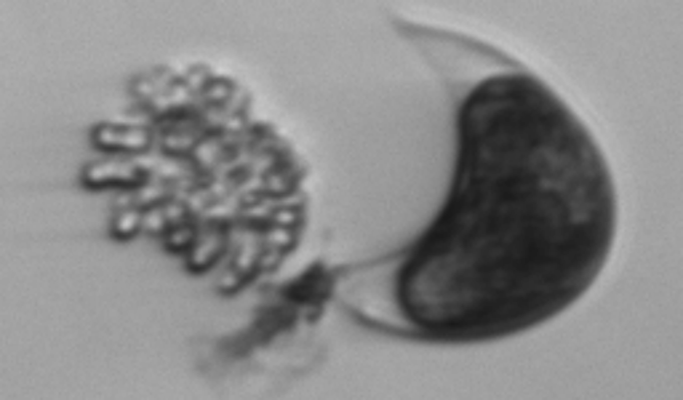

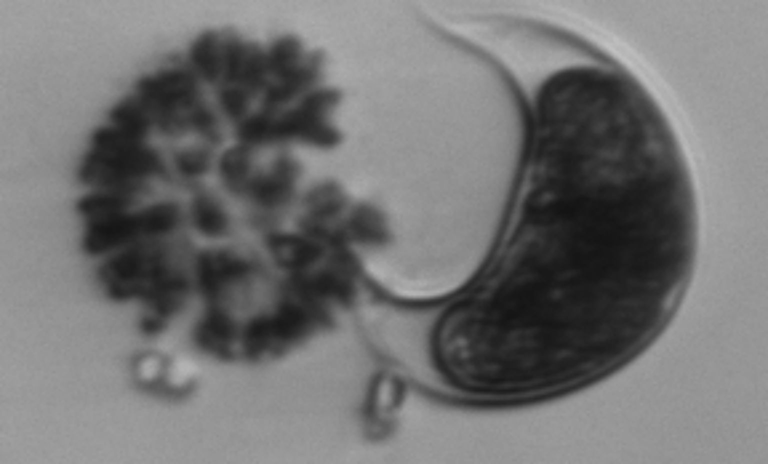

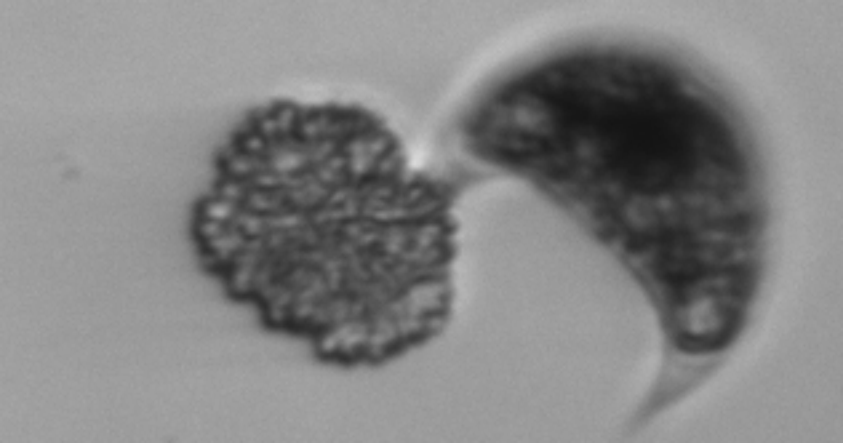

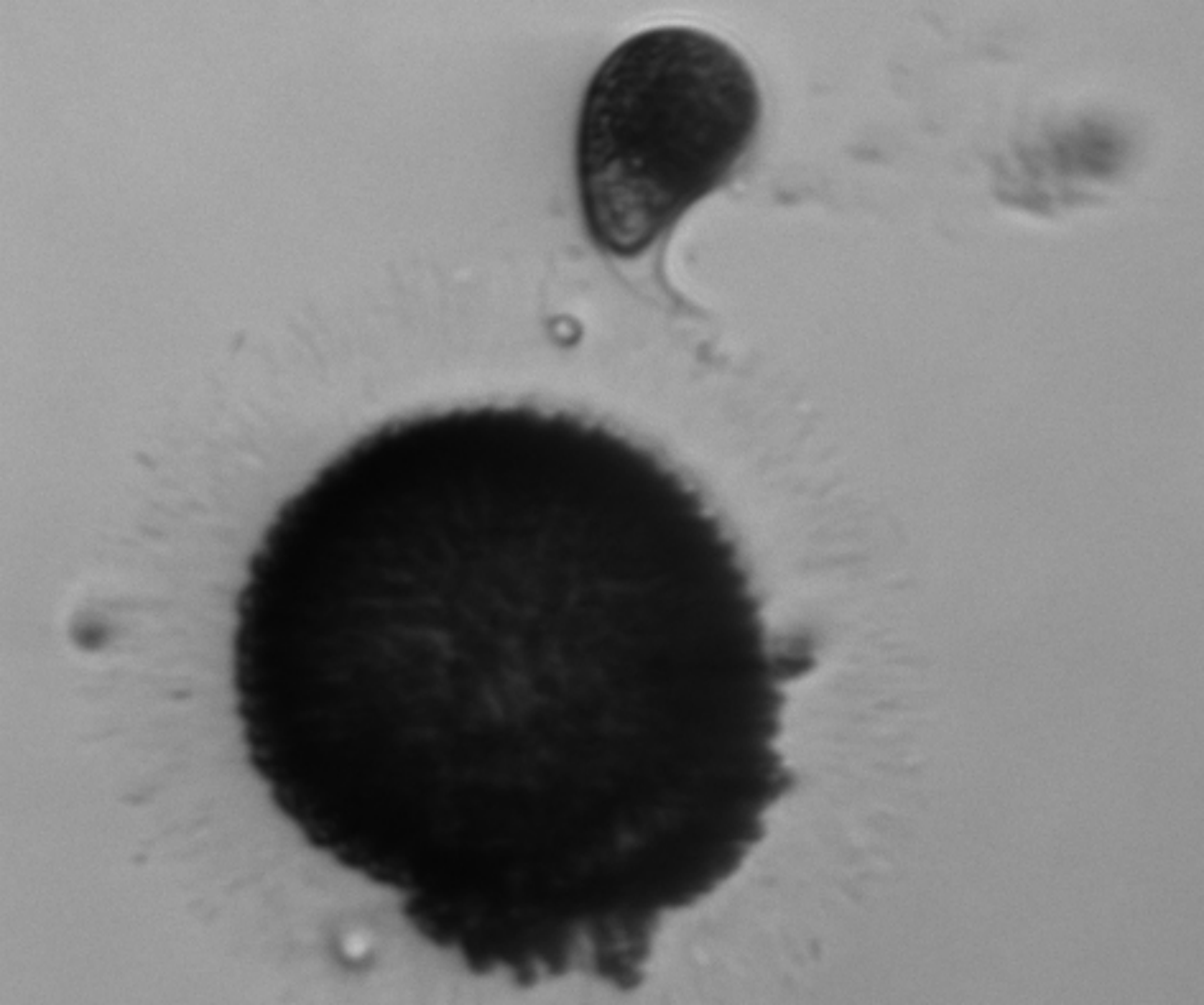

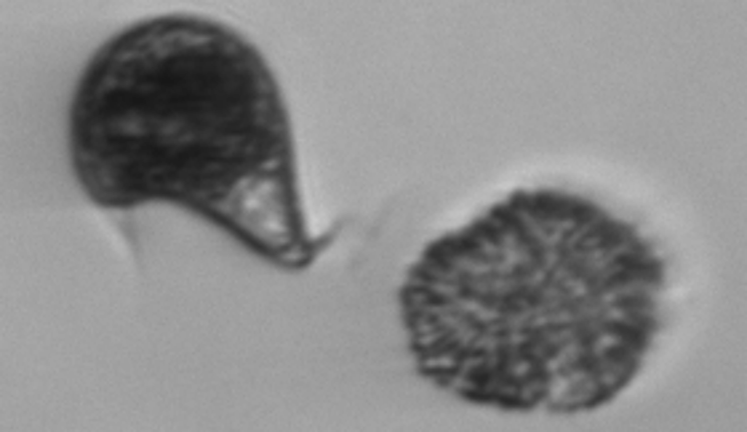

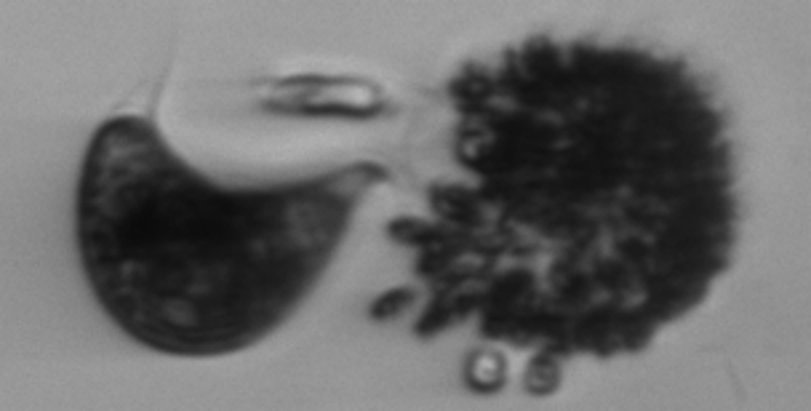

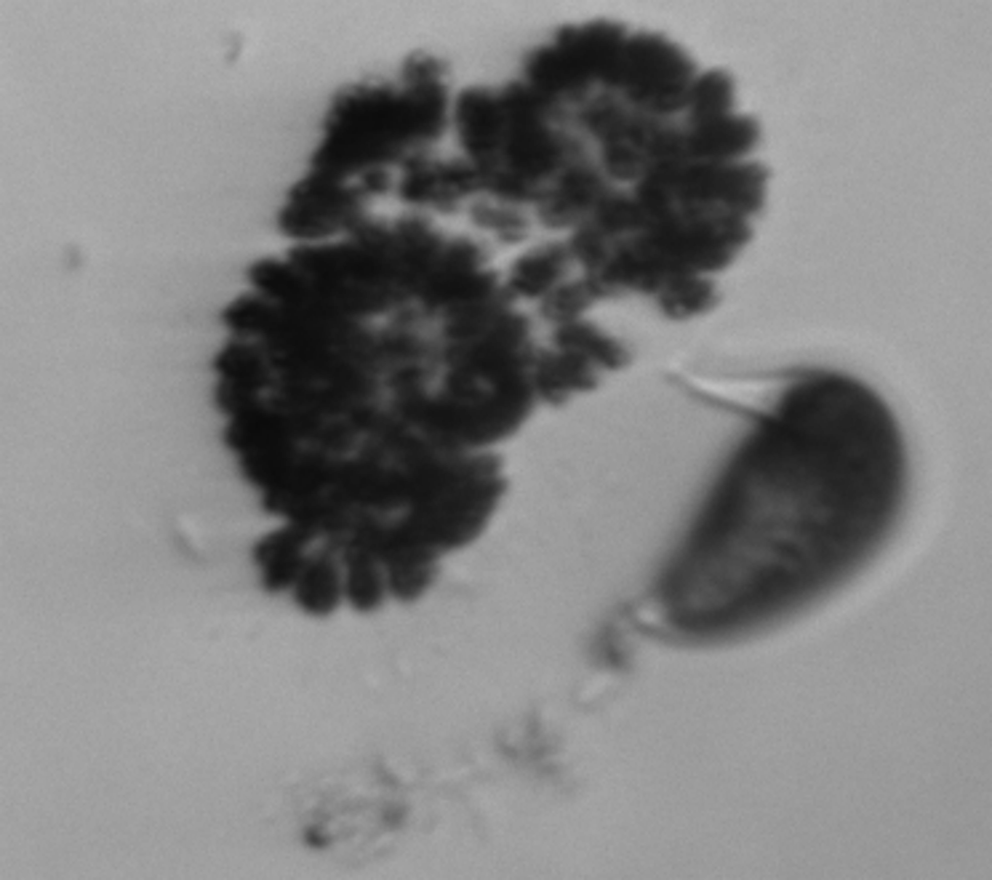

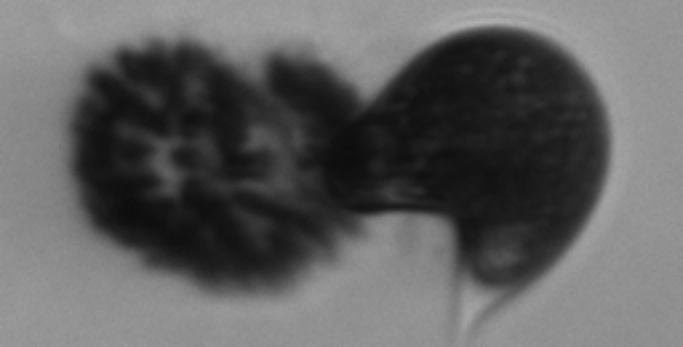

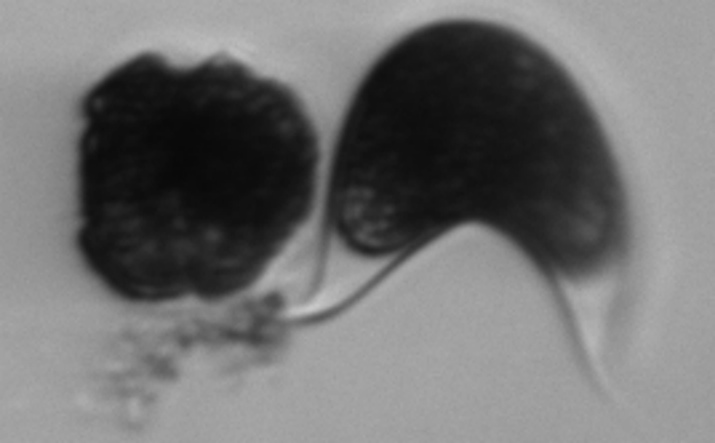

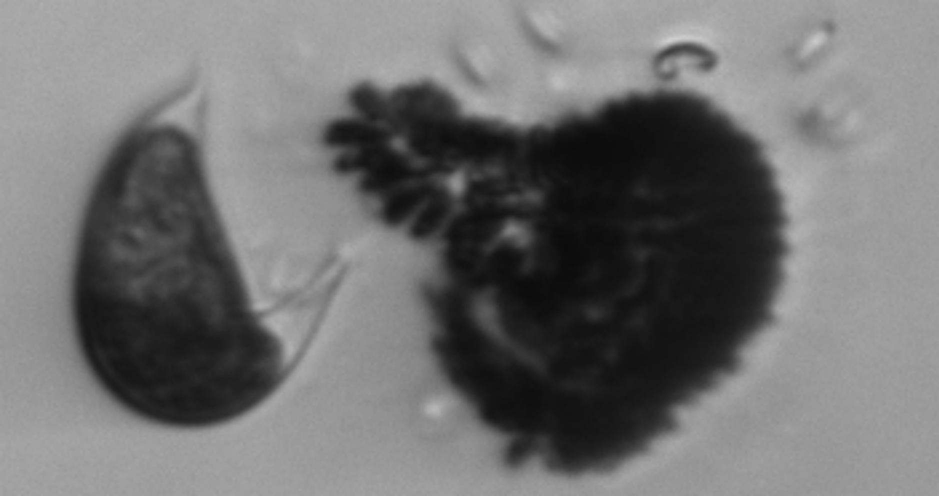

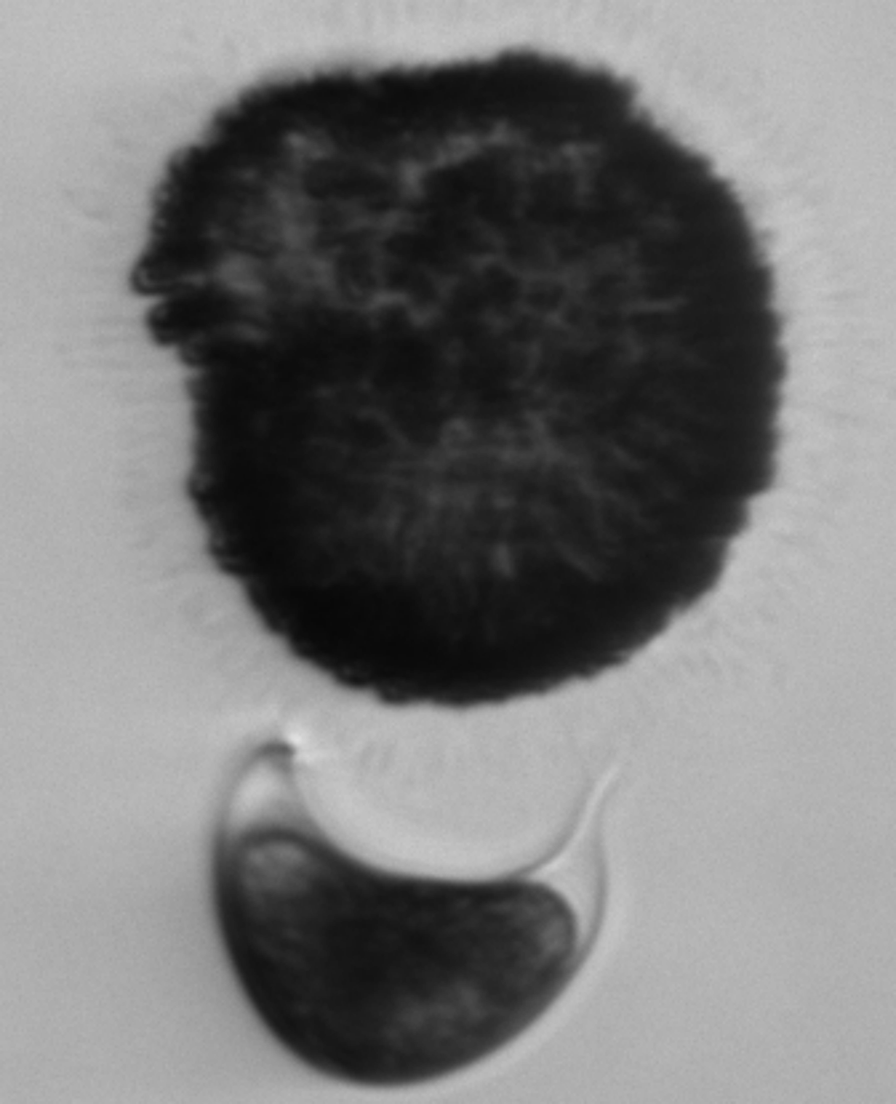

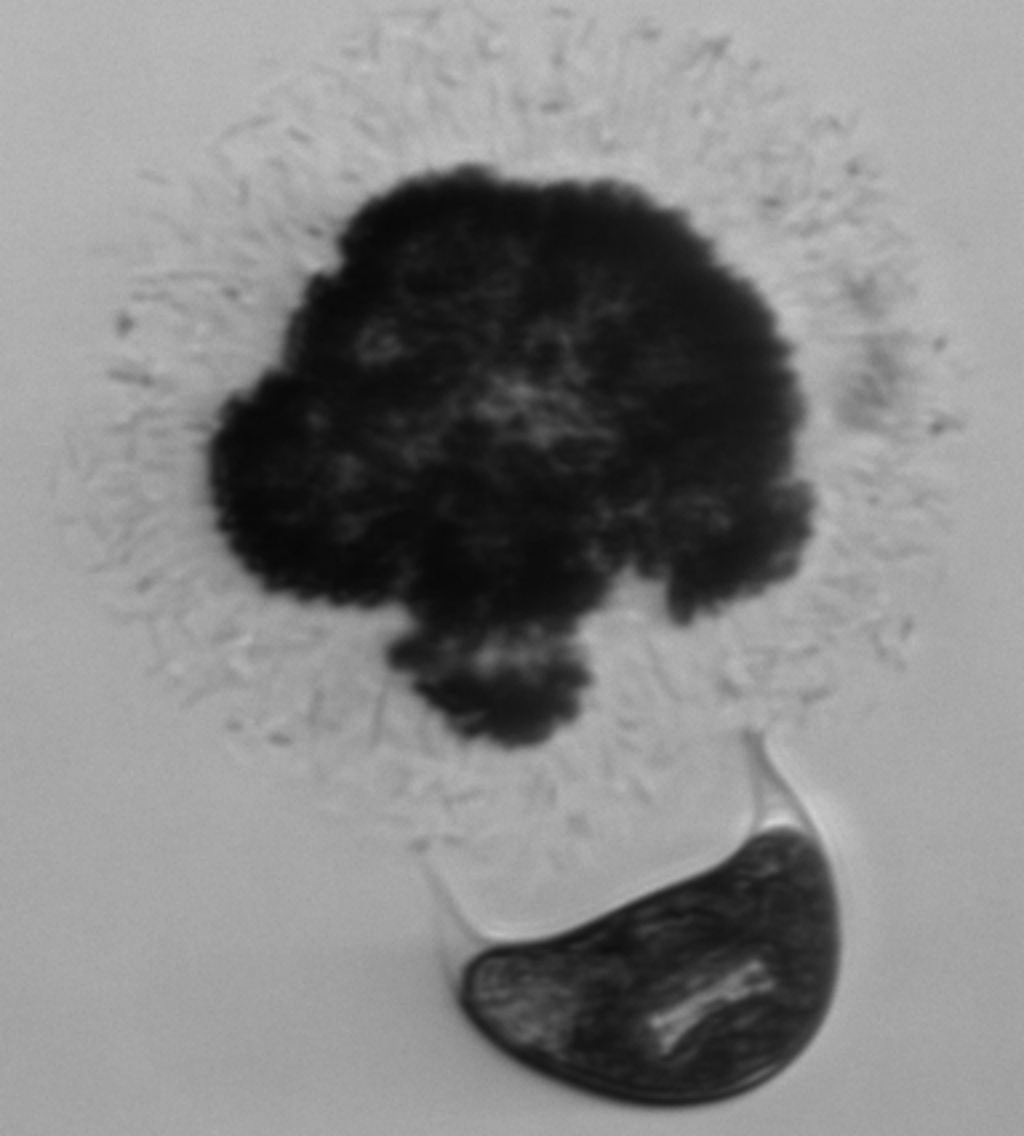

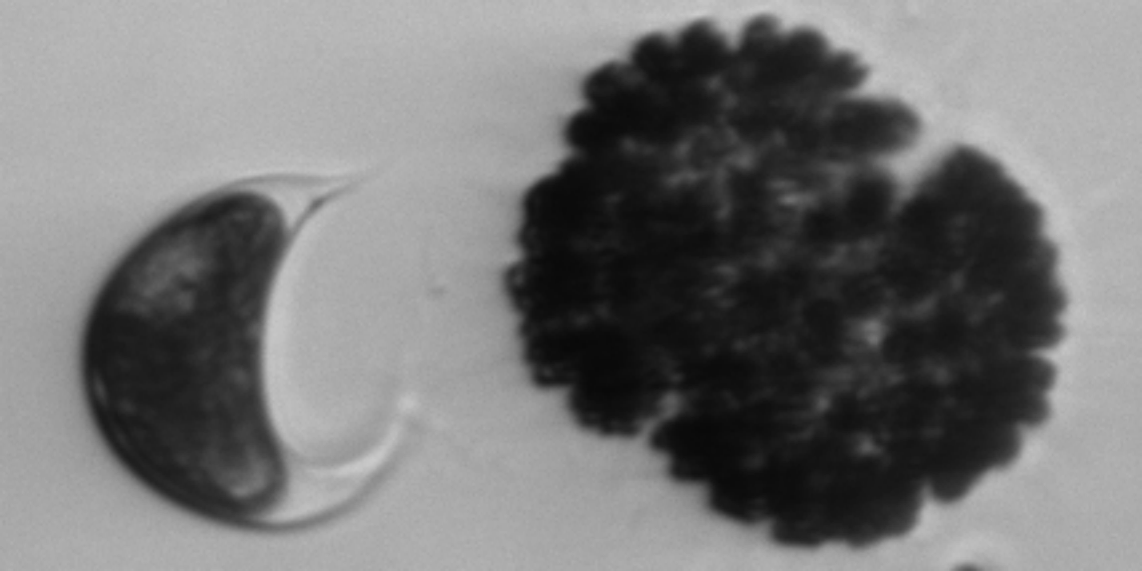

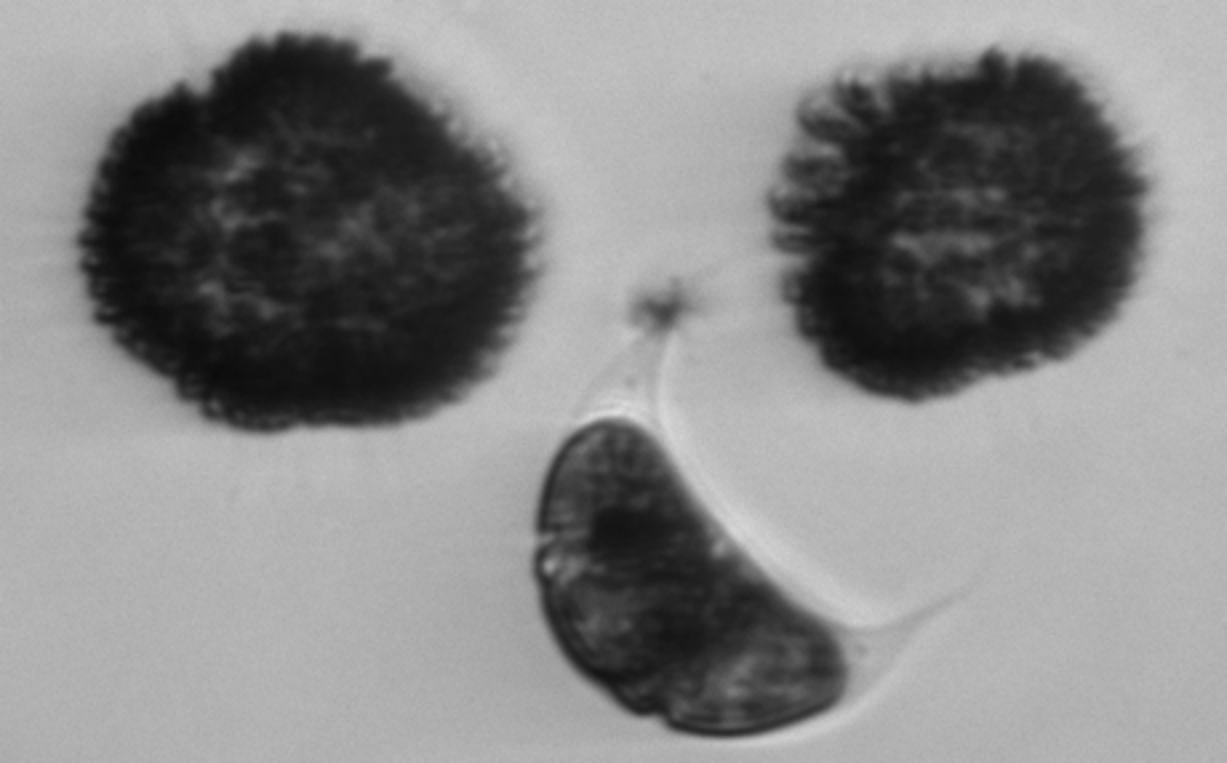

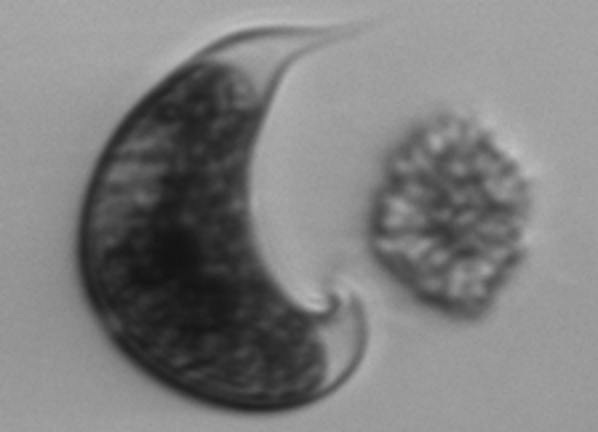

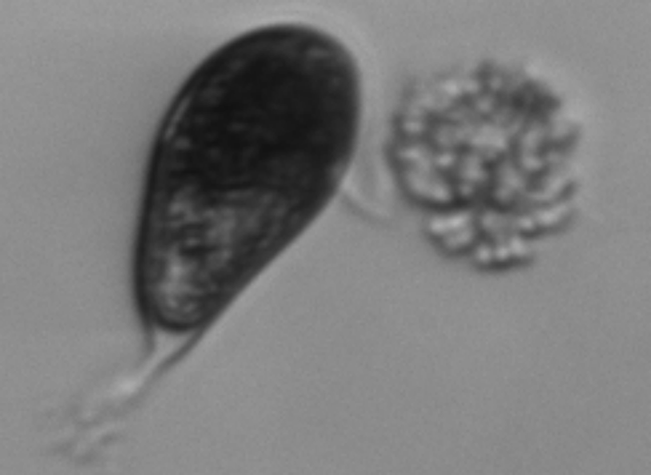

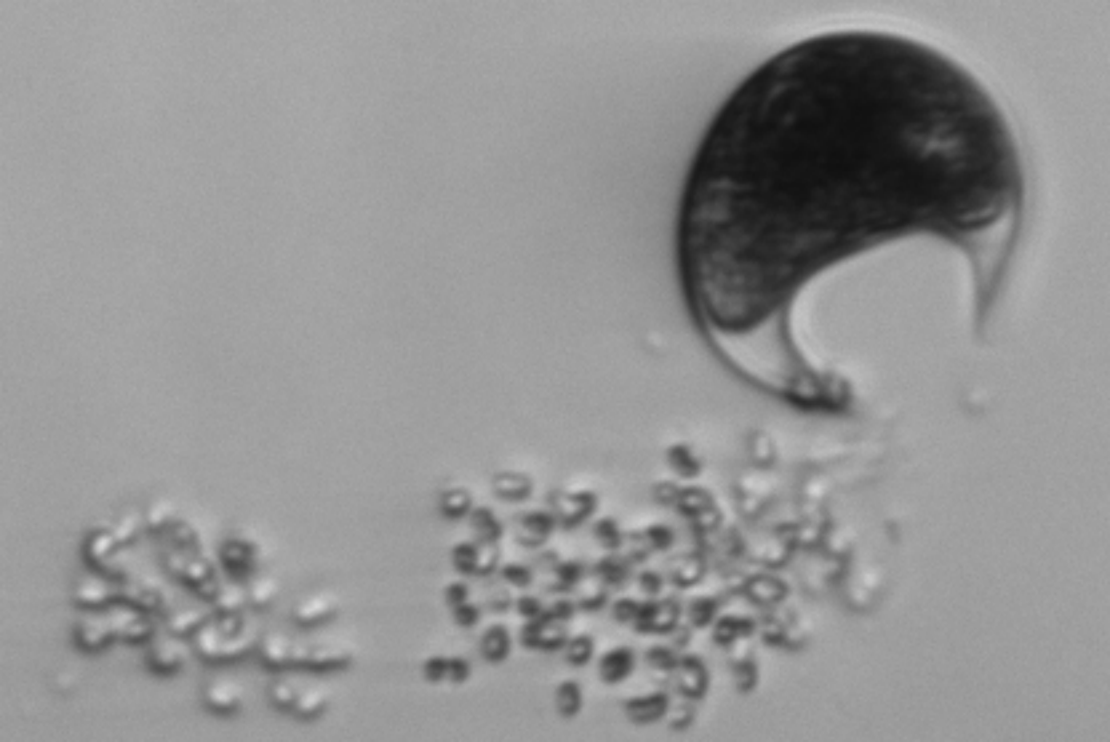

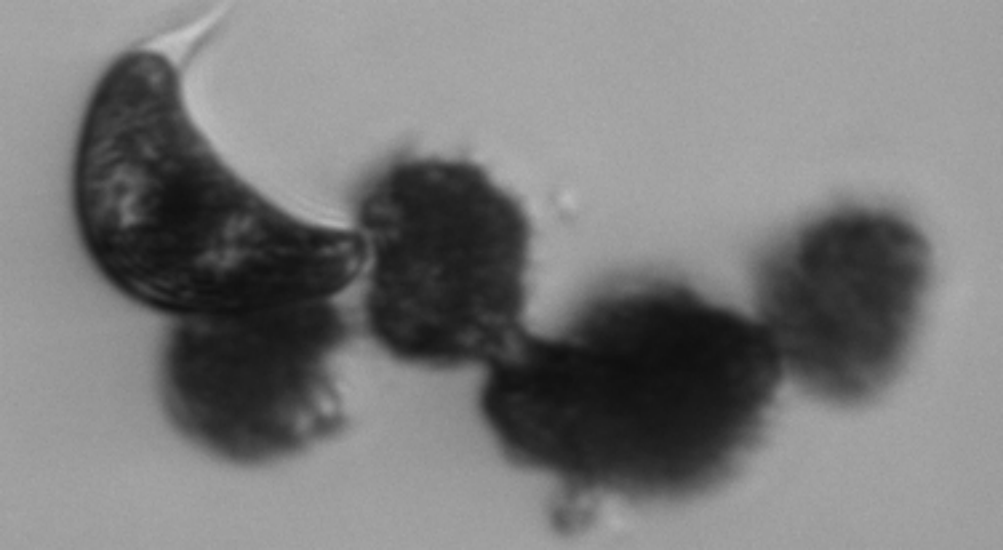

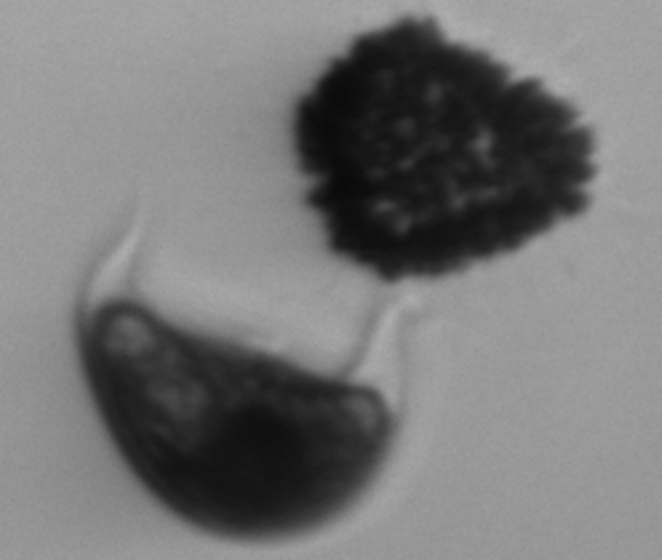

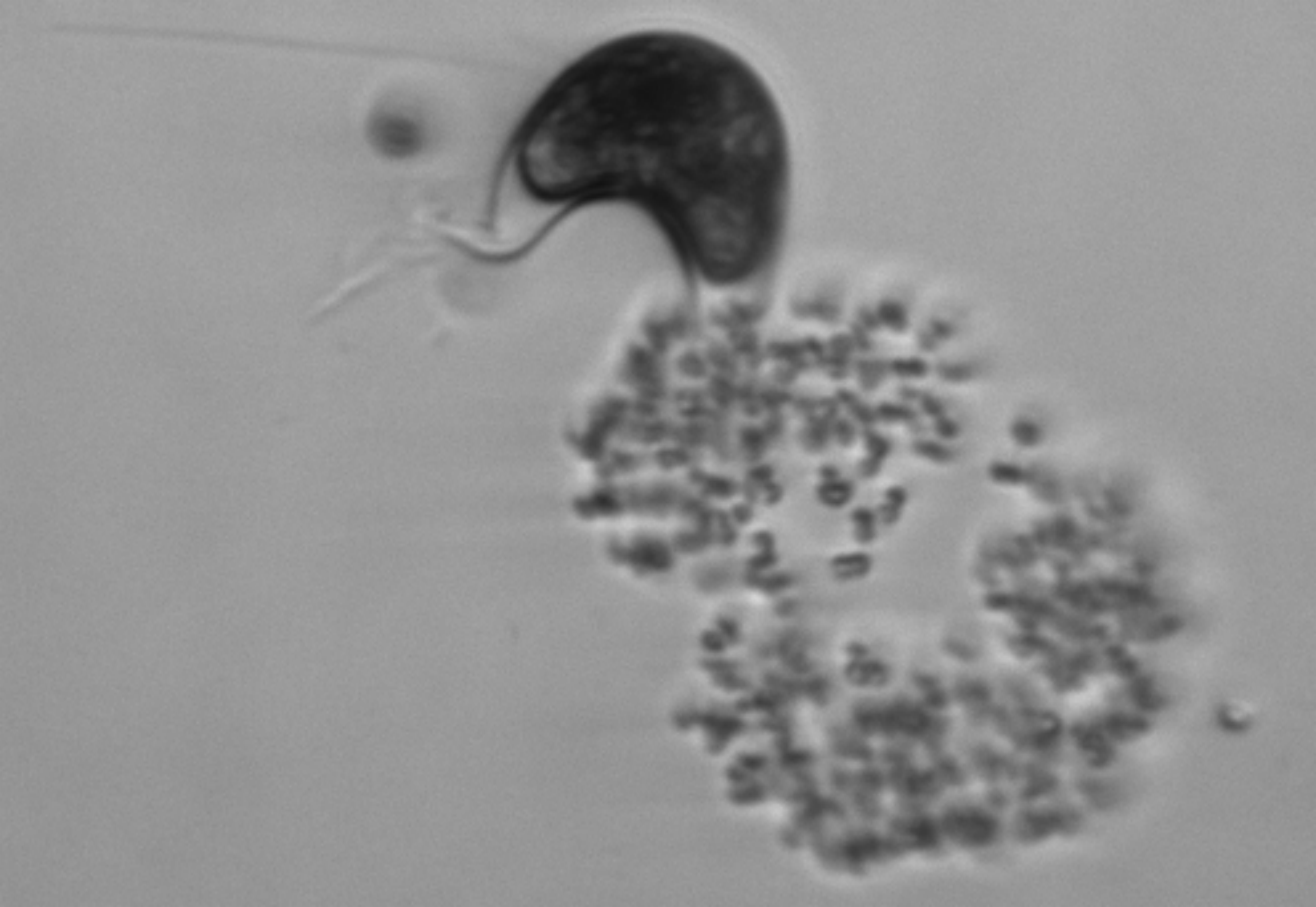

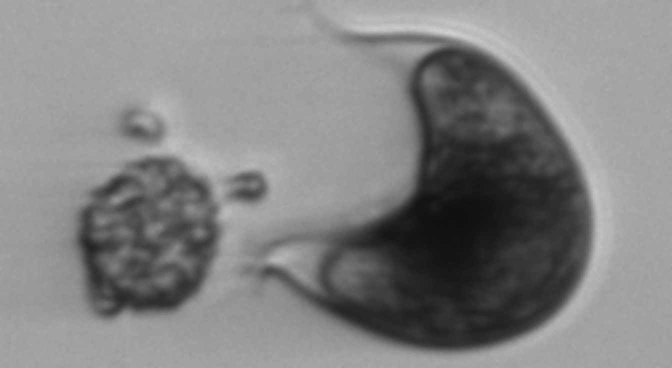

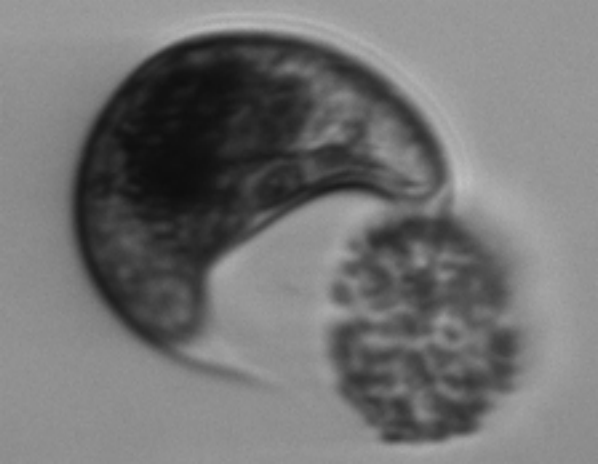

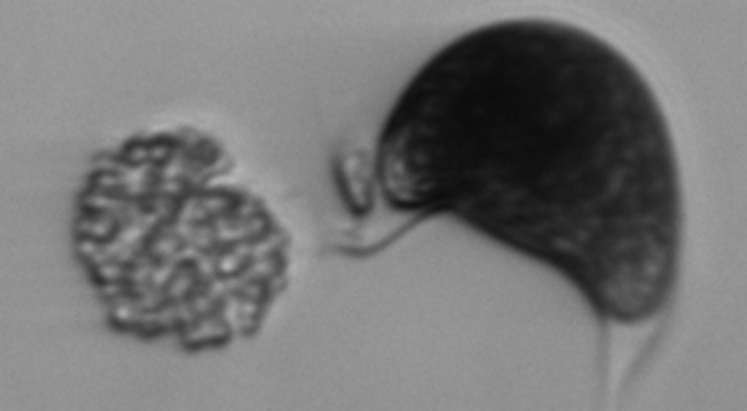

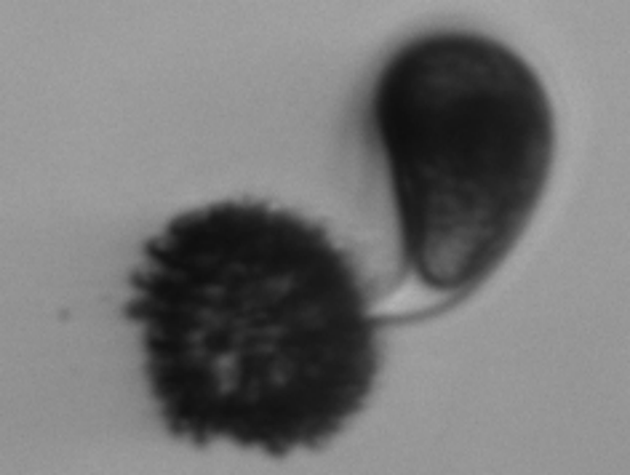

Supplement: Cystodinium_and_its_attachments-Dolichospermum_label_fbab025 [file cystodinium_and_its_attachments-dolichospermum_label_fbab025.zip › Cystodinium_and_its_attachments-Dolichospermum_label_fbab025.pdf]
