# Supplementary material for: The private life of Cystodinium: in situ observation of its attachments and population dynamics
Source: J Plankton Res. 2021 Apr 19;43(3):492–6. doi: 10.1093/plankt/fbab025 (PMC8163037; doi:10.1093/plankt/fbab025)
Supplement: Cystodinium_supplemental_2021_submission_fbab025 [file cystodinium_supplemental_2021_submission_fbab025.zip › Cystodinium_supplemental_2021_submission_fbab025.docx]

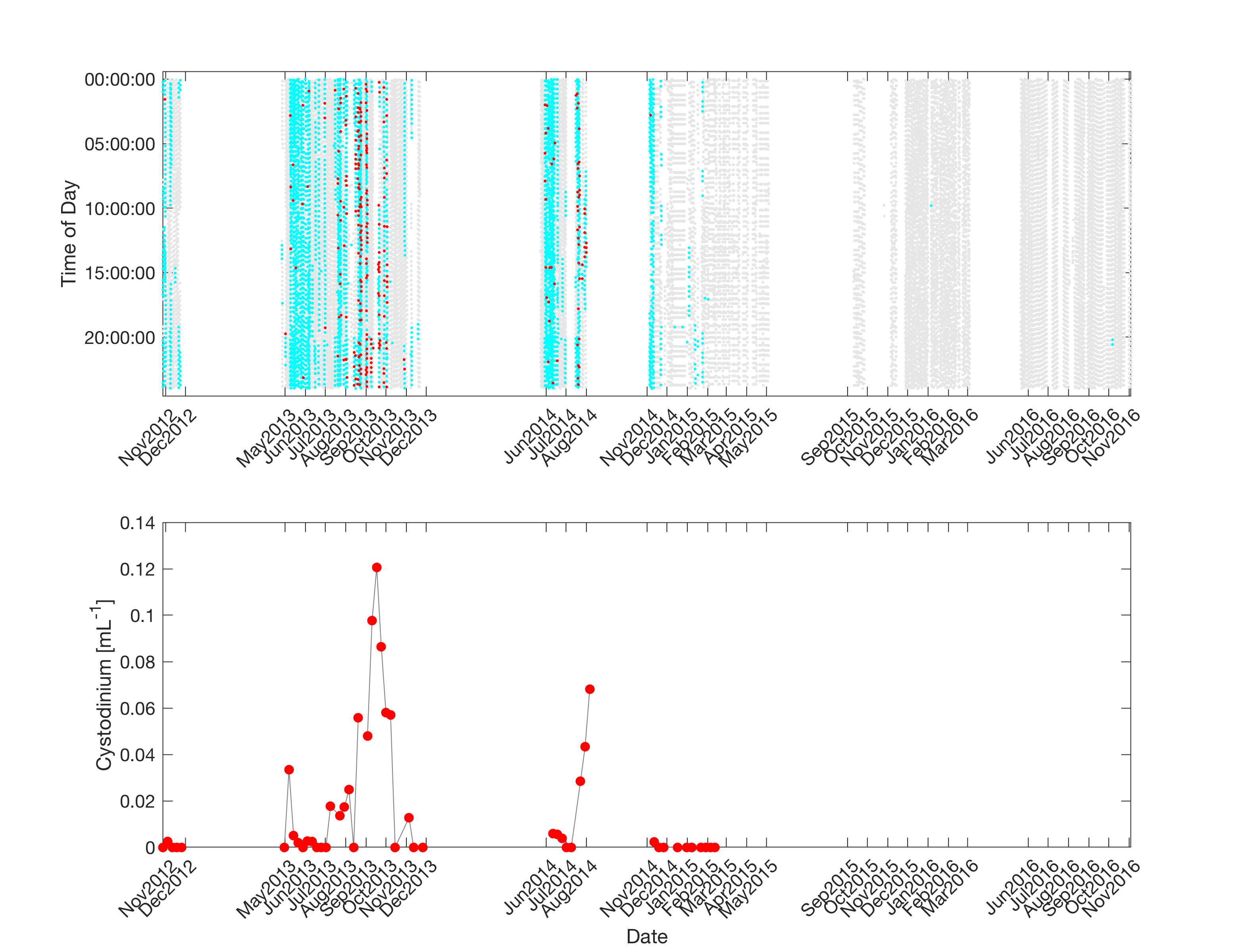


**Supplemental Figure 1.** Upper plot: distribution of *Cystodinium* cyst detections (red), manually-classified (blue) and full (grey) datasets. Lower plot: *Cystodinium* cyst detections per volume of manually-classified sample.


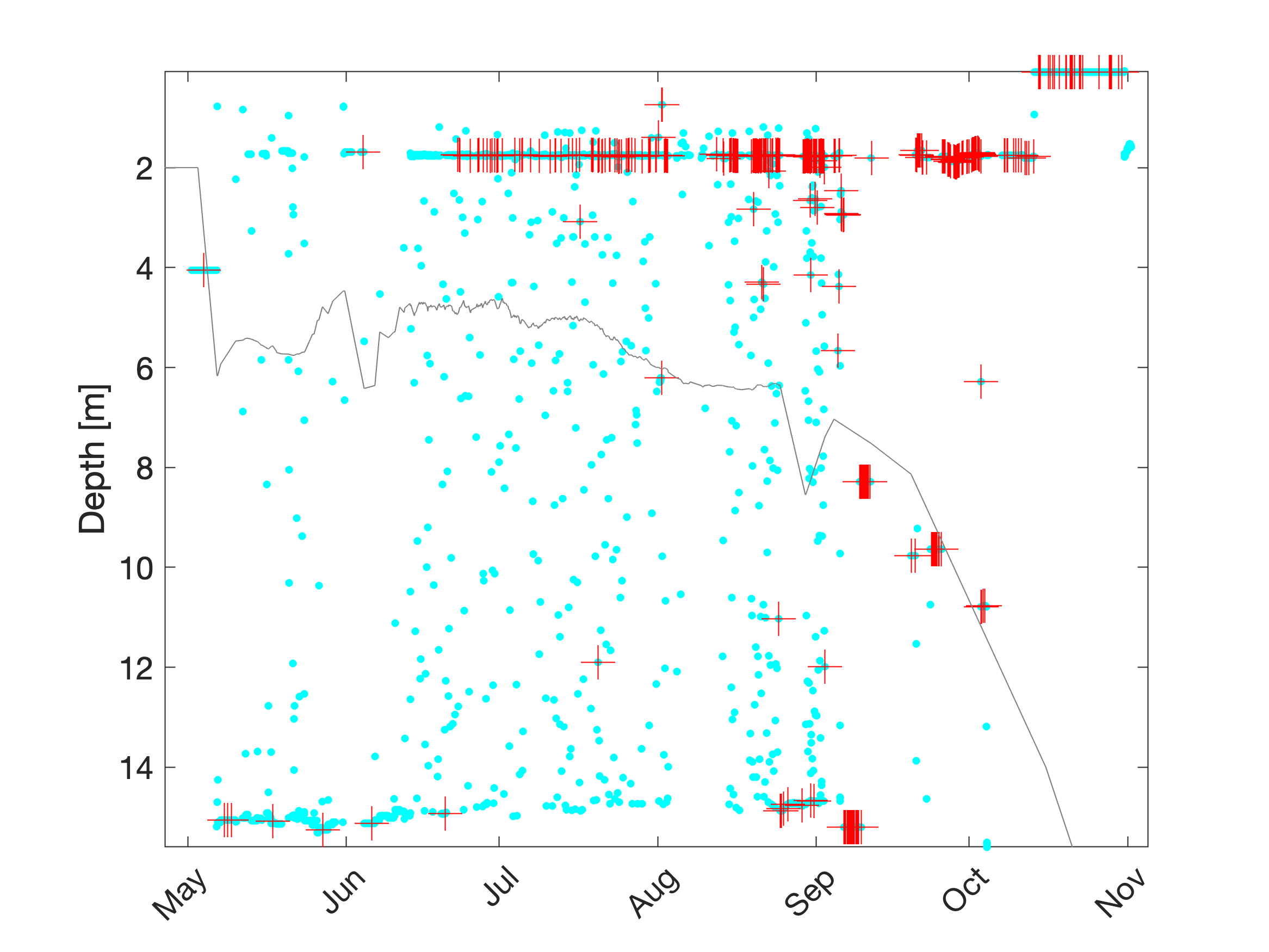


**Supplemental Figure 2.** Depth distribution of 2013 IFCB samples (cyan circles) and *Cystodinium* cyst detections (red crosses). The 7‑day average seasonal thermocline is indicated in grey. Note the May and early June samples were taken mostly around 15 m depth while later samples are concentrated around 2 m.

**Supplemental information**

The seasonal thermocline was extracted using a Lake Analyser algorithm. A 7-day centered average was applied, and the result was linearly interpolated and resampled to match the IFCB phytoplankton sample timepoints. Thermocline estimates were unavailable for the endpoints of 2013 (April 26 – May 5 & August 31 – 21 November); for these points we estimated the seasonal thermocline using measurements from a hand-held YSI 600XL.
